# Acridane[4]Arenes: Scope of the Macro‐Tetramerization, Derivatization Options, and Water‐Soluble Derivatives

**DOI:** 10.1002/chem.202503091

**Published:** 2025-11-28

**Authors:** Vera Höft, Jonathan Pfeuffer‐Rooschüz, Ricard López‐Coll, Alessandro Prescimone, Konrad Tiefenbacher

**Affiliations:** ^1^ Department of Chemistry University of Basel Basel Switzerland; ^2^ Department of Biosystems Science and Engineering ETH Zurich Basel Switzerland

**Keywords:** host–guest systems, macrocycles, self‐assembly, supramolecular chemistry, water‐soluble

## Abstract

Macrocycles are essential scaffolds in supramolecular chemistry, offering versatile platforms for functionalization. Here, we report the optimization, expanded scope, and comprehensive derivatization strategies for acridane[4]arenes (A4A), a large macrocyclic platform featuring a conformationally restricted bowl shape analogous to resorcin[4]arene. The scope of the macrocyclization was expanded with four distinct ‘feet’ substituents at the lower rim. Strategies for selective functionalization at the upper rim were developed, including *N*‐Boc protection at each acridane subunit. This modification induced self‐assembly into a dimeric cage in apolar solvents, which form stable host–guest complexes with tetrabutylammonium salts. Further functionalization of the acridane nitrogen via Buchwald–Hartwig cross‐coupling allowed installation of aromatic groups bearing diverse functional handles. Finally, water‐soluble A4A derivatives were achieved by introducing charged ionic ‘feet’ substituents, such as 1‐methylimidazolium. These results establish A4As as a highly modular macrocyclic platform with broad potential for supramolecular applications.

## Graphical abstract

Acridane[4]arenes (A4As) are introduced as a versatile, conformationally restricted macrocyclic platform accessible in six steps and good yields. Expanded lower‐rim scope, selective upper‐rim functionalization, and N‐Boc modification enable controlled self‐assembly into a dimeric cage that binds tetrabutylammonium salts. Further Buchwald–Hartwig diversification and ionic substituents afford water‐soluble derivatives, establishing A4As as a broadly modular scaffold for supramolecular applications.

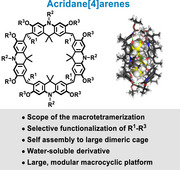

## 1 Introduction

**FIGURE 1:**
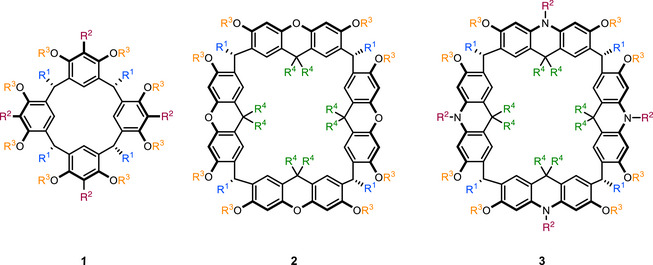
Derivatization sites of resorcin[4]arene 1, xanthene[4]arene 2, and acridane[4]arene 3.

Supramolecular chemistry is intimately linked to macrocycles, not only due to the historical context [1, 2, 3], but because they are key building blocks for many research areas [4, 5, 6]. Thus, it is not surprising that interest in the development of larger macrocycles has surged over the last couple of years [7, 8, 9, 10, 11, 12, 13, 14, 15, 16, 17, 18, 19, 20, 21, 22, 23, 24, 25, 26, 27, 28, 29, 30]. However, only a small subset of these newly developed macrocycles feature a conformationally restricted bowl shape similar to resorcin[4]arene **1** (Figure 1), a true workhorse for the construction of diverse molecular containers [31, 32, 33, 34, 35, 36]. What makes resorcin[4]arene especially well‐suited for such endeavors? (1) Its conformationally restricted, well‐defined geometry is suitable for self‐assembly [37, 38] and the construction of larger concave containers [39, 40, 41, 42]. (2) The phenol moieties at the upper rim can be readily modified via substitution reactions. (3) The ‘feet’ at the lower rim (R^1^, Figure 1) enable a facile tuning of the solubility properties. For instance, the attachment of longer alkyl chains (*n*‐undecyl) entails good solubility in apolar organic solvents, while charged residues lead to water‐soluble derivatives [43, 44, 45]. Interestingly, most large macrocycles developed do not feature these properties. Thus, our group set out to overcome this limitation over the last few years. It was shown that the simple expansion of the resorcinol unit to the enlarged naphthalene derivative failed to deliver bowl‐shaped macrocycles [46, 47, 48, 49]. The expansion of resorcin[4]arene to higher resorcin[n]arene oligomers is in principle possible. However, the derivatives known do not feature R^1^ `feet` at the lower rim, making them conformationally more flexible and less soluble in apolar solvents [50, 51, 52]. Moreover, derivatives larger than resorcin[5]arene do not feature a symmetric cone conformation anymore [53, 54]. To our knowledge, only two larger counterparts to resorcin[4]arene have been reported that fulfill all the three advantages mentioned above: The xanthene[4]arene **2** ^19^ and the acridane[4]arene **3** ^22^ (Figure 1). The xanthene[4]arene **2** turned out to be thermodynamically unstable, and the formation of its smaller trimeric oligomer dominated under most conditions. Thus, the yields obtained were rather low (4%–7% for the macro‐tetramerization), making it less suited for further investigations [19]. Thus, it was a pleasant surprise to see high yields of 53%–83% for the macro‐tetramerization to acridane[4]arene **3** [22]. The preference for acridane[4]arene is attributed to its higher thermodynamic stability and the reversibility of the reaction, which drives the equilibrium of initial mixtures predominantly toward the cyclotetramer. In our initial report, we synthesized two acridan[4]arenes featuring either *i*‐butyl or *n*‐undecyl R^1^ ‘feet’ [22].

This work aimed at unlocking the full potential of this novel, conformationally restricted, large macrocycle by exploring the derivatization options at positions R^1^‐R^4^. Specifically, we investigated (1) the scope and limitations of the R^1^ and R^4^ substituents during the macro‐tetramerization reaction, (2) a strategy to functionalize either R^2^ or R^3^ selectively on the formed macrocycle, (3) the possibility of synthesizing derivatives that are soluble in aqueous mixtures, and (4) options to further improve the synthetic sequence to these macrocycles.

## 2 Results and Discussion

### 2.1 Optimization of the Reaction Sequence and Synthesis of Acridanes with Different R^4^ Groups

**SCHEME 1:**
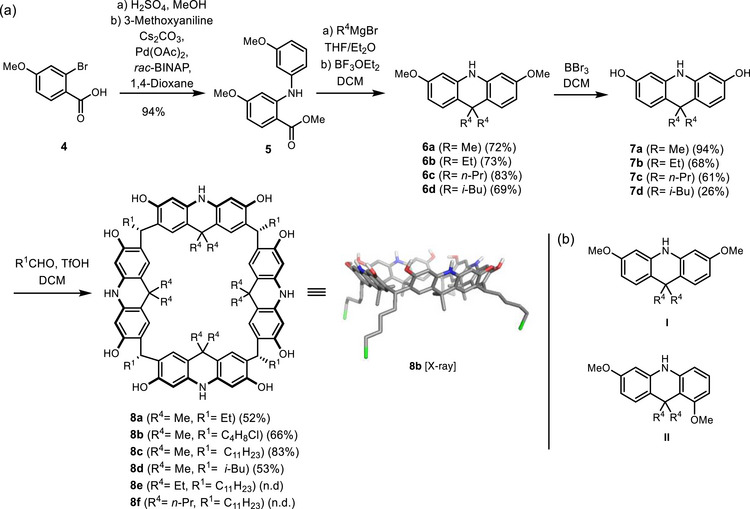
(a) Synthesis of acridane[4]arene derivatives **8a** – **8f**, (b) Isomers **I** and **II**, which are formed during the synthesis of **6a** – **6d**.

The acridane[4]arenes (A4A) were synthesized in six steps from commercially available, inexpensive 2‐bromo‐4‐methoxybenzoic acid **4** (Scheme 1a). In our initial route [22], the first two steps represented a bottleneck in the sequence: (1) the esterification had to be performed in a microwave using methyl iodide, limiting upscaling, and (2) the Ullman coupling gave the product **5** in rather modest yield of 65% over 2 steps. Thus, it was decided to optimize these two steps. We found that the carboxylic acid can easily be esterified quantitatively with sulfuric acid and methanol, followed by a Buchwald–Hartwig cross‐coupling to give **5** in an excellent yield (94% over 2 steps). The R^4^ substituents were quantitatively introduced by a Grignard addition to the ester, from which the protected acridanes **6a**–**6d** were obtained by a Lewis acid‐catalyzed electrophilic aromatic substitution. The reaction yielded two constitutional isomers, I and II (Scheme 1b), with the ratio formed depending on the substituent R^4^. For the methyl‐substituted derivative (**6a**), a ratio of 3.0:1.0 (I:II) was observed, affording the desired isomer I in an isolated yield of 72% over two steps. Modification of the reaction conditions did not significantly affect this ratio (see Supporting Information Chapter 2). However, extension to larger alkyl substituents (ethyl and *n*‐propyl) shifted the ratio further toward the desired isomer I (4.7:1.0 and 4.2:1.0, respectively). The synthesis of the *i*‐butyl substituted acridane **6d** required higher temperatures and longer reaction times, but also afforded **6d** in good yield (69%).

It was found that the length of the R^4^ substituents influences the stability of the acridanes, as reflected in the yields of the subsequent methyl deprotection with boron tribromide to afford **7a**–**7d**. Yields decreased with increasing R^4^ substituent size, from 94% for **7a** to 68% and 61% for **7b** and **7c**, respectively, and to 26% for the *i*‐butyl derivative **7d** due to decomposition.

### 2.2 Scope of the Macro‐ Tetramerization Reaction

To explore the scope of the macro‐tetramerization reaction and to create additional functional handles in the macrocycle, we investigated four R^1^ groups (ethyl, 4‐chlorobutyl, undecyl, and *i*‐butyl). These groups are introduced during the macro‐tetramerization itself (**7** → **8**, Scheme 1a). Using the most stable acridane **7a** (R^4^ = Me), we accessed all four R^1^‐derivatives of the A4A (**8a**–**d**) in good to excellent yields (52%–83%), likely due to equilibration of the reaction mixture to the thermodynamically stable tetrameric macrocycle. Reaction monitoring of the macrocyclization leading to **8a** revealed the initial formation of both trimeric and tetrameric species. Over time, the trimer was gradually converted into the more stable tetramer, as confirmed by NMR and HRMS analyses (see Supporting Information, Chapter 3). Further evidence for reversibility and thermodynamic control was obtained from the reaction's dependence on water: addition of molecular sieves to remove water completely suppressed macrocycle preference, indicating that significant product formation requires reversible equilibration to achieve thermodynamic control. Macrocyclization with propionaldehyde and **7a**, followed by washing with ethyl acetate, gave macrocycle **8a** with a short R^1^ substituent in good purity and yield (52%). In addition to alkyl substituents, we prepared the chloro‐functionalized derivative **8b** by reacting **7a** with 5‐chloropentanal. After recrystallization from dioxane, macrocycle **8b** was obtained in good yield (66%). Single crystals suitable for X‐ray crystallography were produced by slow evaporation of a solution of **8b **in acetone at 4 °C (see Supporting Information Chapter 6). The crystal structure analysis confirms the same crown conformation as previously reported for **8d** [22]. Attempts to introduce R^1^‐hydroxy substituents using 2,3‐dihydrofuran or 3,4‐dihydropyran under a range of conditions [43, 55], yielded only trace amounts of the desired products. Accordingly, the chloro‐substituted derivative **8b** was employed as a suitable alternative. The undecyl and *i*‐butyl derivatives were already described in our preliminary communication [22] and were purified by recrystallization from ethyl acetate.

Furthermore, we explored the cyclization of the other, less stable, acridane derivatives **7b**–**d** (R^4^ = Et, *n*‐Pr, *i*‐Bu). These were of interest to us as the R^4^ substituent may substantially affect the stability and solubility of the macrocycle, and potentially could also lead to larger, pentameric macrocycles in case of bulky R^4^ substituents, as observed for the related xanthenearenes [19]. However, as expected, the less stable acridanes **7b**–**d** carrying longer R^4^ groups did not perform well in the cyclization reaction. Only two further A4A were obtained (**8e**,**f**). However, their inherent instability precluded isolation in analytically pure form, as decomposition occurred during purification on silica and size‐exclusion chromatography. Attempts at crystallization from various solvents were unsuccessful due to the poor crystallinity of the material.

### 2.3 Selective Functionalization on the A4A Rim

**SCHEME 2:**
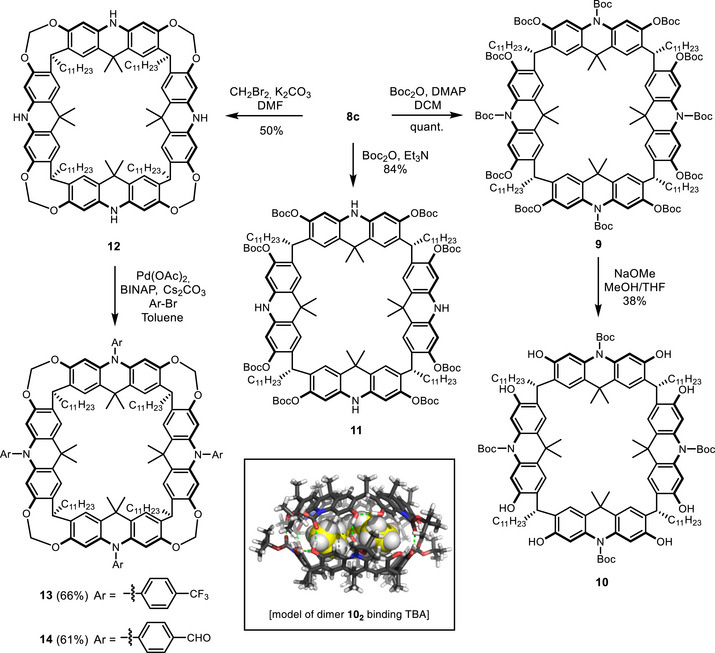
Selective functionalization on the A4A rim of 8c. Molecular model of the dimeric cage structure 10_2_ binding tetrabutylammonium (TBA).

After having established access to a wider selection of A4A, we explored options to functionalize the acridane nitrogen (R^2^) and the phenols (R^3^) at the upper rim of the macrocycle. Acridane[4]arene **8c** was chosen for these studies due to its high stability and good solubility in a range of solvents. Boc protection with triethylamine as base efficiently differentiated phenolic and acridane nitrogen sites, affording **11** in 84% yield, whereas acetyl protection was less effective. With 4‐dimethylaminopyridine as a base, both the acridane nitrogen and phenolic groups were Boc‐protected quantitatively to produce **9** (Scheme 2). Subsequent treatment of macrocycle **9** with NaOMe selectively removed the phenolic protecting groups, affording the *N*‐Boc macrocycle **10** in a useful yield of 38%. On the other hand, the use of triethylamine as a base, led to the selective protection of the phenols in an excellent yield (84%).

Furthermore, the acridane nitrogen can be coupled to aromatic moieties via Buchwald–Hartwig cross‐coupling. After protecting the phenols of undecyl‐acridan[4]arene **8c** with methylene bridges to give compound **12**, macrocycle **13** (bearing a CF_3_ group) and macrocycle **14** (bearing an aldehyde group) were obtained in good yields of 66% and 61%, respectively (Scheme 2). We found that this reaction proceeded particularly well with electron‐withdrawing substituents in the para position, whereas coupling of unsubstituted bromobenzene afforded only trace amounts of product. Those functional groups, like the aldehyde, offer the possibility for further functionalization.

### 2.4 Self‐Assembly of 10 to a Dimer Cage Structure

Further ^1^H NMR studies of macrocycle **10** revealed that *N*‐Boc‐protection induces self‐assembly into a larger structure in apolar solvents such as chloroform. This behavior is reflected in the broadening and emergence of new signals, in contrast to the sharp resonances observed in a polar solvent such as acetone. In comparison, the *N*,*O*‐Boc macrocycle **9** does not undergo assembly under these conditions, as no signal broadening was observed in chloroform. More information about the size of the formed assembly of **10** was gained by DOSY‐NMR measurements, which have been established as a reliable tool for determining the size of supramolecular structures [56]. A diffusion coefficient of 0.19 ×10^−5^ cm^2^s^−1^ in chloroform‐*d* at 298K was obtained from all major signals, corresponding to a hydrodynamic radius of 25.8 Å as estimated using the modified Stokes–Einstein equation. This radius was consistent with a molecular model of the dimeric species, which yielded an estimated hydrodynamic radius of 25 Å (see Supporting Information Chapter 5.1). For comparison, the diffusion coefficient of the fully protected macrocycle **9**, which is incapable of self‐assembly, was measured under identical conditions and found to be 0.32 × 10^−^⁵ cm^2^ s^−^^1^, which corresponds to a hydrodynamic radius of 22 Å.

Next, we investigated the properties of the dimeric capsule regarding guest uptake. Therefore, different potential guests were screened (Supporting Information Section 4), and the tetrabutylammonium cation was found to be a suitable guest by the appearance of new upfield signals in the ^1^H NMR spectrum, due to the anisotropic effect of the aromatic cavity. The nature of the corresponding anion had no influence on the shape or chemical shift of the signals. The formation of the host–guest complex was observed as slow exchange on the NMR timescale. The binding constant of TBAB was estimated to be at least 6500 M^−^^1^ (see Supporting Information Section 4). Next, the structure of the complex was investigated by DOSY‐NMR. The measured diffusion coefficient of 0.13 ×10^5^ cm^2^s^−1^ is notably low, even lower than that of the empty cage. The reduced diffusion is likely attributable to the encapsulated guest within the dimeric host, leading to an increased effective diffusional cross‐section.

### 2.5 Toward Water‐Soluble A4A Derivatives

Besides the selective functionalization discussed above, we were also interested in exploring acridane[4]arene derivatives that are soluble in aqueous mixtures. Such derivatives may be of interest for diverse applications, ranging, for instance, from the construction of water‐soluble molecular containers [42, 45, 57, 58] to the exploration of interactions with peptides and proteins [59, 60, 61, 62, 63, 64, 65, 66]. In order to form completely stable macrocycles, we decided to functionalize the oxidation–sensitive phenols with methylene linkages. The methylene linkage was chosen to minimize the attachment of apolar residues that reduce water solubility. With this modification, only the sites R^1^ (‘feet’) and R^2^ (nitrogen substituent) remain available for the introduction of water‐solubilizing groups.

**SCHEME 3:**
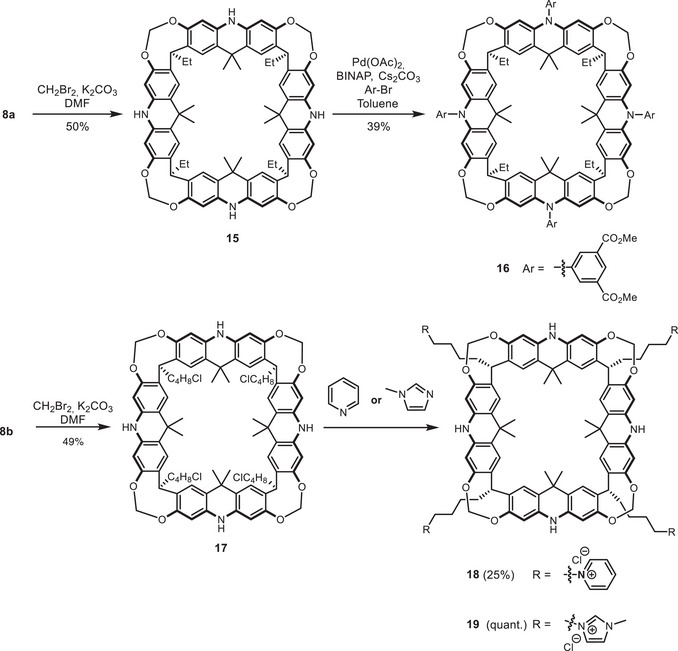
Modification of the acridane nitrogen to increase water solubility.

First, functionalization of the acridane nitrogen (R^2^) was investigated. Direct sulfonation using 1,3‐propanesultone [67] in the presence of various bases led to very complex reaction mixtures, from which no defined product was isolatable. Thus, further *N*‐derivatization focused on the Buchwald–Hartwig cross‐coupling, we established for the lipophilic compounds **13** and **14** carrying undecyl groups at the R^1^ site (Scheme 2). To facilitate water solubility, acridane **8a** (R^1^ = Et) was chosen as the starting point for modification. After protecting the phenols with methylene bridges to obtain macrocycle **15**, (Scheme 3) cross‐coupling with dimethyl 5‐bromoisophthalate yielded compound **16**. Unfortunately, due to the poor solubility of **16** in polar solvents, the ester cleavage under basic conditions did not produce high and reproducible yields. Additionally, despite the presence of eight carboxylic acid groups, water solubility, even in 1:1 DMSO/water mixtures under basic conditions, was not achieved. Therefore, another approach toward water‐soluble macrocycles was explored.

We explored ‘feet’ modifications (R^1^) with charged residues to enhance water solubility. Starting from 4‐chlorobutyl‐acridan[4]arene **8b**, the macrocycle was stabilized via methylene linkages to give **17** in good yield (49%). Pyridinium groups were then introduced by treating **17** with excess pyridine, affording **18**, which displayed moderate solubility in a water/DMSO mixture (10 vol% DMSO). To further increase solubility, pyridine was replaced with 1‐methylimidazole, yielding macrocycle **19**. Due to the higher polarity, macrocycle **19** is readily soluble in water. However, 10 vol% DMSO was necessary to observe NMR resonances, likely by preventing micelle formation and the resulting signal broadening.

## 3 Conclusion

This work establishes A4A as a versatile, conformationally restricted, large macrocyclic platform with multiple modification sites, offering a significantly larger counterpart to the well‐known resorcin[4]arene. The synthetic route to A4As was optimized, enabling access to these macrocycles in six steps with good overall yields. The key tetramerization reactions produced A4A **8a**–**d** in good to excellent yields (52%–83%), with evidence indicating that the tetramer represents the thermodynamically favored product toward which the reaction mixture equilibrates. The scope of the macrocyclization was further expanded by incorporating four different R^1^ groups. Strategies for selective functionalization at the upper rim (R^2^ and R^3^) were developed, including the selective installation of Boc groups at the acridane nitrogen (R^2^), which afforded macrocycle **10** and promoted self‐assembly into a larger dimeric cage structure (**10**_2_) in apolar solvents. This dimeric cage forms a stable host–guest complex with tetrabutylammonium salts. Furthermore, aromatic moieties bearing functional groups were introduced at the acridane nitrogen via Buchwald–Hartwig cross‐coupling, providing additional modification handles. Finally, water‐soluble A4A macrocycles were successfully synthesized. While derivatives bearing eight carboxylic acid groups failed to achieve solubility, the installation of charged ionic R^1^ substituents, specifically 1‐methylimidazole, yielded the readily water‐soluble macrocycle **19**, creating opportunities for diverse applications in host–guest chemistry and beyond. Overall, we are confident that A4As represent a highly versatile macrocyclic platform that will be of interest to scientists for a wide range of applications.

## Conflicts of Interest

The authors declare no conflict of interest.

## Supporting information




**Supporting File 1**: The authors have cited additional references within the Supporting Information[68, 69]. Deposition Number(s) 2495371 data_vh123_150k contain(s) the supplementary crystallographic data for this paper. These data are provided free of charge by the joint Cambridge Crystallographic Data Centre and Fachinformationszentrum Karlsruhe “http://www.ccdc.cam.ac.uk/structures” Access Structures service.

## Data Availability

The data that support the findings of this study are available in the supplementary material of this article. Data for the table and figures have been deposited at https://zenodo.org/records/17552827.
