# Supplementary material for: Acridane[4]Arenes: Scope of the Macro‐Tetramerization, Derivatization Options, and Water‐Soluble Derivatives
Source: Chemistry. 2025 Nov 28;32(1):e03091. doi: 10.1002/chem.202503091 (PMC12759172; doi:10.1002/chem.202503091)
Supplement: Supplementary file 1 — Supporting File 1: The authors have cited additional references within the Supporting Information[68, 69]. Deposition Number(s) 2495371 data_vh123_150k contain(s) the supplementary crystallographic data for this paper. These data are provided free of charge by the joint Cambridge Crystallographic Data Centre and Fachinformationszentrum Karlsruhe “http://www.ccdc.cam.ac.uk/structures” Access Structures service. [file CHEM-32-e03091-s001.pdf]

Acridane[4]arenes: Scope of the Macro-tetramerization, Derivatization Options, and Water-soluble Derivatives

Vera Höft[1], Jonathan Pfeuffer-Rooschüz, Ricard López-Coll, Alessandro Prescimone[1], and Konrad Tiefenbacher*[,1,2]

[1] Department of Chemistry, University of Basel, Mattenstrasse 22, 4058 Basel, Switzerland
[2] Department of Biosystems Science and Engineering, ETH Zurich, Klingelbergstrasse 48, 4056 Basel, Switzerland

Table of Contents

1. GENERAL INFORMATION 3

2. SYNTHESIS 4

Methyl 4-methoxy-2-((3-methoxyphenyl)amino)benzoate (5) 4

3,6-Dimethoxy-9,9-diethyl-9,10-dihydroacridine (6b) 5

3,6-Dimethoxy-9,9-dipropyl-9,10-dihydroacridine (6c) 6

9,9-Diisobutyl-3,6-dimethoxy-9,10-dihydroacridine (6d) 7

9,9-Dimethyl-9,10-acridane-3,6-diol (7a) 8

9,9-Diethyl-9,10-acridane-3,6-diol (7b) 9

9,9-Dipropyl-9,10-acridane-3,6-diol (7c) 10

9,9-Diisobutyl-9,10-acridane-3,6-diol (7d) 11

Ethyl-acridane[4]arene (8a) 12

4-Chlorobutyl-acridane[4]arene (8b) 13

Diethyl-acridane[4]arene (8e) 14

Di-*n*-propyl-acridane[4]arene (8f) 15

***N,O-Boc*-undecyl-acridane[4]arene (9)** 16

***N-Boc*-undecyl-acridane[4]arene (10)** 17

***O-Boc*-undecyl-acridane[4]arene (11)** 18

Methylene-bridged-undecyl-acridane[4]arene (12) 19

***N*-4-(trifluoromethyl)phenyl-undecyl-acridane[4]arene (13)** 20

***N-4*-benzaldehyde-undecyl-acridane[4]arene (14)** 21

Methylene-bridged-ethyl-acridane[4]arene (15) **22**

***N*-5-dimethyl-isophthalate-Ethyl-acridane[4]arene (16)** **23**

Methylene-bridged-4-chlorobutyl-acridane[4]arene (17) **24**

4-Pyridinium-butyl-acridane[4]arene (18) **25**

4-*1*-Methyl-imidazolium-butyl-acridane[4]arene (19) **26**

3. REACTION MONITORING OF THE CYCLISATION TOWARDS ET-A4A (8A) **27**

4. GUEST UPTAKE STUDIES **28**

5. DOSY-NMR STUDIES **30**

5.1 Calculation of the hydrodynamic radius **32**

6. CRYSTALLOGRAPHIC DATA OF CL-A4A (8B) **33**

7. NMR-SPECTRA OF NEW COMPOUNDS **38**

1. General information

Experimental: All reactions involving air- or moisture sensitive substances were carried out under an atmosphere of argon, and the glassware was heated out under vacuum unless otherwise stated.

Analytical methods and instruments: For flash column chromatography separation, a CombiFlash® NextGen 300+ system with silica gel (RediSep® Rf Normal-phase Silica Flash Columns 4 g/12 g/24 g/40 g/80 g) cartridges was used.

Thin-layer chromatography (TLC) was performed with Merk silica gel 60 F254 plates. The probes were analyzed either under UV light (λ = 254 nm) or immersion in a cerium ammonium molybdate solution (CAM) and heat treatment.

NMR spectra were recorded with on a Bruker Ascend 500 spectrometer. As internal references, the respective solvent signals were used: $CDCl_3$ $\delta(^1H)$= 7.26ppm, Acetone-d_6 $\delta(^1H)$= 2.05ppm, MeOD-d_4 $\delta(^1H)$= 3.31ppm, D_2O $\delta(^1H)$= 4.79ppm, CD_2Cl_2 $\delta(^1H)$= 5.32ppm.[1] The chemical shift δ is reported in ppm and the coupling constant *J* in Hz. The multiplicity is abbreviated as following, s (singlet), d (doublet), t (triplet), hept (heptet) and m (multiplet). The assignment was realized by the measurement of two-dimensional spectra (^1H -^1H -COSY, ^1H-^{13}C-HMBC, ^1H -^{13}C-HSQC).

Mass spectrometric measurements were performed on Bruker maXis 4G mass spectrometer and conducted by the analytical division of the faculty for chemistry at the University of Basel. Molecular ions are abbreviated as *M* and the *m/z* value is given in comparison to the calculated *m/z* value.

Infrared spectra were measured on a BRUKER ALPHA IR spectrometer (ATR, attenuated total reflection). The intensity was described as follows: s (strong), m (medium) and w (weak).

Solvents: Anhydrous tetrahydrofuran (THF), anhydrous diethyl ether (Et_2O) and dichloromethane (DCM) and anhydrous *N,N*-dimethylformamide (DMF) were purchased from ACROS ORGANICS.

For work-up, column chromatography, and reactions under non- anhydrous conditions cyclohexane (*c*-hex), ethyl acetate (EtOAc), tetrahydrofuran (THF), dichloromethane (DCM), methanol (MeOH), toluene (Tol) and isopropanol (*i*-PrOH) were purchased from VWR and Honeywell in HPLC-grade quality.

Solvents for NMR spectroscopy were purchased from CAMBRIDGE ISOTOPE LABORATORIES [$CDCl_3$ (99.8%), DMSO- d_6 (99.9%)] or ACROS ORGANICS [acetone- d_6 (99.8%), CD_2Cl_2 (99.8%), MeOD-d_4 (99.8%), D_2O (99.9%)].

Chemicals: All reagents used were purchased from commercial distributers and used without further purification unless otherwise stated.

2. Synthesis

Methyl 4-methoxy-2-((3-methoxyphenyl)amino)benzoate (5)

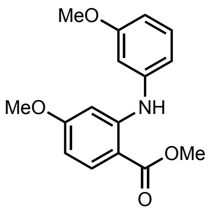

5

$C_{16}H_{17}NO_4$
M= 287.32

2-Bromo-4-methoxybenzoic acid (10.0 g, 43.3 mmol, 1.00 equiv.) was dissolved in methanol (86 mL). Sulfuric acid (conc. 19.4 mL, 346 mmol, 8.00 equiv.) was added at 0 °C and the reaction mixture was refluxed for 19 h. After the reaction mixture cooled down to r.t., the solvent was removed *in vacuo* and the residue was extracted with DCM (3x 150 mL). The organic layer was washed with $NaHCO_3$ (2x 100 mL), water (1x 100 mL) and brine (1x 100 mL). The organic layer was dried over $MgSO_4$, filtered, and the solvent was removed in vacuo. 2-Bromo-4-methoxybenzoate was obtained as a yellow oil and used without further purification.

The crude 2-bromo-4-methoxybenzoate, caesium carbonate (21.2 g, 64.9 mmol, 1.50 equiv.), palladium(II)acetate (468 mg, 2.08 mmol, 0.05 equiv.) and rac-BINAP (2.16 g, 3.46 mmol, 0.08 equiv.) were dissolved in anhydrous 1,4-dioxane (173 mL) in a dried flask under an argon atmosphere. The reaction mixture was degassed with argon, 3-methoxyaniline (7.26 mL, 64.9 mmol, 1.50 equiv.) was added, and the reaction mixture was refluxed for 48 h. After the reaction mixture cooled down to r.t., water (150 mL) and EtOAc (100 mL) were added. The aqueous layer was extracted with EtOAc (2x 200 mL). The combined organic layer was washed with brine (1x 200 mL), dried over $MgSO_4$, filtered, and the solvent was removed in vacuo. The crude product was purified *via* column chromatography (120 g silica, *c*-hex:DCM = 9:1 to 1:1). Compound **4** (11.7 g, 40.7 mmol, 94%) was obtained as a brown oil.

The spectroscopic data is congruent to literature reported data.[2]

^{1}H NMR (500 MHz, $CDCl_3$, 298K) δ 7.91 (d, J = 9.0 Hz, 1H), 7.24 (d, J = 8.1 Hz, 1H), 6.87 (d_d, J = 7.9 Hz, J = 2.0 Hz, 1H), 6.81 (t, J = 2.2 Hz, 1H), 6.77 (d, J = 2.5 Hz, 1H), 6.65 (d_d, J = 8.4, J = 2.5 Hz, 1H), 6.31 (d_d, J = 8.9 Hz, J = 2.5 Hz, 1H), 3.87 (s, 3H) 3.80 (s, 3H), 3.75 (s, 3H).

^{13}C NMR (126 MHz, $CDCl_3$, 298K) δ 168.8, 164.5, 160.8, 149.8, 142.0, 133.6, 130.2, 115.0, 109.5, 108.2, 105.5, 104.9, 98.0, 55.4, 55.4, 51.7.

6a: Synthesis was described in our initial publication.[2]

3,6-Dimethoxy-9,9-diethyl-9,10-dihydroacridine (6b)

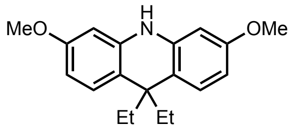

$C_{19}H_{23}NO_2$
M= 297.40

6b

5 (1.00 g, 3.48 mmol, 1.00 equiv.) was dissolved in anhydrous THF/Et_2O (1:1, 21 mL) under an argon atmosphere. Ethylmagnesium bromide (3M in THF, 5.81 mL, 17.4 mmol, 5.00 equiv.) was added at 0 °C and stirred at 0 °C for 10 min. After removal of the ice bath, the reaction mixture was stirred at r.t. for 96 h. Saturated aq. NH_4Cl solution (20 mL) and water (10 mL) were added at 0 °C. Next, the solvent was removed *in vacuo*. DCM was added, and the aqueous layer was extracted with DCM (3x 45 mL). The combined organic layer was washed with brine (1x 150 mL) and dried over $MgSO_4$. The solvent was removed *in vacuo*. 3-(4-methoxy-2-((3-methoxyphenyl)amino)phenyl)pentan-3-ol was obtained as an orange oil and was used without further purification.

The crude methoxy-2-((3-methoxyphenyl)amino)phenyl)pentan-3-ol was dissolved in anhydrous DCM (40 mL) under an argon atmosphere. Boron trifluoride diethyl etherate (2.07 mL, 8.70 mmol, 2.5 equiv.) was added, and the reaction mixture was stirred at 0 °C for 1 h and at r.t. for 1 h. Saturated aq. $NaHCO_3$ solution (15 mL) and water (20 mL) were added, and the organic layer was separated. The aqueous layer was extracted with DCM (3x 50 mL), and the combined organic layer were dried over $MgSO_4$, filtered, and the solvent was removed *in vacuo*. The crude product was purified *via* column chromatography (40 g silica, *c*-hex:DCM = 9:1 to 1:1). Compound **6b** (755 mg, 2.54 mmol, 73%) was obtained as light brown solid.

$\boldsymbol{R_f}$= 0.13 (*c*-hex/DCM 1:1).

^{1}H NMR (500 MHz, Acetone-d_6, 298K) δ 7.82 (s, 1H), 7.11 (d, J = 8.6 Hz, 2H), 6.40 (d_d, J = 8.6 Hz, J = 2.6 Hz, 2H), 6.27 (d, J = 2.6 Hz, 2H), 3.73 (s, 6H), 1.87 (q, J = 7.3 Hz, 4H), 0.56 (t, J = 7.3 Hz, 6H).

^{13}C NMR (126 MHz, Acetone-d_6, 298K) δ 159.5, 142.3, 128.2, 117.2, 106.7, 98.7, 55.2, 45.4, 38.8, 9.9.

IR ν_{max}(thin film)/cm^{-1} 3445w, 2962m, 2919m, 1516s, 1579s, 1488s, 1451m, 1366w, 1334m, 1275m, 1252m, 1219m, 1206m, 1175s, 1096m, 1034m, 988w, 813s, 788m, 719m.

HR-MS (ESI$^+$, acetone): $[M+H]^+$: $C_{19}H_{24}NO_2^+$, calculated *m/z* 298.1802, found *m/z* 298.1795.

3,6-Dimethoxy-9,9-dipropyl-9,10-dihydroacridine (6c)

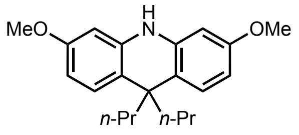

$C_{21}H_{27}NO_2$
M= 325.45

6c

5 (610 mg, 2.12 mmol, 1.00 equiv.) was dissolved in anhydrous THF/Et_2O (1:1, 13 mL) under an argon atmosphere. *n*-Propylmagnesium bromide (1M in Et_2O, 10.6 mL, 10.6 mmol, 5.00 equiv.) was added at 0 °C and stirred at 0 °C for 10 min. After removal of the ice bath, the reaction mixture was stirred at r.t. for 96 h. Saturated aq. NH_4Cl solution (20 mL) and water (20 mL) were added at 0 °C. Next, the solvent was removed *in vacuo*. DCM was added, and the aqueous layer was extracted with DCM (3x 50 mL). The combined organic layer was washed with brine (1x 200 mL) and dried over $MgSO_4$. The solvent was removed *in vacuo*. 4-(4-methoxy-2-((3-methoxyphenyl)amino)phenyl)heptan-4-ol was obtained as an orange oil and was used without further purification.

The crude 4-(4-methoxy-2-((3-methoxyphenyl)amino)phenyl)heptan-4-ol was dissolved in anhydrous DCM (13 mL) under an argon atmosphere. Boron trifluoride diethyl etherate (655 µl, 5.31 mmol, 2.50 equiv.) was added, and the reaction mixture was stirred at 0 °C for 1 h and at r.t. for 1 h. Saturated aq. $NaHCO_3$ solution (15 mL) and water (10 mL) were added, and the organic layer was separated. The aqueous layer was extracted with DCM (3x 40 mL) and the combined organic layer was dried over $MgSO_4$, filtered, and the solvent was removed *in vacuo*. The crude product was purified *via* column chromatography (24 g silica, *c*-hex:DCM = 9:1 to 4:6). Compound **6c** (573 mg, 1.76 mmol, 83%) was obtained as off-white solid.

$\boldsymbol{R_f}$= 0.15 (*c*-hex/DCM 1:1).

^{1}H NMR (500 MHz, Acetone-*d$_6$*, 298K) δ 7.81 (s, 1H), 7.14 (d, *J* = 8.4 Hz, 2H), 6.39 (d$_d$, *J* = 8.6 Hz, *J* = 2.6 Hz, 2H), 6.25 (d, *J* = 2.6 Hz, 2H), 3.27 (s, 6H), 1.86–1.79 (m, 4H), 1.03 – 0.92 (m, 4H), 0.72 (t, *J* = 7.4 Hz, 6H).

^{13}C NMR (126 MHz, Acetone-*d$_6$*, 298K) δ 159.5, 141.8, 128.1, 118.3, 106.7, 98.8, 55.2, 49.3, 44.2, 19.1, 14.8.

IR ν_{max}(thin film)/cm^{-1} 3412w, 2952w, 1616m, 1599m, 1485s, 1464s, 1327m, 1279s, 1206m, 1188m, 1168s, 1128m, 1036s, 926 w, 831s, 784m, 742w, 717w, 635m.

HR-MS (ESI$^+$, acetone): $[M+H]^+$: $C_{21}H_{28}NO_2^+$, calculated *m/z* 326.2115, found *m/z* 326.2112.

9,9-Diisobutyl-3,6-dimethoxy-9,10-dihydroacridine (6d)

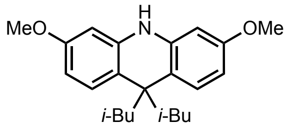

$C_{23}H_{31}NO_2$
M= 353.51

6d

5 (1.00 g, 3.48 mmol, 1.00 equiv.) was dissolved in anhydrous THF/Et_2O (1:1, 20 mL) under an argon atmosphere. Isobutyl magnesium bromide (2M in Et_2O, 8.70 mL, 17.4 mmol, 5.00 equiv.) was added at 0 °C and stirred at 0 °C for 10 min. After removal of the ice bath, the reaction mixture was stirred at r.t. for 96 h. Saturated NH_4Cl solution (20 mL) and water (20 mL) were added at 0 °C. Next, the solvent was removed *in vacuo*. DCM was added, and the aqueous layer was extracted with DCM (3x 50 mL). The combined organic layer was washed with brine (1x 150 mL) and dried over $MgSO_4$. The solvent was removed *in vacuo*. 4-(4-methoxy-2-((3-methoxyphenyl)amino)phenyl)-2,6-dimethylheptan-4-ol was obtained as a yellow-orange oil and was used without further purification.

The crude 4-(4-methoxy-2-((3-methoxyphenyl)amino)phenyl)-2,6-dimethylheptan-4-ol was dissolved in anhydrous DCM (21 mL) under an argon atmosphere. Boron trifluoride diethyl etherate (1.07 mL, 8.70 mmol, 2.50 equiv.) was added, and the reaction mixture was stirred at r.t. over night. Saturated aq. $NaHCO_3$ solution (15 mL) and water (15 mL) were added, and the organic layer was separated. The aqueous layer was extracted with DCM (3x 50 mL), and the combined organic layer was dried over $MgSO_4$, filtered, and the solvent was removed *in vacuo*. The crude product was purified *via* column chromatography (40 g silica, *c*-hex:DCM = 95:5 to 1:1). Compound **6d** (848 mg, 2.40 mmol, 69%) was obtained as pale brown solid.

$\boldsymbol{R_f}$= 0.16 (*c*-hex/DCM 1:1).

^{1}H NMR (500 MHz, Acetone-d_6, 298K) δ 7.84 (s, 1H), 7.11 (d, J = 8.6 Hz, 2H), 6.37 (d_d, J = 8.6 Hz, J = 2.6 Hz, 2H), 6.23 (d, J = 2.6 Hz, 2H), 3.27 (s, 6H), 1.80 (d, J = 5.9 Hz, 4H), 1.51-1.39 (m, 2H), 0.54 (d, J = 6.7 Hz, 12H).

^{13}C NMR (126 MHz, Acetone-d_6, 298K) δ 159.5, 141.2, 129.6, 118.5, 106.4, 98.6, 57.0, 55.1, 43.4, 26.1, 24.6.

IR ν_{max}(thin film)/cm^{-1} 3417w, 2953m, 2918m, 2864 w, 1616m, 1597m, 1487s, 1466s, 1455s, 1323m, 1278s, 1201m, 1184m, 1167s, 1126m, 1038s, 923w, 831s, 784m, 743w, 728w, 640m.

HR-MS (ESI$^+$, acetone): $[M+H]^+$: $C_{23}H_{32}NO_2^+$, calculated *m/z* 354.2428, found *m/z* 354.2424.

9,9-Dimethyl-9,10-acridane-3,6-diol (7a)

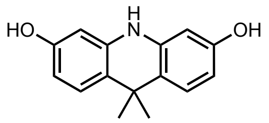

7a

6a (2.31 g, 8.57 mmol, 1.00 equiv.) was dissolved in anhydrous DCM (37 mL) in a dried flask under an argon atmosphere. Boron tribromide (1M in DCM, 30.0 mL, 30.0 mmol, 3.50 equiv.) was added at -78 °C. The reaction mixture was stirred at r.t. for 72 h. Hydrochloric acid (1M, 10 mL) was added at 0 °C and the reaction mixture was stirred for 30 min. Subsequently, saturated aq. $NaHCO_3$ (25 mL) solution was added, and the organic layer was separated. The aqueous layer was extracted with DCM (3x 50mL), and the combined organic layer was dried over $MgSO_4$, filtered, and the solvent was removed *in vacuo*. The crude product was purified *via* column chromatography (40 g silica, *c*-hex:EtOAc = 1:0 to 1:1). Compound **7a** (1.94 g, 8.06 mmol, 94%) was obtained as off-white solid.

The spectroscopic data is congruent to literature reported data.[2]

^{1}H NMR (500 MHz, Acetone-d_6, 298K) δ 7.94 (s, 2H), 7.80 (s, 1H), 7.16 (d, J = 8.5 Hz, 2H), 6.32 (dd J = 8.4 Hz, J = 2.5 Hz, 2H), 6.27 (d, J = 2.5 Hz, 2H), 1.47 (s, 6H).

^{13}C NMR (126 MHz, Acetone-d_6, 298K) δ 157.0, 140.8, 127.3, 121.6, 108.1, 100.8, 35.7, 32.2.

9,9-Diethyl-9,10-acridane-3,6-diol (7b)

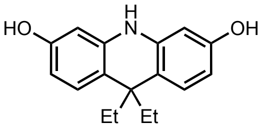

$C_{17}H_{19}NO_2$
M= 269.34

7b

6b (212 mg, 713 μmol, 1.00 equiv.) was dissolved in anhydrous DCM (2.8 mL) in a dried flask under an argon atmosphere. Boron tribromide (1M in DCM, 2.14 mL, 2.14 mmol, 3.00 equiv.) was added at -78 °C. The reaction mixture was stirred at r.t. for 72 h. Hydrochloric acid (1M, 5 mL) was added at 0 °C and the reaction mixture was stirred for 30 min. Saturated $NaHCO_3$ solution (10 mL) was added, and the organic layer was separated. The aqueous layer was extracted with DCM (3x 20 mL), and the combined organic layer was dried over $MgSO_4$, filtered, and the solvent was removed *in vacuo*. The crude product was purified *via* column chromatography (12 g silica, *c*-hex:EtOAc = 9:1 to 1:1). Compound **7b** (131 mg, 487 μmol, 68%) was obtained as pale green solid.

R_f= 0.47 (*c*-hex/EtOAc 1:1).

^{1}H NMR (500 MHz, Acetone-d_6, 298K) δ 7.92 (s, 2H), 7.65 (s, 1H), 7.01 (d, J = 8.4 Hz, 2H), 6.31 (d_d, J = 8.4 Hz, J = 2.5 Hz, 2H), 6.20 (d, J = 2.5 Hz, 2H), 1.84 (q, J = 7.3 Hz, 4H), 0.57 (t, J = 7.3 Hz, 6H).

^{13}C NMR (126 MHz, Acetone-d_6, 298K) δ 156.9, 142.4, 128.1, 116.2, 108.2, 100.2, 45.2, 38.8, 9.9.

IR ν_{max}(thin film)/cm^{-1} 3314m, 2972w, 1608s, 1480s, 1421m, 1375w, 1327m, 1292s, 1167s, 1121m, 1094m, 997s, 954w, 844s, 798m, 638m.

HR-MS (ESI$^+$, acetone): $[M+H]^+$: $C_{17}H_{20}NO_2^+$, calculated *m/z* 270.1489, found *m/z* 270.1482.

9,9-Dipropyl-9,10-acridane-3,6-diol (7c)

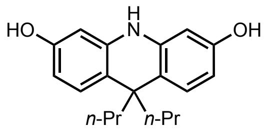

$C_{19}H_{23}NO_2$
M= 297.40

7c

6c (600 mg, 1.84 mmol, 1.00 equiv.) was dissolved in anhydrous DCM (2 mL) in a dried flask under an argon atmosphere. Boron tribromide (1M in DCM, 5.53 mL, 5.53 mmol, 3.00 equiv.) was added at -78 °C. The reaction mixture was stirred at r.t. for 72h. Hydrochloric acid (1M, 10mL) was added at 0 °C and the reaction mixture was stirred for 30 min. Saturated aq. $NaHCO_3$ solution (15 mL) was added, and the organic layer was separated. The aqueous layer was extracted with DCM (3x 40 mL), and the combined organic layer was dried over $MgSO_4$, filtered, and the solvent was removed *in vacuo*. The crude product was purified *via* column chromatography (24 g silica, *c*-hex:EtOAc = 95:5 to 6:4). Compound **7c** (334 mg, 1.12 mmol, 61%) was obtained as pale green solid.

R_f= 0.52 (*c*-hex/EtOAc 1:1).

^{1}H NMR (500 MHz, Acetone-d_6, 298K) δ 7.91 (s, 2H), 7.64 (s, 1H), 7.04 (d, *J* = 8.4 Hz, 2H), 6.30 (d_d, *J* = 8.4 Hz, *J* = 2.5 Hz, 2H), 6.18 (d, *J* = 2.5 Hz, 2H), 1.83–1.75 (m, 4H), 1.06-0.93 (m, 4H), 0.72 (t, *J* = 7.4 Hz, 6H).

^{13}C NMR (126 MHz, Acetone-d_6, 298K) δ 156.8, 141.9, 128.1, 117.3, 108.1, 100.2, 49.4, 44.1, 19.0, 14.8.

IR ν_{max}(thin film)/cm^{-1} 3389m, 2952m, 2929m, 2869w, 1611s, 1487s, 1454s, 1283m, 1232w, 1167s, 1118m, 1100w, 999s, 953w, 825s, 796w, 725w, 636m.

HR-MS (ESI$^+$, acetone): $[M+H]^+$: $C_{19}H_{24}NO_2^+$, calculated *m/z* 298.1802, found *m/z* 298.1797.

9,9-Diisobutyl-9,10-acridane-3,6-diol (7d)

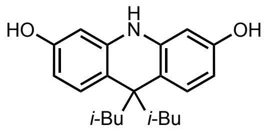

$C_{21}H_{27}NO_2$
M= 325.45

7d

6d (152 mg, 430 µmol, 1.00 equiv.) was dissolved in anhydrous DCM (1.7 mL) in a dried flask under an argon atmosphere. Boron tribromide (1M in DCM, 1.29 mL, 1.29 mmol, 3.00 equiv.) was added at -78 °C. The reaction mixture was stirred at r.t. for 72 h. Hydrochloric acid (1M, 7 mL) was added at 0 °C and the reaction mixture was stirred for 30 min. Saturated aq. $NaHCO_3$ solution (15mL) was added, and the organic layer was separated. The aqueous layer was extracted with DCM (3x 30 mL), and the combined organic layer were dried over $MgSO_4$, filtered, and the solvent was removed *in vacuo*. The crude product was purified *via* column chromatography (12g silica, *c*-hex:EtOAc = 9:1 to 1:1). Compound **7d** (36.0 mg, 112 µmol, 26%) was obtained as green solid.

***R*f**= 0.42 (*c*-hex/EtOAc 1:1).

^{1}H NMR (500 MHz, Acetone-d_6, 298K) δ 7.91 (s, 2H), 7.68 (s, 1H) 7.01 (d, *J* = 8.4 Hz, 2H), 6.28 (dd, *J* = 8.5 Hz, *J* = 2.5 Hz, 2H), 6.16 (d, *J* = 2.5 Hz, 2H), 1.77 (d, *J* = 5.8 Hz, 4H), 1.53–1.40 (m, 2H), 0.55 (d, *J* = 6.7 Hz, 12H).

^{13}C NMR (126 MHz, Acetone-d_6, 298K) δ 156.9, 141.3, 129.5, 117.5, 107.8, 100.2, 57.0, 43.3, 26.2, 24.6.

IR ν_{max}(thin film)/cm^{-1} 3400w, 2949w, 1611m, 1484s, 1449m, 1331w, 1280m, 1166s, 1105m, 1000m, 828s, 794m, 749w, 643m.

HR-MS (ESI$^+$, acetone): $[M+H]^+$: $C_{21}H_{28}NO_2^+$, calculated *m/z* 326.2115, found *m/z* 326.2114.

Ethyl-acridane[4]arene (8a)

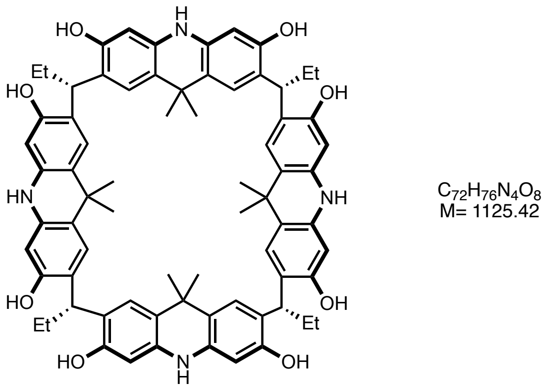

8a

7a (250 mg, 1.04 mmol, 1.00 eq) and propionaldehyde (74.8 µL, 1.04 mmol, 1.00 equiv.) were dissolved in DCM (47 mL) and the solution was degassed *via* sparging with argon for 25 min. Triflic acid (9.17 µl, 104 µmol, 0.10 equiv.) was added, and the reaction mixture was stirred at 30 °C for 7 days. Saturated aq. $NaHCO_3$ solution (5 mL) and water (50 mL) were added, the layers were separated, and the aqueous layer was extracted with DCM:*i*PrOH (3:1, 3x 70 mL). The combined organic layer was dried over $MgSO_4$, filtered, and the solvent was removed *in vacuo*. The crude product was refluxed with EtOAc, the solid was collected and dried under high vacuum. Compound **8a** (152 mg, 135 µmol, 52 %) was obtained as green solid.

^{1}H NMR (500 MHz, Acetone-d_6, 298K) δ 8.18 (s, 8H), 7.55 (s, 4H), 7.39 (s, 8H), 6.12 (s, 8H), 4.28 (t, J = 7.9 Hz, 4H), 2.18 (p, J = 7.3 Hz, 8H), 1.70 (s, 12H), 1.41 (s, 12H), 0.91 (t, J = 7.2 Hz, 12H).

^{13}C NMR (126 MHz, Acetone-d_6, 298K) δ 153.0, 137.6, 125.4, 124.3, 122.3, 100.6, 69.7, 55.5, 32.0, 30.6, 27.5, 13.1.

IR ν_{max}(thin film)/cm^{-1} 3396w, 3368w, 2959m, 2925w, 2869w, 1617s, 1492s, 1460m, 1421m, 1383m, 1280m, 1216m, 1168m, 1124s, 1062s, 887m, 827s, 760w.

HR-MS (ESI$^-$, acetone): [M-H]$^-$: $C_{72}H_{75}N_4O_8^-$, calculated *m/z* 1123.5590, found *m/z* 1123.5595.

4-Chlorobutyl-acridane[4]arene (8b)

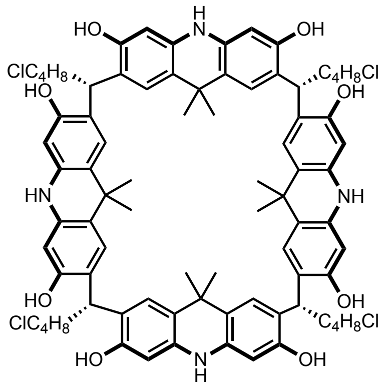

$C_{80}H_{88}Cl_4N_4O_8$
M= 1375.40

8b

7a (300 mg, 1.24 mmol, 1.00 equiv.) and 5-chloropentanal (0.15 mL, 1.24 mmol, 1.00 equiv.) were dissolved in DCM (56 mL) and the solution was degassed *via* sparging with argon for 20 min. Triflic acid (11.0 µl, 124 µmol, 0.10 equiv.) was added, and the reaction mixture was stirred at 30 °C for 7 days. Saturated aq. $NaHCO_3$ solution (10 mL) and water (60 mL) were added, the layers were separated, and the aqueous layer was extracted with DCM:*i*PrOH (3:1, 3x 80 mL). The combined organic layer was dried over $MgSO_4$, filtered, and the solvent was removed *in vacuo*. The crude product was recrystallized from dioxane, the solid was collected and dried under high vacuum. Compound **8b** (282 mg, 205 µmol, 66 %) was obtained as off-white solid.

^{1}H NMR (500 MHz, Acetone-d_6, 298K) δ 8.18 (s, 8H), 7.57 (s, 4H), 7.42 (s, 8H), 6.14 (s, 8H), 4.42 (t, *J* = 7.9 Hz, 4H), 3.60 (t, *J* = 6.8 Hz, 8H), 2.21 (q, *J* = 7.8 Hz, 8H), 1.84 (p, *J* = 7.0 Hz, 8H), 1.71 (s, 12H), 1.50 – 1.41 (m, 8H), 1.40 (s, 12H).

^{13}C NMR (126 MHz, Acetone-d_6, 298K) δ 153.0, 137.7, 125.5, 124.2, 122.3, 100.7, 45.8, 36.0, 34.3, 34.1, 33.8, 33.6, 33.4, 26.2.

IR ν_{max}(thin film)/cm^{-1} 3363w, 2951w, 2858w, 1617s, 1491s, 1426m, 1277m, 1216m, 1173m, 1121w, 870w, 830m, 752w.

HR-MS (ESI$^-$, acetone): [M-H]$^-$: $C_{80}H_{87}Cl_4N_4O_8^-$, calculated *m/z* 1371.5284, found *m/z* 1371.5288.

8c-d: Synthesis was described in our initial publication.[2]

Diethyl-acridane[4]arene (8e)

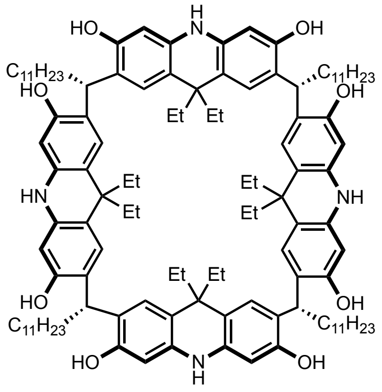

$C_{116}H_{164}N_4O_8$
M= 1742.61

8e

7b (131 mg, 489 µmol, 1.00 equiv.) and dodecanal (109 µL, 489 µmol, 1.00 equiv.) were dissolved in DCM (22 mL) and the solution was degassed *via* sparging with argon for 10 min. Triflic acid (4.32 µl, 48.9 µmol, 0.10 equiv.) was added, and the reaction mixture was stirred at 30 °C for 7 days. Saturated aq. $NaHCO_3$ solution (8 mL) and water (30 mL) were added, the layers were separated and the aqueous layer was extracted with DCM:*i*PrOH (3:1, 3x 40 mL). The combined organic layer was dried over $MgSO_4$, filtered, and the solvent was removed *in vacuo*. The crude compound **8e** (213mg, 122 µmol) was obtained brown solid.

^{1}H NMR (500 MHz, Acetone-d_6, 273K) δ 8.23 (s, 8H), 7.45 (s, 4H), 7.13 (s, 8H), 6.11 (s, 8H), 4.36 (t, J = 7.7 Hz, 4H), 2.13 (q, J = 7.8 Hz 8H), 1.95 – 1.89 (m, 8H), 1.29 (m, 80H), 0.93 – 0.82 (m, 12H), 0.66 (t, J = 7.2 Hz, 12H), 0.25 (t, J = 7.2 Hz, 12H).

^{13}C NMR (126 MHz, Acetone-d_6, 273K) δ 152.7, 139.9, 125.1, 124.5, 117.2, 100.3, 45.8, 39.9, 39.5, 34.5, 34.0, 32.7, 30.4, 30.4, 30.3, 30.2, 30.1, 28.9, 23.4, 14.4, 14.3, 10.5, 10.1.

HR-MS (ESI$^-$, acetone): [M-H]$^-$: $C_{116}H_{163}N_4O_8^-$, calculated *m/z* 1740.2476, found *m/z* 1740.2470.

Di-*n*-propyl-acridane[4]arene (8f)

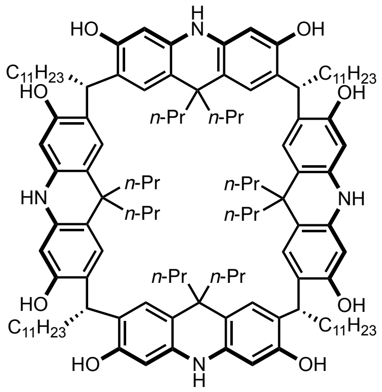

8f

7c (290 mg, 975 µmol, 1.00 equiv.) and dodecanal (0.21 mL, 975 µmol, 1.00 equiv.) were dissolved in DCM (45 mL) and the solution was degassed *via* sparging with argon for 15 min. Triflic acid (8.63 µl, 97.5 µmol, 0.10 equiv.) was added, and the reaction mixture was stirred at 30 °C for 7 days. Saturated aq. $NaHCO_3$ solution (10 mL) and water (50 mL) were added, the layers were separated and the aqueous layer was extracted with DCM:*i*PrOH (3:1, 3x 50 mL). The combined organic layer was dried over $MgSO_4$, filtered, and the solvent was removed *in vacuo*. The crude compound **8f** (456mg, 244 µmol) was obtained brown solid.

^{1}H NMR (500 MHz, Acetone-d_6, 273K) δ 8.00 (s, 8H), 7.46 (s, 4H), 7.13 (s, 8H), 6.15 (s, 8H), 4.41 (t, *J* = 7.7 Hz, 4H), 1.84 (d_{dd}, *J* = 18.2, 8.2, 4.4 Hz, 16H), 1.29 (s, 96H), 1.13 (q_d, *J* = 9.7, 5.9 Hz, 8H), 0.95 (d_q, *J* = 11.6, 6.1 Hz, 8H), 0.89 (t, *J* = 6.8 Hz, 12H), 0.84 (t, *J* = 7.3 Hz, 12H), 0.60 (t, *J* = 7.4 Hz, 12H).

^{13}C NMR (126 MHz, Acetone-d_6, 273K) δ 153.2, 139.7, 125.4, 123.8, 117.7, 100.3, 49.8, 47.9, 44.3, 35.6, 34.7, 32.7, 30.4, 30.3, 30.1, 28.9, 23.3, 19.0, 18.9, 15.9, 15.2, 14.4.

HR-MS (ESI$^-$, acetone): $[M-H]^-$: $C_{124}H_{179}N_4O_8^-$, calculated *m/z* 1852.3728, found *m/z* 1852.3754.

***N,O-Boc*-undecyl-acridane[4]arene (9)**

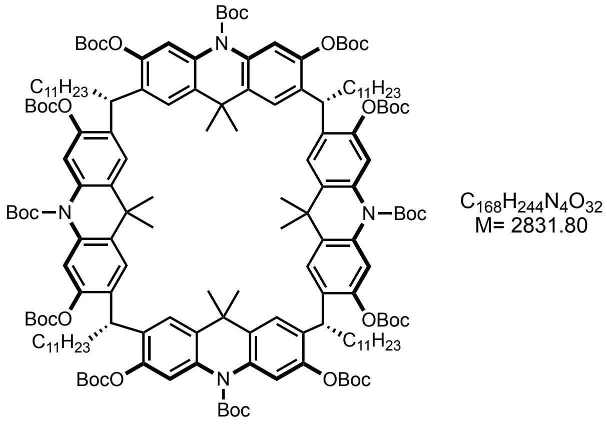

9

A suspension of **8c** (250 mg, 153 μmol, 1.00 equiv.) in anhydrous DCM (15 mL) was degassed *via* sparging with argon for 20 min, treated with DMAP (7.48 mg, 61.2 μmol, 0.40 equiv.) and Boc_2O (1.4 mL, 6.12 mmol, 40.0 equiv.) and stirred for 4 d at room temperature. Subsequently water (100 mL) was added, and the aqueous layer was extracted with DCM (3 × 100 mL). The combined organic layers were washed with water (200 mL), dried over $MgSO_4$, filtered, and the solvent and Boc_2O was removed *in vacuo* to obtain **9** (433 mg, 153 μmol, quant.) as a grey solid.

^{1}H NMR (500 MHz, Acetone-d_6, 298K) δ 7.48 (s, 8H), 7.28 (s, 8H), 4.56 (t, *J* = 7.7 Hz, 4H), 2.03 – 1.95 (m, 8H), 1.52 (s, 63H), 1.50 (s, 36H), 1.32 – 1.21 (m, 105H), 0.90 – 0.81 (m, 12H).

^{13}C NMR (126 MHz, Acetone-d_6, 298K) δ 152.5, 148.0, 138.7, 137.9, 132.8, 123.9, 120.6, 83.6, 82.8, 38.3, 32.6, 30.4, 30.3, 30.2, 30.1, 30.1, 30.0, 29.9, 29.9, 29.8, 29.8, 28.7, 28.4, 28.0, 27.8, 23.3, 14.4.

IR ν_{max}(thin film)/cm^{-1} 2925.4m, 2854.4m, 1757.4s, 1717.0m, 1482.9m, 1394.2w, 1368.9m, 1319.4m, 1246.7s, 1154.1s, 1126.1s, 1047.9w, 914.8w, 896.6w, 849.7w, 814.4w, 779.7m, 461.6w.

HR-MS (ESI$^+$, acetone): [M+Na]$^+$: $C_{168}H_{244}N_4O_{32}$+Na$^+$, calculated *m/z* 2852.7481, found *m/z* 2852.7537.

N-Boc-undecyl-acridane[4]arene (10)

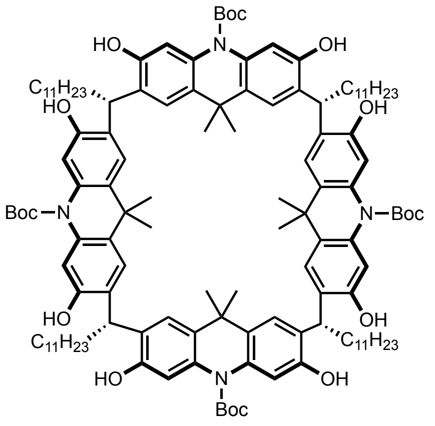

$C_{128}H_{180}N_4O_{16}$
M= 2030.86

10

A solution of **12** (189 mg, 66.7 µmol, 1.0 equiv.) in anhydrous MeOH/THF (1:1, 7.5 mL) was cooled to 0 °C, treated with NaOMe (72.1 mg, 1.33 mmol, 20 equiv.), let come to room temperature and stirred for 2 d. Subsequently water (20 mL) and conc. AcOH (0.5 mL) were added, and the mixture was stirred for 30 min. The mixture was treated with saturated aq. $NaHCO_3$ solution (20 mL) and extracted with DCM (3× 50 mL). The combined organic layers were washed with water (100 mL), dried over $MgSO_4$, filtered, and the solvent was removed *in vacuo*. The crude product was purified *via* flash column chromatography (12 g silica gel, DCM:MeOH = 99:1 to 9:1) to obtain **10** (50.9 mg, 25.1 µmol, 38%) as a brown solid.

***R*$_f$**= 0.44 (DCM/MeOH 9:1).

^{1}H NMR (500 MHz, Acetone-*d$_6$*, 298K) δ 8.45 (s, 8H), 7.32 (s, 8H), 7.12 (s, 8H), 4.71 (t, *J* = 7.8 Hz, 4H), 2.18 – 2.13 (m, 8H), 1.42 (s, 36H), 1.34 – 1.24 (m, 96H), 0.90 – 0.87 (m, 12H).

^{13}C NMR (126 MHz, Acetone-*d$_6$*, 298K) δ 152.9, 152.6, 137.9, 133.9, 122.1, 113.6, 81.6, 37.4, 35.7, 34.3, 32.7, 30.4, 30.3, 30.2, 30.1, 30.1, 30.1, 29.9, 29.8, 28.7, 28.3, 23.4, 14.4.

IR ν_{max}(thin film)/cm^{-1} 3260w, 2923w, 2853w, 1717m, 1485w, 1367m, 1318s, 1247s, 1151s, 1090m, 854m, 763m, 644w.

HR-MS (ESI$^-$, acetone): $[M-2H]^{2-}$: $C_{128}H_{178}N_4O_{16}^{2-}$, calculated *m/z* 1013.6624, found *m/z* 1013.6634.

***O-Boc*-undecyl-acridane[4]arene (11)**

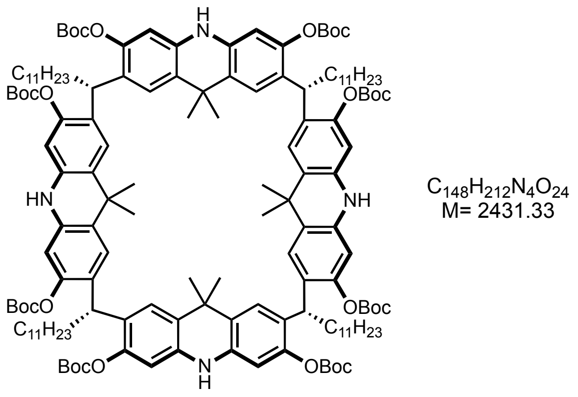

11

A suspension of **8c** (100 mg, 61.3 µmol, 1.00 equiv.) in anhydrous DCM (5 mL) was degassed *via* sparging with argon for 5 min, treated with Et_3N (0.25 mL, 1.78 mmol, 29.0 equiv.) and Boc_2O (0.30 mL, 1.29 mmol, 21.0 equiv.) and sonicated for 2 h at r.t.. Subsequently saturated aq. NH_4Cl solution (5 mL) and water (20 mL) were added, and the aqueous layer was extracted with DCM (3 × 30 mL). The combined organic layers were washed with water (100 mL), dried over $MgSO_4$, filtered, and the solvent and Boc_2O was removed *in vacuo.* The crude product was purified *via* flash column chromatography (4 g silica gel, *c*-hex:DCM = 1:0 to 0:1) to give **11** (125 mg, 51.7 µmol, 84%) as a beige solid.

$\boldsymbol{R_f}$= 0.44 (*c*-hex/DCM 1:1).

^{1}H NMR (500 MHz, Acetone-*d₆*, 298K) δ 8.07 (s, 4H), 7.31 (s, 8H), 6.53 (s, 8H), 4.36 (t, *J* = 7.7 Hz, 4H), 1.72 (s, 12H), 1.49 – 1.52 (m, 72H), 1.95 – 1.90 (m, 8H), 1.36 (s, 12H), 1.26 – 1.29 (m, 72H), 0.86 (t, *J* = 6.7 Hz, 12H).

^{13}C NMR (126 MHz, Acetone-d_6, 298K) δ 152.7, 148.8, 138.3, 128.1, 126.6, 126.3, 108.1, 83.0, 36.8, 36.7, 36.0, 33.5, 30.4, 30.3, 30.2, 30.1, 30.0, 29.9, 29.8, 28.7, 27.8, 27.4, 23.3, 14.4.

IR ν_{max}(thin film)/cm^{-1} 2922w, 2853w, 1175m, 1616w, 1492m, 1369w, 1241s, 1154m, 1122s, 1001m, 881w, 780w, 464m.

HR-MS (ESI$^-$ acetone): $[M-H]^-$: $C_{148}H_{211}N_4O_{24}^-$, calculated *m/z* 2428.5419, found *m/z* 2428.5402.

Methylene-bridged-undecyl-acridane[4]arene (12)

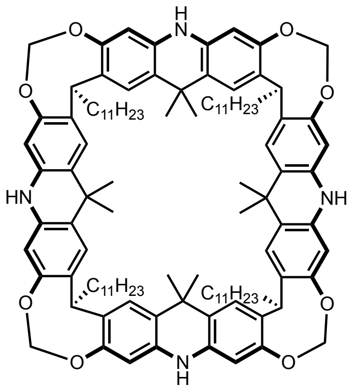

12

A solution of **8c** (200 mg, 123 µmol, 1.00 equiv.) in anhydrous DMF (12 mL) was degassed *via* sparging with argon for 25 min and subsequently treated with potassium carbonate (340 mg, 2.46 mmol, 20.0 equiv.) and dibromomethane (0.17 mL, 2.46 mmol, 20.0 equiv.). The reaction mixture was stirred at 70 °C for 24 h. The solvent was removed *in vacuo* and water (50 mL) and DCM (35 mL) were added and sonicated to give a homogenous suspension. The layers were separated, and the aqueous layer was extracted with DCM (3x 50 mL). The combined organic layer was dried over Na_2SO_4, filtered, and the solvent was removed *in vacuo*. The crude product was purified *via* column chromatography (4g silica, DCM:MeOH = 1:0 to 20:1). Compound **12** (102 mg, 60.8 µmol, 50%) was obtained as brown solid.

$\boldsymbol{R}_f$= 0.18 (DCM/MeOH 98:2).

^{1}H NMR (500 MHz, Acetone-d_6, 298K) δ 7.78 (s, 4H), 7.39 (s, 8H), 6.22 (s, 8H), 5.71 (d, J = 7.3 Hz, 4H), 4.64 (t, J = 8.1 Hz, 4H), 4.47 (d, J = 7.3 Hz, 4H), 1.62 (s, 12H), 1.45 (s, 12H), 1.55– 0.25 (m, 84H), 0.92 – 0.85 (m, 12H).

^{13}C NMR (126 MHz, Acetone-d_6, 298K) δ 156.5, 137.6, 133.5, 126.3, 123.7, 107.5, 100.8, 36.8, 36.6, 34.2, 33.1, 32.7, 30.4, 30.3, 30.3, 30.2, 30.1, 30.1, 29.9, 28.8, 23.3, 14.4.

IR ν_{max}(thin film)/cm^{-1} 3356.7w, 2922.4m, 2852.2m, 1668.1m, 1614.8m, 1489.0s, 1461.7m, 1413.4m, 1383.1w, 1289.1m, 1206.1m, 1173.5s, 1130.6m, 1045.7m, 962.8s, 899.8m, 849.9m, 742.9w, 720.4w, 694.9w, 661.0w, 609.3m, 515.7w, 461.7w.

HR-MS (ESI$^+$, acetone): $[M+2H]^{2+}$: $C_{112}H_{150}N_4O_8^{2+}$, calculated *m/z* 838.5643, found *m/z* 838.5663.

***N*-4-(trifluoromethyl)phenyl-undecyl-acridane[4]arene (13)**

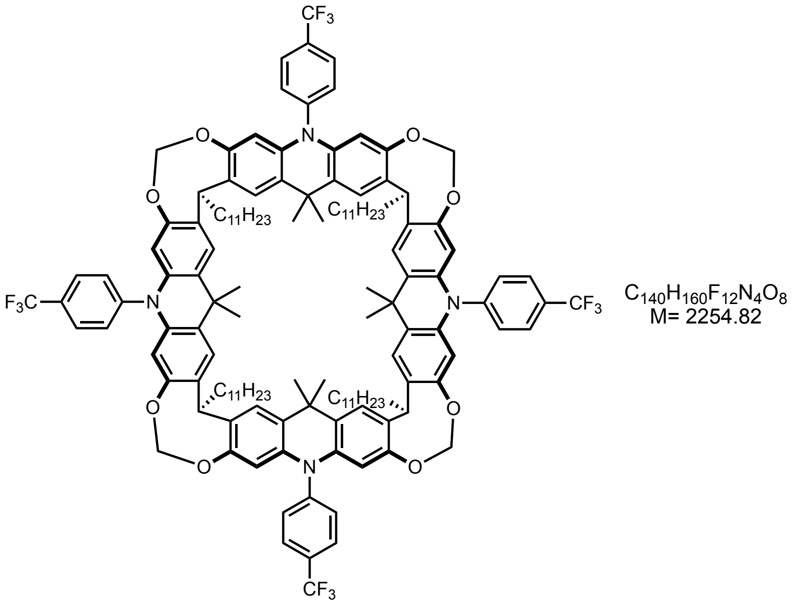

13

A solution of **12** (8.39 mg, 5.00 µmol, 1.00 equiv.) in anhydrous toluene (0.15 mL) was treated with 1-bromo-4-(trifluoromethyl)benzene (4.22 µl, 30.0 µmol, 6.00 equiv.) racemic-BINAP (1.25 mg, 2.00 µmol, 0.40 equiv.), palladium(II)acetate (0.23 mg, 1.00 µmol, 0.20 equiv.) and Cs_2CO_3 (9.77 mg, 30.0 µmol, 6.00 equiv.) and heated at 100 °C for 24 h. The reaction mixture was cooled to room temperature, treated with DCM (2 mL), and filtered through celite. The crude product was purified *via* flash column chromatography (4 g silica gel, *c*-hex:EtOAc = 1:0 to 3:1) to obtain **13** (7.50 mg, 3.33 µmol, 66%) as light yellow solid.

$\boldsymbol{R}_f$= 0.19 (*c*-hex/EtOAc 98:2).

^{1}H NMR (500 MHz, Acetone-d_6, 298K) δ 7.97 (d, *J* = 8.2 Hz, 8H), 7.58 (s, 8H), 7.50 (d, *J* = 8.0 Hz, 8H), 5.60 (s, 8H), 5.47 (d, *J* = 7.3 Hz, 5H), 4.61 (t, *J* = 8.1 Hz, 4H), 4.20 (d, *J* = 7.3 Hz, 4H), 2.33 (q, *J* = 7.9 Hz, 8H), 1.76 (s, 12H), 1.62 (s, 12H), 1.40 – 1.28 (m, 72H), 0.88 (t, *J* = 6.8 Hz, 12H).

^{13}C NMR (126 MHz, Acetone-d_6, 298K) δ 156.0, 145.5, 139.3, 134.3, 133.1, 130.9, 130.6, 129.2, 127.7, 125.9, 124.1, 123.9, 108.4, 100.7, 36.7, 36.5, 34.4, 33.4, 32.7, 30.4, 30.3, 30.3, 30.3, 28.7, 23.3, 14.4.

^{19}F NMR (470 MHz, Acetone-d_6, 293.15 K) *δ* -63.05.

IR ν_{max}(thin film)/cm^{-1} 2923.9m, 2853.3m, 1607.4m, 1485.9s, 1459.5m, 1427.5m, 1409.4w, 1321.9s, 1198.2m, 1166.1s, 1125.7s, 1101.5m, 1065.4m, 1048.8m, 1018.6m, 966.5s, 890.4w, 853.6m, 763.0m, 720.8w, 625.5m, 511.4w, 451.4w.

HR-MS (ESI$^+$, acetone): $[M+Na]^+$: $C_{140}H_{160}F_{12}N_4O_{24}+Na^+$, calculated *m/z* 2276.1937, found *m/z* 2276.1903.

N-4-benzaldehyde-undecyl-acridane[4]arene (14)

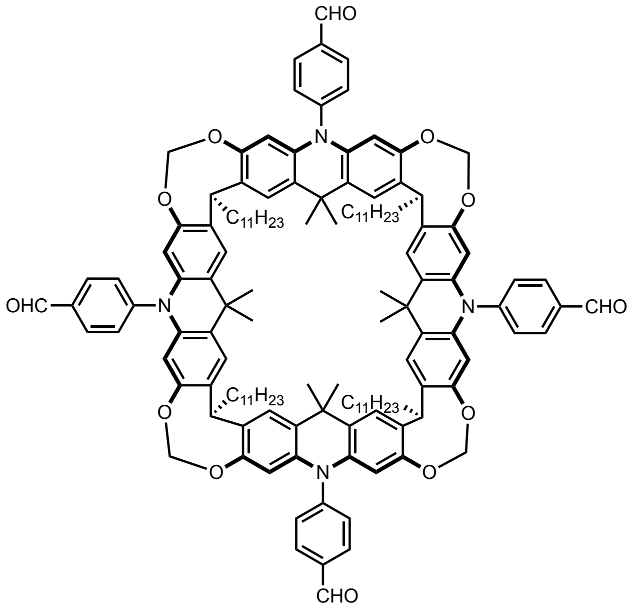

$C_{140}H_{164}N_4O_{12}$
M= 2094.87

14

A solution of **12** (17.0 mg, 10.1 µmol, 1.00 equiv.) in anhydrous toluene (0.10 mL) was treated with 4-Bromobenzaldehyde (11.2 mg, 60.6 µmol, 6.00 equiv.) racemic-BINAP (2.52 mg, 4.04 µmol, 0.40 equiv.), palladium(II)acetate (0.45 mg, 2.02 µmol, 0.20 equiv.) and Cs_2CO_3 (19.8 mg, 60.6 µmol, 6.00 equiv.) and heated at 100 °C for 48h. The reaction mixture was cooled to room temperature, treated with DCM (2 mL), and filtered through celite. The crude product was purified *via* flash column chromatography (4 g silica gel, *c*-hex:EtOAc = 1:0 to 2:1) to obtain **14** (13.0 mg, 6.21 µmol, 61%) as yellow solid.

$\boldsymbol{R_f}$= 0.20 (*c*-hex/EtOAc 7:3).

^{1}H NMR (500 MHz, Acetone-*d₆*, 298K) δ 10.14 (s, 4H), 8.18 – 8.12 (m, 8H), 7.57 (s, 8H), 7.51 – 7.44 (m, 8H), 5.63 (s, 8H), 5.46 (d, *J* = 7.3 Hz, 4H), 4.61 (t, *J* = 8.1 Hz, 4H), 4.21 (d, *J* = 7.3 Hz, 4H), 2.33 (q, *J* = 7.9 Hz, 8H), 1.77 (s, 12H), 1.62 (s, 12H), 1.40 – 1.26 (m, 72H), 0.92 – 0.84 (m, 12H).

^{13}C NMR (126 MHz, Acetone-d_6, 298K) δ 192.2, 156.0, 147.2, 139.3, 137.4, 134.3, 133.0, 132.8, 127.7, 123.9, 108.5, 100.7, 36.7, 36.6, 34.4, 33.4, 32.7, 30.4, 30.3, 30.3, 30.2, 30.1, 30.1, 29.9, 28.7, 23.3, 14.4.

IR ν_{max}(thin film)/cm^{-1} 2922.9m, 2852.2m, 1705.1m, 1596.1m, 1483.2s, 1426.7m, 1298.9m, 1196.6s, 1047.5m, 967.5s, 822.0m, 757.4w, 621.7w, 511.7w.

HR-MS (ESI$^+$, acetone): [M+Na]$^+$: $C_{140}H_{164}N_4O_{12}$+Na$^+$ calculated 2116.2238 *m/z*, found *m/z* 2116.2214.

Methylene-bridged-ethyl-acridane[4]arene (15)

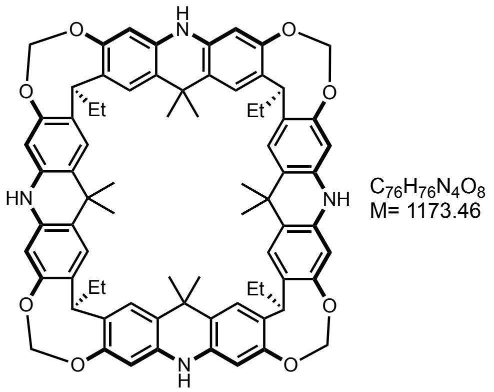

15

A solution of **8a** (152 mg, 135 μmol, 1.00 equiv.) in anhydrous DMF (13.5 mL) was degassed *via* sparging with argon for 30 min and subsequently treated with potassium carbonate (749 mg, 5.42 mmol, 40.0 equiv.) and dibromomethane (381 μl, 5.42 mmol, 40.0 equiv.). The reaction mixture was stirred at 70 °C for 72 h. The solvent was removed *in vacuo* and water (30 mL) and DCM (30 mL) were added and sonicated to give a homogenous suspension. The layers were separated, and the aqueous layer was extracted with DCM (3x 30 mL). The combined organic layer was dried over $MgSO_4$, filtered, and the solvent was removed *in vacuo*. The crude product was purified *via* column chromatography (4g silica, *c*-hex:EtOAc = 1:1 to 1:2). Compound **15** (79 mg, 67.3 μmol, 50%) was obtained as light yellow solid.

R_f= 0.14 (*c*-hex/EtOAc 1:2).

^{1}H NMR (500 MHz, Acetone-d_6, 298K) δ 7.77 (s, 4H), 7.37 (s, 8H), 6.22 (s, 8H), 5.71 (d, J = 7.3 Hz, 4H), 4.52 (t, J = 8.2 Hz, 4H), 4.47 (d, J = 7.3 Hz, 4H), 2.28 (p, J = 7.4 Hz, 8H), 1.61 (s, 12H), 1.45 (s, 12H), 0.97 (t, J = 7.2 Hz, 12H).

^{13}C NMR (126 MHz, Acetone-d_6, 298K) δ 156.6, 137.6, 133.3, 126.4, 123.7, 107.5, 100.8, 38.9, 36.6, 34.1, 33.1, 23.3, 13.1.

IR ν_{max}(thin film)/cm^{-1} 2961.3m, 1614.0m, 1488.5s, 1413.5m, 1359.6w, 1289.2m, 1208.5m, 1172.0s, 1130.3m, 1066.4w, 1044.9m, 960.9s, 901.1m, 848.5m, 745.0w, 695.6w, 610.0m, 462.0m.

HR-MS (ESI$^-$, acetone): [M-H]$^-$: $C_{76}H_{75}Cl_4N_4O_8^-$, calculated *m/z* 1173.5736, found *m/z* 1173.5718.

***N*-5-dimethyl-isophthalate-Ethyl-acridane[4]arene (16)**

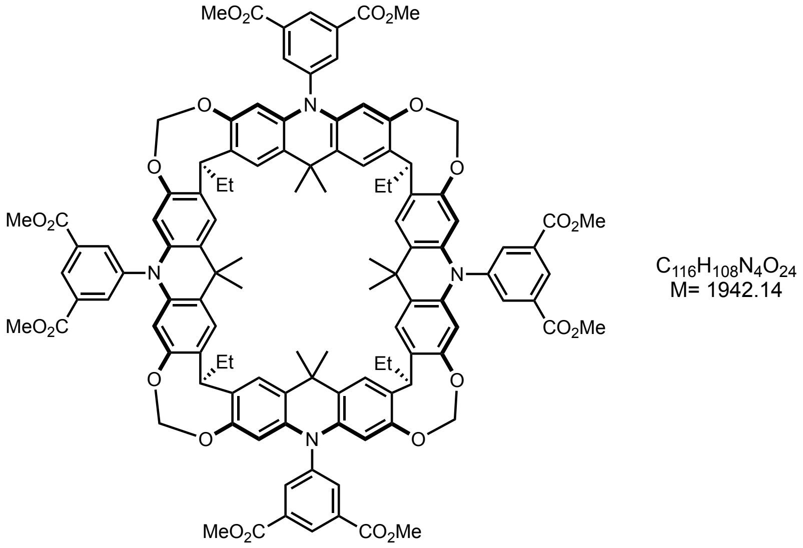

16

A solution of **15** (20.3 mg, 17.3 µmol, 1.00 equiv.) in anhydrous toluene (0.17 mL) was treated with dimethyl-5-bromisophthalat (28.3 mg, 104 µmol, 6.00 equiv.), racemic-BINAP (4.31 mg, 6.92 µmol, 0.40 equiv.), palladium(II)acetate (0.78 mg, 3.46 µmol, 0.20 equiv.) and Cs_2CO_3 (33.8 mg, 104 µmol, 6.00 equiv.) and heated at 100 °C for 48 h. The reaction mixture was cooled to room temperature, treated with DCM (5 mL), and filtered through celite. The crude product was purified *via* flash column chromatography (4 g silica gel, *c*-hex:EtOAc = 1:0 to 2:1) to obtain **16** (13.0 mg, 6.69 µmol, 39%) as light yellow solid.

$\boldsymbol{R_f}$= 0.18 (*c*-hex/EtOAc 2.3:1).

^{1}H NMR (500 MHz, Acetone-*d*$_6$, 298K) δ 8.70 (t, *J* = 1.6 Hz, 4H), 7.99 (d, *J* = 1.6 Hz, 8H), 7.31 (s, 8H), 5.50 (s, 8H), 5.38 (d, *J* = 7.3 Hz, 4H), 4.48 (t, *J* = 8.1 Hz, 4H), 4.27 (d, *J* = 7.3 Hz, 4H), 3.86 (s, 24H), 2.26 (p, *J* = 7.3 Hz, 8H), 1.76 (s, 12H), 1.57 (s, 12H), 1.01 (t, *J* = 7.2 Hz, 12H).

^{13}C NMR (126 MHz, Acetone-*d*$_6$, 298K) δ 165.5, 155.4, 141.8, 138.5, 137.3, 134.6, 133.4, 127.0, 123.5, 108.2, 100.2, 38.1, 35.9, 35.6, 34.2, 30.1, 23.0, 12.7.

IR ν_{max}(thin film)/cm^{-1} 2919m, 2850w, 1728s, 1594w, 1487s, 1459m, 1428m, 1344m, 1241s, 1212m, 1177m, 1076w, 1052w, 1019m, 1000m, 963s, 749m 722m.

HR-MS (ESI$^+$, acetonitrile): [M+Na]$^+$: $C_{116}H_{108}N_4O_{24}$+Na$^+$, calculated *m/z* 1963.7246, found *m/z* 1963.7249.

Methylene-bridged-4-chlorobutyl-acridane[4]arene (17)

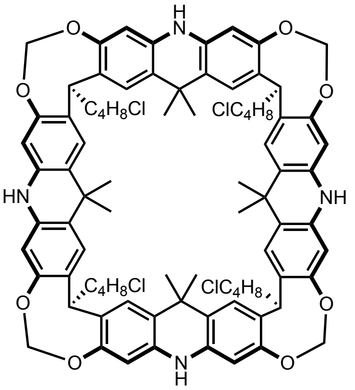

17

A solution of **8b** (65 mg, 47.3 µmol, 1.00 equiv.) in anhydrous DMF (4.7 mL) was degassed *via* sparging with argon for 15 min and subsequently treated with potassium carbonate (131 mg, 945 µmol, 20.0 equiv.) and dibromomethane (133 µl, 1.89 mmol, 40.0 equiv.) at 0°C. The reaction mixture was stirred at 30 °C for 72 h. The solvent was removed *in vacuo* and water (30 mL), and DCM (30 mL) were added and sonicated to give a homogenous suspension. The layers were separated, and the aqueous layer was extracted with DCM (3x 30 mL). The combined organic layer was dried over $MgSO_4$, filtered, and the solvent was removed *in vacuo*. The crude product was purified *via* column chromatography (4g silica, *c*-hex:EtOAc = 1:1 to 1:3). Compound **17** (33 mg, 23.2 µmol, 49%) was obtained as brown solid.

$\boldsymbol{R_f}$= 0.28 (*c*-hex/EtOAc 1:2).

^{1}H NMR (500 MHz, Acetone-*d*$_6$, 298K) δ 7.84 (s, 4H), 7.40 (s, 8H), 6.23 (s, 8H), 5.71 (d, *J* = 7.3 Hz, 4H), 4.63 (t, *J* = 8.2 Hz, 4H), 4.46 (d, *J* = 7.3 Hz, 4H), 3.64 (t, *J* = 6.7 Hz, 8H), 2.31 (q, *J* = 8.1 Hz, 8H), 1.89 (d_t, *J* = 14.3, *J* = 6.7 Hz, 8H), 1.61 (s, 12H), 1.56 – 1.46 (m, 8H), 1.44 (s, 12H).

^{13}C NMR (126 MHz, Acetone-*d*$_6$, 298K) δ 156.5, 137.7, 133.2, 126.4, 123.7, 107.5, 45.7, 36.6, 33.5, 30.4, 30.2, 30.1, 29.9, 29.8, 26.2.

IR ν_{max}(thin film)/cm^{-1} 2926.1w, 1722.4w, 1613.3m, 1489.0s, 1359.6w, 1288.7m, 1244.8m, 1172.6s, 1126.4w, 1042.2m, 961.0s, 900.8m, 848.1m, 740w, 610.5m, 465.5w.

HR-MS (ESI$^-$, acetone): [M-H$^+$]$^-$: $C_{84}H_{87}Cl_4N_4O_8^-$, calculated *m/z* 1419.5284, found *m/z* 1419.5272.

4-Pyridinium-butyl-acridane[4]arene (18)

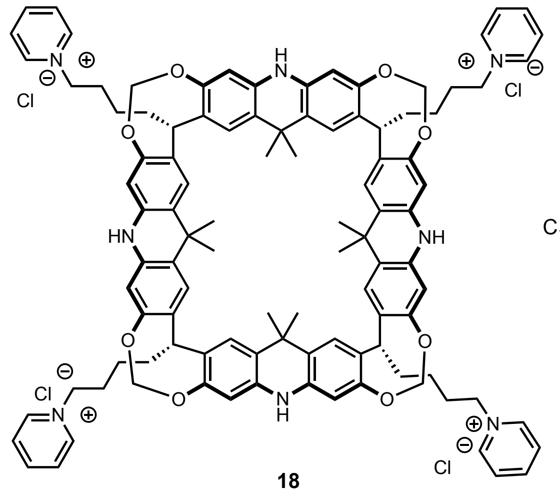

18

$C_{104}H_{107}N_8O_8Cl_4$
M= 1597.05

A solution of **17** (32.2 mg, 22.6 μmol, 1.00 equiv.) in pyridine (500 μl) under an argon atmosphere was stirred at 100 °C for 24h. The reaction mixture was cooled to 0 °C, the precipitate was filtered and suspended in acetone (10 mL). The mixture was refluxed for 2h, the solid was filtered, washed with acetone and dried under high vacuum to afford **18** (9.00 mg, 5.63 μmol, 25%).

^{1}H NMR (500 MHz, Methanol-d_4, 298K) δ 9.02 – 8.97 (m, 8H), 8.55 (t_t, J = 7.8, J = 1.4 Hz, 4H), 8.12 – 8.05 (m, 12H), 7.19 (s, 8H), 6.15 (s, 8H), 5.73 (d, J = 7.2 Hz, 4H), 4.67 (t, J = 7.3 Hz, 8H), 4.62 (t, J = 8.3 Hz, 4H), 4.40 (d, J = 7.2 Hz, 4H), 2.30 (q, J = 7.7 Hz, 8H), 2.16 (d_t, J = 15.1 Hz, J = 7.2 Hz, 8H), 1.55 (s, 12H), 1.51 – 1.41 (m, 8H), 1.37 (s, 12H).

^{13}C NMR (126 MHz, Methanol-d_4, 298K) δ 156.8, 146.9, 145.9, 137.9, 132.6, 129.5, 126.7, 124.1, 107.5, 101.6, 63.1, 36.8, 36.4, 35.4, 34.3, 32.0, 29.6, 25.3.

IR ν_{max}(thin film)/cm^{-1} 3246.5m, 2834.0w, 1613.7m, 1487.9s, 1290.9m, 1177.2m, 1042.9m, 962.0s, 681.1s.

HR-MS (ESI$^+$, methanol): [M]$^{4+}$: $C_{104}H_{107}N_8O_8^{4+}$, calculated *m/z* 399.2067, found *m/z* 399.2075.

4-*1*-Methyl-imidazolium-butyl-acridane[4]arene (19)

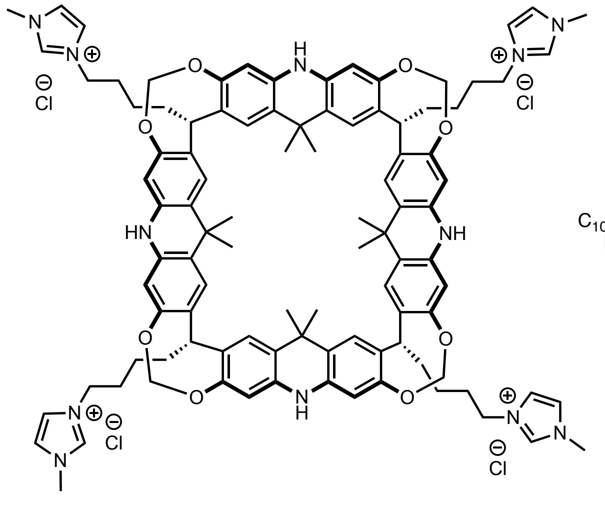

$C_{100}H_{112}N_{12}O_8Cl_4$
M= 1610.07

19

A solution of **17** (24.0 mg, 16.9 µmol, 1.00 equiv.) in 1-methyl-imidazole (1.10 mL) under an argon atmosphere was stirred at 90 °C for 48h. The 1-methyl-imidazole was removed under high vacuum and the solid was refluxed with acetone (10 mL) for 2h. The solid was separated, washed with acetone and dried under high vacuum to afford **19** (27.1 mg, 16.9 µmol, quant.).

^{1}H NMR (500 MHz, Methanol-d_4, 298K) δ 7.60 (d, *J* = 1.9 Hz, 4H), 7.53 (d, *J* = 1.9 Hz, 4H), 7.21 (s, 8H), 6.16 (s, 8H), 5.74 (d, *J* = 7.2 Hz, 4H), 4.62 (t, *J* = 8.2 Hz, 4H), 4.40 (d, *J* = 7.3 Hz, 4H), 4.23 (t, *J* = 7.1 Hz, 8H), 3.98 (s, 12H), 2.28 (q, *J* = 7.7 Hz, 8H), 2.02 (p, *J* = 7.3 Hz, 8H), 1.59 (s, 12H), 1.43 (q, *J* = 7.6 Hz, 8H), 1.39 (s, 12H).

^{13}C NMR (126 MHz, Methanol-d_4, 298K) δ 155.4, 136.5, 131.3, 125.3, 123.5, 122.8, 122.2, 106.1, 99.7, 49.4, 47.7, 35.5, 35.0, 33.9, 32.9, 29.3, 28.3, 24.1.

IR ν_{max}(thin film)/cm^{-1} 3076.1w, 2926.8w, 1666.9m, 1614.2m, 1487.5s, 1289.6m, 1172.1s, 1129.7w, 1044.9m, 960.5s, 856.2w, 745.0m, 620.0m.

HR-MS (ESI^{+}, methanol): $[M]^{4+}$: $C_{100}H_{112}N_{12}O_8^{4+}$, calculated *m/z* 402.2176, found *m/z* 402.2176.

3. Reaction monitoring of the cyclisation towards Et-A4A (8a)

The samples were prepared by sampling 0.1mL from the reaction mixture and adding to 0.5mL acetone-d_6. The sample was subjected to ^{1}H-NMR spectroscopy. The formation of a second symmetric species can be deduced from the methine signals at approx. 4.3 ppm (highlighted in the grey box). HR-MS analysis confirms the initial formation of the trimeric macrocycle.

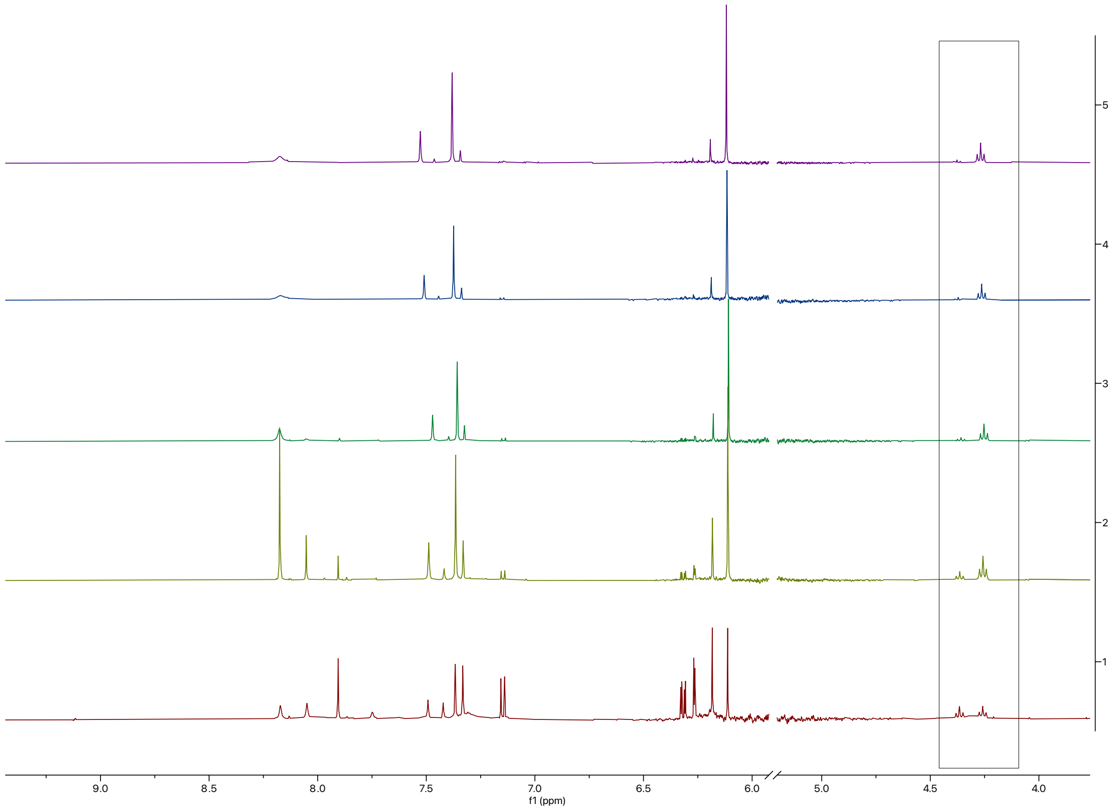

Figure S1: Excerpts from the 1H-NMR spectra in acetone-d_6 of the cyclisation reaction of 8a, where the formation of the two different macrocycles can be deduced from the methine signals, highlighted by the grey box.

4. Guest Uptake Studies

Different potential guests for the dimeric cage **10_2** were tested, including coronene, perylene, 1,1’-binaphtalene, methylene-bridged methyl-resorcin[4]arene, fullerene-C_{60}, estradiol, andrastenedione, estrone, tetrabutylammonium bromide TBAB, tetrabutylammonium iodide TBAI and tetrabutylammonium hexafluorophosphate. However, only for the tetrabutylammonium salts, guest-uptake was observed as indicated by the appearance of a new signals between approx. -1.2 and 0 ppm. The anion had no influence on the shape or chemical shift of the signals.

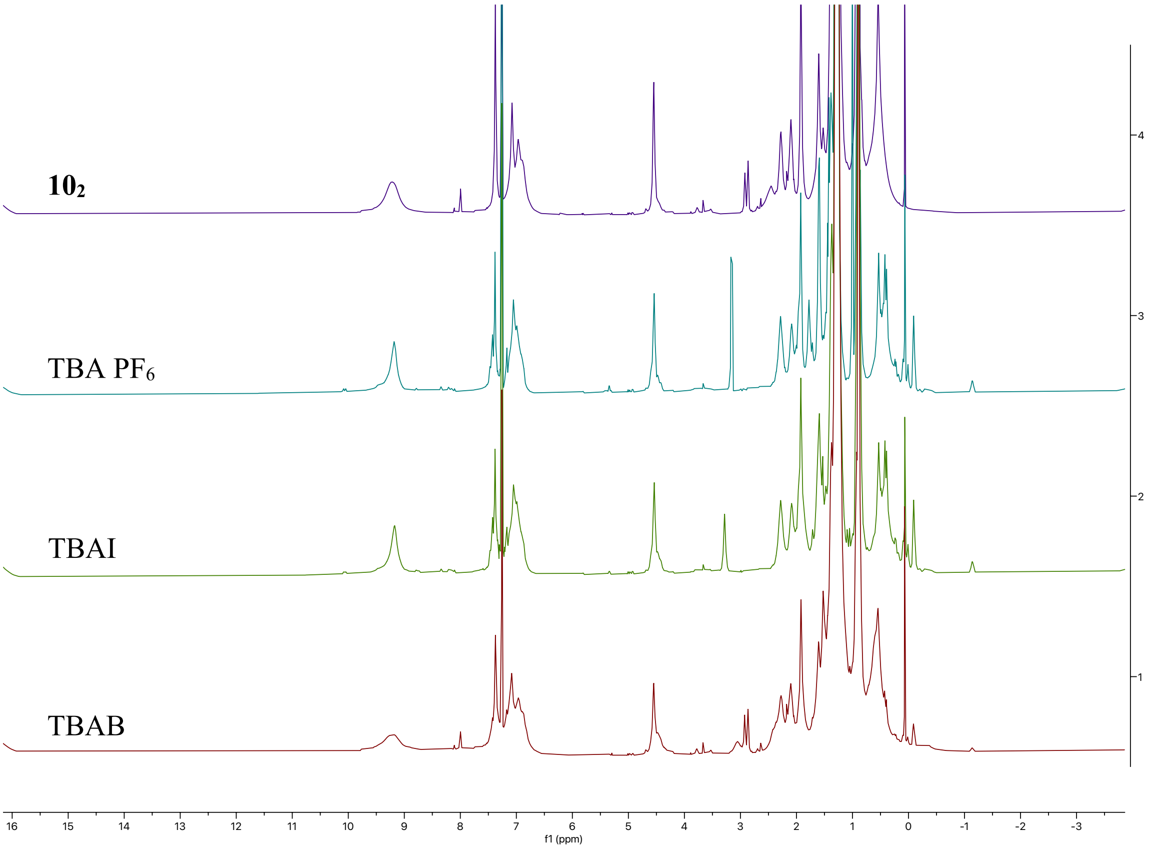

Figure S2: ^{1}H NMR of 10_2 (10mM) with 0.5 equiv. of different tetrabutylammonium salts.

An NMR-titration was performed by measuring ^{1}H NMR spectra at 298K and chloroform-*d* as a solvent. A 20mM stock solution of **10** in chloroform-*d* was prepared. Furthermore, a 250mM TBAB solution was prepared, and added portionwise to the stock solution of **10**. After mixing the solution by shaking and waiting for 5 min, the spectrum was recorded.

Upon the formation of the host-guest complex, a strong shift of the TBAB signals is observed. The most striking signal is appearing at -0.09 ppm. During the titration, this signal disappears again when TBAB is added in excess, very likely due to the disassembly of the complex due to the polar additive. Since the chemical shifts of the resonances corresponding to the encapsulated guest remain unchanged across guest concentrations (0.25–1.0 equiv), the system likely exhibits slow exchange on the ^{1}H NMR time scale.
The binding constant was estimated from this data. The complex is formed only within a narrow concentration window and dissociates at higher equivalents of the polar guest or at lower host concentrations. Within this specific range (10 mM host, 0.25 equiv guest), complete guest uptake was observed. As the exchange is slow on the ^{1}H NMR time scale, only a lower limit of the binding constant could be determined, assuming that a maximum of 2% of the guest remains unbound (NMR detection limit). Under these conditions, the binding constant is estimated to be at least 6500 M^{-1} for TBAB.

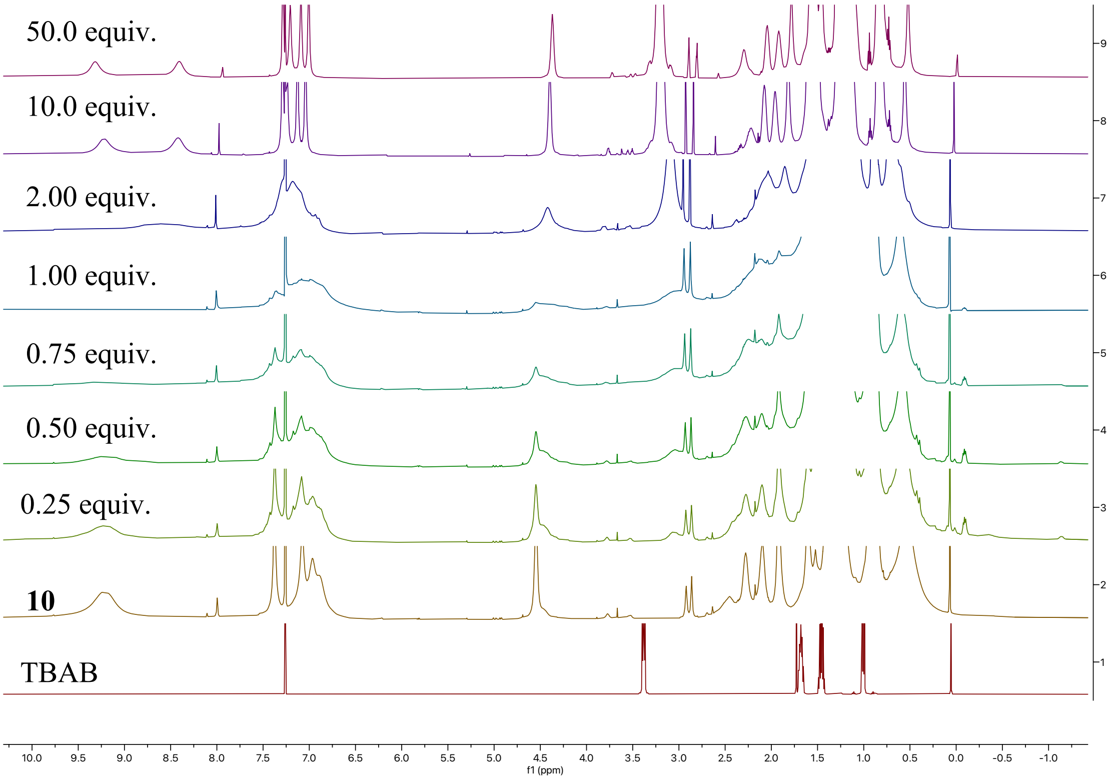

Figure S3: ^{1}H titration of 10_2 with TBAB.

5. DOSY-NMR Studies

The diffusion coefficients of **9** (N,O-Boc), **10** (N-Boc) and the host-guest complex TBAB@$\mathbf{10_2}$ were determined by using 20mM solutions of the respective compounds in chloroform-*d*, and the coefficients for **9** and **10** were determined for all major peaks. The diffusion coefficient for TBAB@$\mathbf{10_2}$ (0.25 equiv.) was determined for the signal at -0.09 ppm. The results are shown in Table S1 and show different values for macrocycle **9** and the dimeric cage **10**. Upon formation of TBAB@$\mathbf{10_2}$ the diffusion coefficient of the complex signals remains low. Due to the entrapped guest, it diffuses even slower than the empty cage-like structure.

Table S1: Diffusion values for 9, 10 and TBAB@10 in chloroform-d at 289K.

Macrocycle	**9**	**10**	TBAB@$\mathbf{10_2}$
D [$\times 10^{-5}$ $cm^2 s^{-1}$]	0.32	0.19	0.13

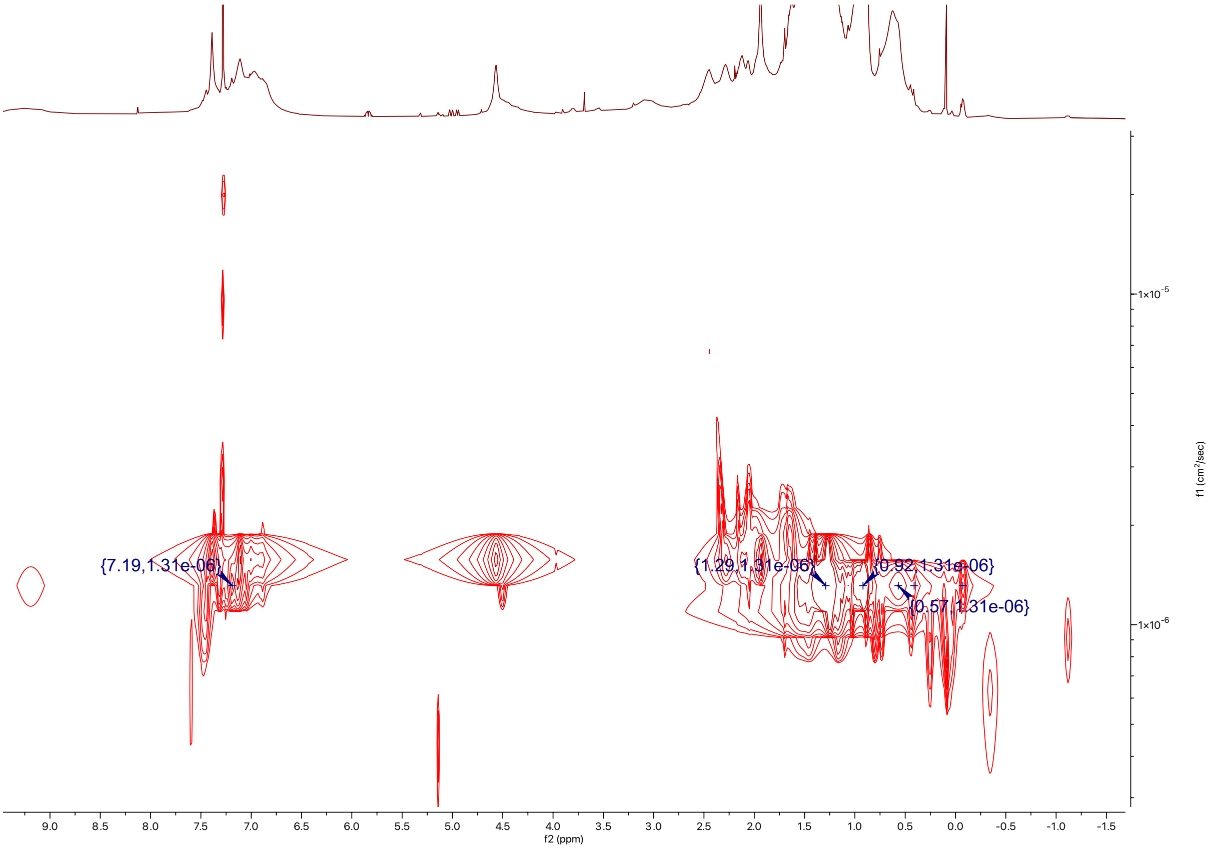

Figure S4: DOSY-NMR of TBAB@$\mathbf{10_2}$.

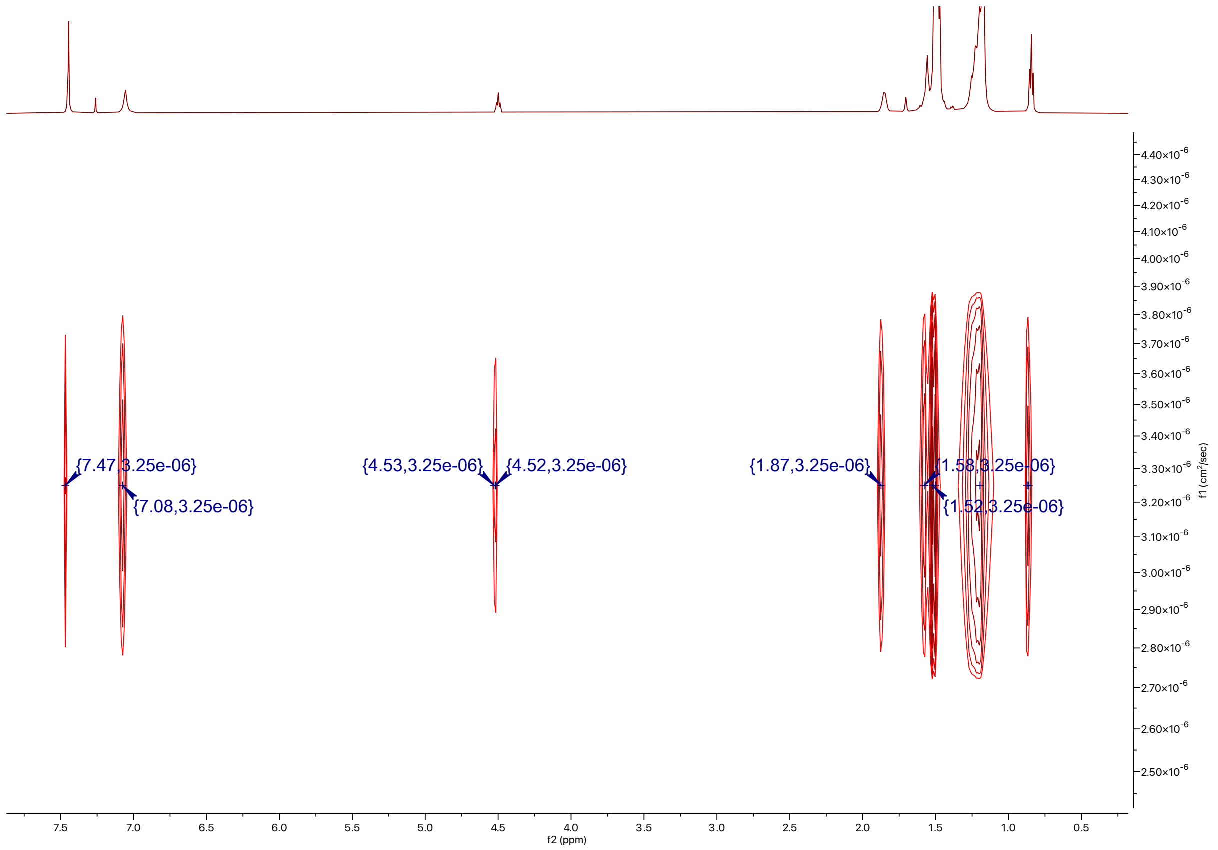

Figure S5: DOSY-NMR of 9.

5.1 Calculation of the hydrodynamic radius

The hydrodynamic radius r_h of the dimeric cage $\mathbf{10_2}$ in chloroform-*d* was estimated using a semi-empirical approach towards the modified Stokes-Einstein equation (equation 1)[3] and solved numerically using the Math Input-function of Wolfram Alpha.[a]

$$D = \frac{k_B T}{\left(\frac{6}{1+0.695\left(\frac{r_{solv}}{r_h}\right)^{2.234}}\right) * \pi \eta r_h} \qquad \textbf{Equation 1}$$

D = Diffusion coefficient obtained from DOSY-measurements [0.19×10^{-5} cm^2s^{-1}]

k_B = Boltzmann constant [$1.3806485 \times 10^{-23}$ $m^2kgs^{-1}K^{-1}$]

T = Temperature [298 K]

r_{solv} = Hydrodynamic radius of the solvent [0.260 nm]

r_h = Hydrodynamic radius of the analyte [m]

η = Viscosity of the solvent at 298 K [$0.542\ 10^{-3}$ $kgm^{-1}s^{-1}$]

$$r_h = 25.8\ \text{Å}$$

The hydrodynamic radius is also estimated with the molecular model build in the Spartan'24 software (Wavefunction, Inc). Here, a hydrodynamic radius of 25 Å is measured using the Pymol software.

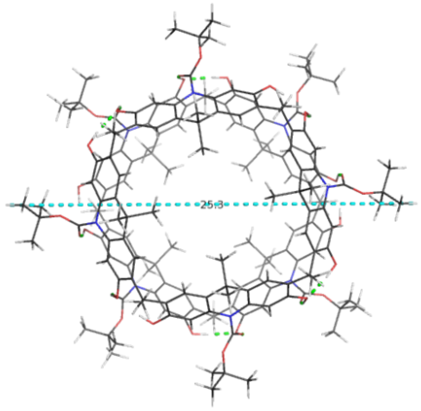

Figure S6: Model of TBAB@10_2

[a]https://www.wolframalpha.com/input?i2d=true&i=0.19+*+%5C%2840%29Power%5B10%2C%5C%2840%29-+5%5C%2841%29%5D%5C%2841%29+%3D+Divide%5B%5C%2840%291.3806485+*+%5C%2840%29Power%5B10%2C%5C%2840%29+-+23%5C%2841%29%5D%5C%2841%29+*298%5C%2841%29%2C%5C%2840%29Divide%5B6%2C%5C%2840%291%2B0.695+*+Power%5B%5C%2840%29+Divide%5B0.260+*+%5C%2840%29Power%5B10%2C%5C%2840%29+-+9%5C%2841%29%5D%5C%2841%29%2Cx%5D%5C%2841%29%2C2.234%5D%5C%2841%29%5D%5C%2841%29*π*x*0.542*Power%5B10%2C%5C%2840%29-3%5C%2841%29%5D%5D

6. Crystallographic Data of Cl-A4A (8b)

checkCIF/PLATON report

Structure factors have been supplied for datablock(s) vh123_150k

THIS REPORT IS FOR GUIDANCE ONLY. IF USED AS PART OF A REVIEW PROCEDURE FOR PUBLICATION, IT SHOULD NOT REPLACE THE EXPERTISE OF AN EXPERIENCED CRYSTALLOGRAPHIC REFEREE.

No syntax errors found. CIF dictionary Interpreting this report

Datablock: vh123_150k

Bond precision: C-C = 0.0113 A Wavelength=1.34143

Cell:	a=14.8708(4)	b=17.1967(4)	c=19.4680(5)
	alpha=82.280(2)	beta=70.150(2)	gamma=86.264(2)
Temperature:	150 K		

	Calculated	Reported
Volume	4639.3(2)	4639.3(2)
Space group	P -1	P -1
Hall group	-P 1	-P 1
Moiety formula	C80 H87.30 Cl4 N4 O8, C2.40 H4.80 O0.80, 5(C3 H6 O)	C80 H87.3 Cl4 N4 O8, 5.8(C3 H6 O)
Sum formula	C97.40 H122.10 Cl4 N4 O13.80	C97.40 H122.10 Cl4 N4 O13.80
Mr	1711.50	1711.48
Dx,g cm-3	1.225	1.225
Z	2	2
Mu (mm-1)	1.095	1.095
F000	1825.8	1826.0
F000'	1832.15	
h,k,lmax	18,21,23	18,21,23
Nref	18159	17724
Tmin,Tmax	0.762,0.947	0.004,0.098
Tmin'	0.720	

Correction method= # Reported T Limits: Tmin=0.004 Tmax=0.098
AbsCorr = MULTI-SCAN

Data completeness= 0.976 Theta(max)= 55.722

R(reflections)= 0.1364(8538) wR2(reflections)= 0.3336(17724)

S = 1.091 Npar= 1117

The following ALERTS were generated. Each ALERT has the format
test-name_ALERT_alert-type_alert-level.
Click on the hyperlinks for more details of the test.

Alert level B

```
PLAT340_ALERT_3_B Low Bond Precision on  C-C Bonds ..............    0.01129 Ang.
```

Author Response: Given the large presence of solvent molecules, disorder and weak diffraction at medium and high angles, this is not unexpected.

Alert level C

```
RINTA01_ALERT_3_C  The value of Rint is greater than 0.12
            Rint given   0.179
PLAT020_ALERT_3_C The Value of Rint is Greater Than 0.12 .........      0.179 Report
PLAT026_ALERT_3_C Ratio Observed / Unique Reflections (too) Low ..        48% Check
PLAT042_ALERT_1_C Calc. and Reported MoietyFormula Strings  Differ     Please Check
PLAT077_ALERT_4_C Unitcell Contains Non-integer Number of Atoms ..     Please Check
PLAT082_ALERT_2_C High R1 Value ..................................       0.14 Report
PLAT084_ALERT_3_C High wR2 Value (i.e. > 0.25) ...................       0.33 Report
PLAT220_ALERT_2_C NonSolvent   Resd 1  C   Ueq(max)/Ueq(min) Range        3.4 Ratio
PLAT220_ALERT_2_C NonSolvent   Resd 1 Cl   Ueq(max)/Ueq(min) Range        3.7 Ratio
PLAT241_ALERT_2_C High   'MainMol' Ueq as Compared to Neighbors of        C71 Check
PLAT244_ALERT_4_C Low    'Solvent' Ueq as Compared to Neighbors of        C98 Check
PLAT244_ALERT_4_C Low    'Solvent' Ueq as Compared to Neighbors of        C92 Check
PLAT244_ALERT_4_C Low    'Solvent' Ueq as Compared to Neighbors of        C95 Check
PLAT250_ALERT_2_C Large U3/U1 Ratio for Average U(i,j) Tensor ....        2.9 Note
PLAT260_ALERT_2_C Large Average Ueq of Residue Including       O9      0.112 Check
PLAT260_ALERT_2_C Large Average Ueq of Residue Including      O11      0.108 Check
PLAT260_ALERT_2_C Large Average Ueq of Residue Including      O13      0.132 Check
PLAT336_ALERT_2_C Long Bond Distance for ..... C74      -Cl3          1.861 Ang.
PLAT360_ALERT_2_C Short  C(sp3)-C(sp3) Bond  C58      - C59      .     1.43 Ang.
PLAT360_ALERT_2_C Short  C(sp3)-C(sp3) Bond  C73      - C74      .     1.37 Ang.
PLAT362_ALERT_2_C Short  C(sp3)-C(sp2) Bond  C92      - C93      .     1.40 Ang.
PLAT362_ALERT_2_C Short  C(sp3)-C(sp2) Bond  C83      - C85      .     1.41 Ang.
PLAT414_ALERT_2_C Short Intra D-H..H-X       H3       ..H12      .     1.97 Ang.
                                                      x,y,z  =     1_555 Check
PLAT420_ALERT_2_C D-H Bond Without Acceptor  N4       --H4A      .    Please Check
PLAT420_ALERT_2_C D-H Bond Without Acceptor  N5       --H5A      .    Please Check
PLAT906_ALERT_3_C Large K Value in the Analysis of Variance ......     11.725 Check
PLAT906_ALERT_3_C Large K Value in the Analysis of Variance ......      2.801 Check
PLAT911_ALERT_3_C Missing FCF Refl Between Thmin & STh/L=    0.600         88 Report
PLAT918_ALERT_3_C Reflection(s) with I(obs) much Smaller I(calc) .          2 Check
```

Alert level G

```
ABSMU01_ALERT_1_G  Calculation of _exptl_absorpt_correction_mu
```

```
              not performed for this radiation type.
PLAT002_ALERT_2_G Number of Distance or Angle Restraints on AtSite         30 Note
PLAT003_ALERT_2_G Number of Uiso or Uij Restrained non-H Atoms ...         29 Report
PLAT007_ALERT_5_G Number of Unrefined Donor-H Atoms .............          12 Report
PLAT068_ALERT_1_G Reported F000 Differs from Calcd (or Missing)...     Please Check
PLAT083_ALERT_2_G SHELXL Second Parameter in WGHT  Unusually Large      15.00 Why ?
PLAT154_ALERT_1_G The s.u.'s on the Cell Angles are Equal ..(Note)      0.002 Degree
PLAT171_ALERT_4_G The CIF-Embedded .res File Contains EADP Records          3 Report
PLAT173_ALERT_4_G The CIF-Embedded .res File Contains DANG Records          1 Report
PLAT176_ALERT_4_G The CIF-Embedded .res File Contains SADI Records          6 Report
PLAT178_ALERT_4_G The CIF-Embedded .res File Contains SIMU Records          4 Report
PLAT180_ALERT_4_G Check Cell Rounding: # of Values Ending with 0 =          3 Note
PLAT186_ALERT_4_G The CIF-Embedded .res File Contains ISOR Records          2 Report
PLAT187_ALERT_4_G The CIF-Embedded .res File Contains RIGU Records          4 Report
PLAT300_ALERT_4_G Atom Site Occupancy of C14       Constrained at       0.65 Check
PLAT300_ALERT_4_G Atom Site Occupancy of C15       Constrained at       0.35 Check
PLAT300_ALERT_4_G Atom Site Occupancy of C65       Constrained at       0.65 Check
PLAT300_ALERT_4_G Atom Site Occupancy of C66       Constrained at       0.65 Check
PLAT300_ALERT_4_G Atom Site Occupancy of C67       Constrained at       0.35 Check
PLAT300_ALERT_4_G Atom Site Occupancy of C68       Constrained at       0.35 Check
PLAT300_ALERT_4_G Atom Site Occupancy of H64A      Constrained at       0.65 Check
PLAT300_ALERT_4_G Atom Site Occupancy of H64B      Constrained at       0.65 Check
PLAT300_ALERT_4_G Atom Site Occupancy of H65A      Constrained at       0.65 Check
PLAT300_ALERT_4_G Atom Site Occupancy of H65B      Constrained at       0.65 Check
PLAT300_ALERT_4_G Atom Site Occupancy of H66A      Constrained at       0.65 Check
PLAT300_ALERT_4_G Atom Site Occupancy of H66B      Constrained at       0.65 Check
PLAT300_ALERT_4_G Atom Site Occupancy of H67A      Constrained at       0.35 Check
PLAT300_ALERT_4_G Atom Site Occupancy of H67B      Constrained at       0.35 Check
PLAT300_ALERT_4_G Atom Site Occupancy of H68A      Constrained at       0.35 Check
PLAT300_ALERT_4_G Atom Site Occupancy of H68B      Constrained at       0.35 Check
PLAT300_ALERT_4_G Atom Site Occupancy of O10       Constrained at        0.8 Check
PLAT300_ALERT_4_G Atom Site Occupancy of C89       Constrained at        0.5 Check
PLAT300_ALERT_4_G Atom Site Occupancy of C90       Constrained at        0.5 Check
PLAT300_ALERT_4_G Atom Site Occupancy of C91       Constrained at        0.5 Check
PLAT300_ALERT_4_G Atom Site Occupancy of C86       Constrained at        0.3 Check
PLAT300_ALERT_4_G Atom Site Occupancy of C87       Constrained at        0.3 Check
PLAT300_ALERT_4_G Atom Site Occupancy of C88       Constrained at        0.3 Check
PLAT300_ALERT_4_G Atom Site Occupancy of H89A      Constrained at        0.5 Check
PLAT300_ALERT_4_G Atom Site Occupancy of H89B      Constrained at        0.5 Check
PLAT300_ALERT_4_G Atom Site Occupancy of H89C      Constrained at        0.5 Check
PLAT300_ALERT_4_G Atom Site Occupancy of H91A      Constrained at        0.5 Check
PLAT300_ALERT_4_G Atom Site Occupancy of H91B      Constrained at        0.5 Check
PLAT300_ALERT_4_G Atom Site Occupancy of H91C      Constrained at        0.5 Check
PLAT300_ALERT_4_G Atom Site Occupancy of H87A      Constrained at        0.3 Check
PLAT300_ALERT_4_G Atom Site Occupancy of H87B      Constrained at        0.3 Check
PLAT300_ALERT_4_G Atom Site Occupancy of H87C      Constrained at        0.3 Check
PLAT300_ALERT_4_G Atom Site Occupancy of H88A      Constrained at        0.3 Check
PLAT300_ALERT_4_G Atom Site Occupancy of H88B      Constrained at        0.3 Check
PLAT300_ALERT_4_G Atom Site Occupancy of H88C      Constrained at        0.3 Check
PLAT301_ALERT_3_G Main Residue  Disorder .............(Resd  1  )         3% Note
PLAT302_ALERT_4_G Anion/Solvent/Minor-Residue Disorder (Resd  2  )       100% Note
PLAT304_ALERT_4_G Non-Integer Number of Atoms in ..... (Resd  1  )     183.30 Check
PLAT311_ALERT_2_G Isolated Disordered Oxygen Atom (No H's ?) .....        O10 Check
PLAT367_ALERT_2_G Long?  C(sp?)-C(sp?) Bond  C63      - C64      .       1.51 Ang.
PLAT411_ALERT_2_G Short Inter H...H Contact  H59A     ..H67A     .       1.90 Ang.
                                                 1+x,y,z  =      1_655 Check
PLAT720_ALERT_4_G Number of Unusual/Non-Standard Labels ..........          9 Note
```

```
PLAT790_ALERT_4_G Centre of Gravity not Within Unit Cell: Resd.  #          6 Note
              C3 H6 O
PLAT860_ALERT_3_G Number of Least-Squares Restraints ............         360 Note
PLAT910_ALERT_3_G Missing # of FCF Reflection(s) Below Theta(Min).          2 Note
PLAT912_ALERT_4_G Missing # of FCF Reflections Above STh/L=  0.600        346 Note
PLAT933_ALERT_2_G Number of HKL-OMIT Records in Embedded .res File          7 Note
PLAT941_ALERT_3_G Average HKL Measurement Multiplicity ..........         4.0 Low
PLAT978_ALERT_2_G Number C-C Bonds with Positive Residual Density.          1 Info
```

```
   0 ALERT level A = Most likely a serious problem - resolve or explain
   1 ALERT level B = A potentially serious problem, consider carefully
  29 ALERT level C = Check. Ensure it is not caused by an omission or oversight
  63 ALERT level G = General information/check it is not something unexpected

   4 ALERT type 1 CIF construction/syntax error, inconsistent or missing data
  24 ALERT type 2 Indicator that the structure model may be wrong or deficient
  13 ALERT type 3 Indicator that the structure quality may be low
  51 ALERT type 4 Improvement, methodology, query or suggestion
   1 ALERT type 5 Informative message, check
```

It is advisable to attempt to resolve as many as possible of the alerts in all categories. Often the minor alerts point to easily fixed oversights, errors and omissions in your CIF or refinement strategy, so attention to these fine details can be worthwhile. In order to resolve some of the more serious problems it may be necessary to carry out additional measurements or structure refinements. However, the purpose of your study may justify the reported deviations and the more serious of these should normally be commented upon in the discussion or experimental section of a paper or in the "special_details" fields of the CIF. checkCIF was carefully designed to identify outliers and unusual parameters, but every test has its limitations and alerts that are not important in a particular case may appear. Conversely, the absence of alerts does not guarantee there are no aspects of the results needing attention. It is up to the individual to critically assess their own results and, if necessary, seek expert advice.

Publication of your CIF in IUCr journals

A basic structural check has been run on your CIF. These basic checks will be run on all CIFs submitted for publication in IUCr journals (*Acta Crystallographica*, *Journal of Applied Crystallography*, *Journal of Synchrotron Radiation*); however, if you intend to submit to *Acta Crystallographica Section C* or *E* or *IUCrData*, you should make sure that full publication checks are run on the final version of your CIF prior to submission.

Publication of your CIF in other journals

Please refer to the *Notes for Authors* of the relevant journal for any special instructions relating to CIF submission.

PLATON version of 06/07/2023; check.def file version of 30/06/2023

Datablock vh123_150k - ellipsoid plot

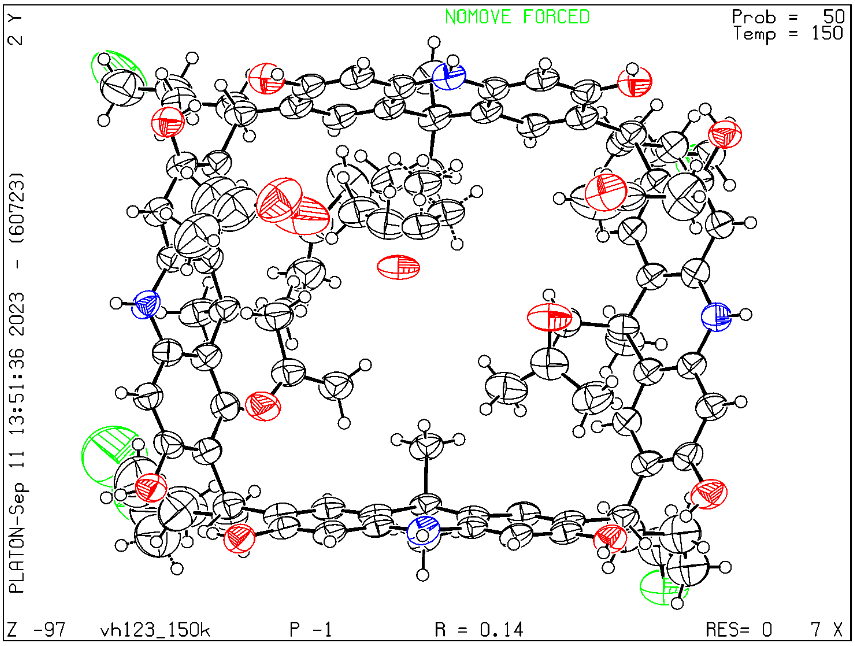

7. NMR-Spectra of New Compounds

^{1}H-NMR (500 MHz) and ^{13}C-NMR (126 MHz) spectrum of 6b in Acetone-d_6 at 298 K.

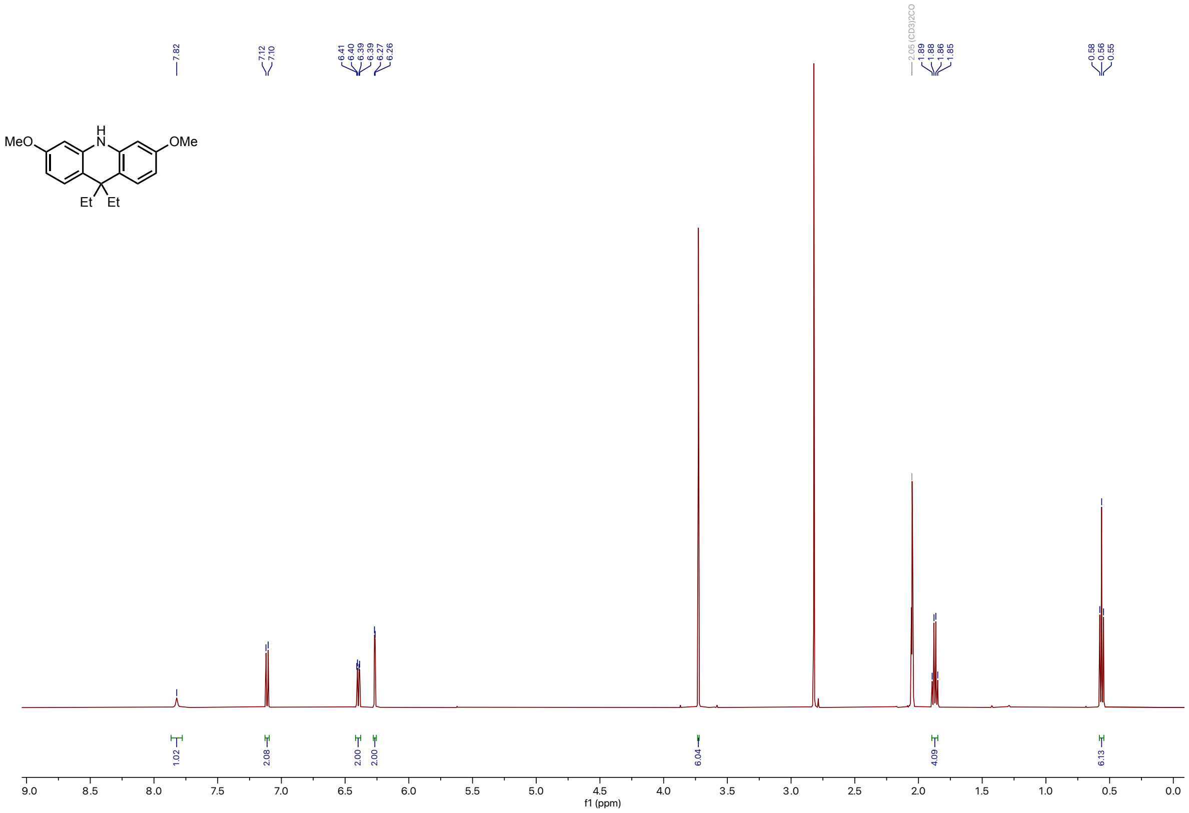

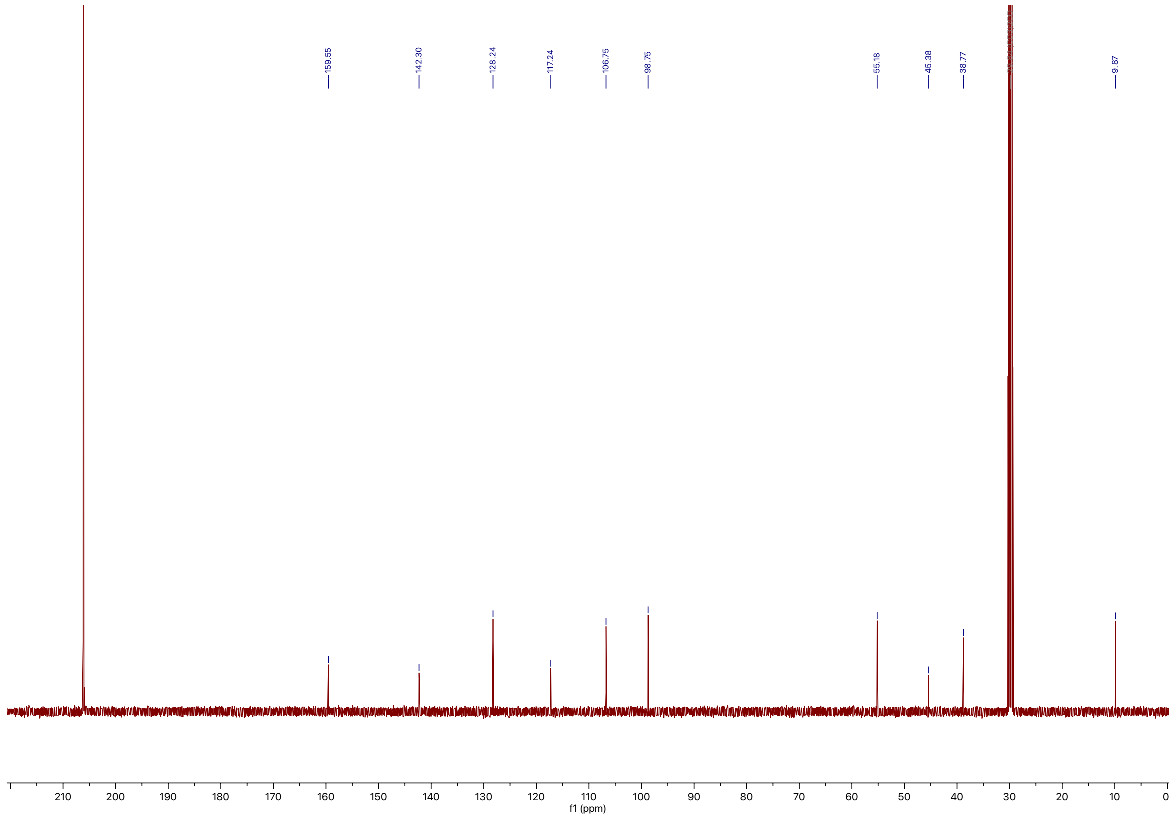

^{1}H-NMR (500 MHz) and ^{13}C-NMR (126 MHz) spectrum of 6c in Acetone-d_6 at 298 K.

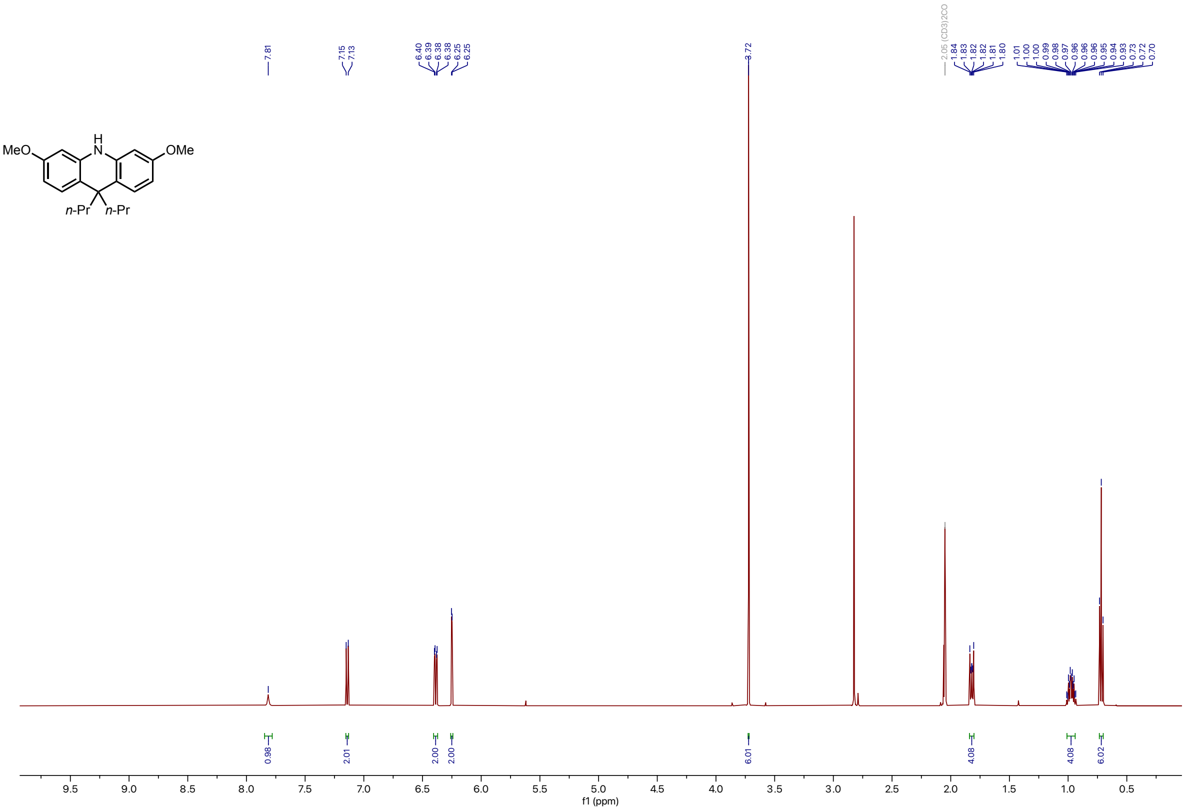

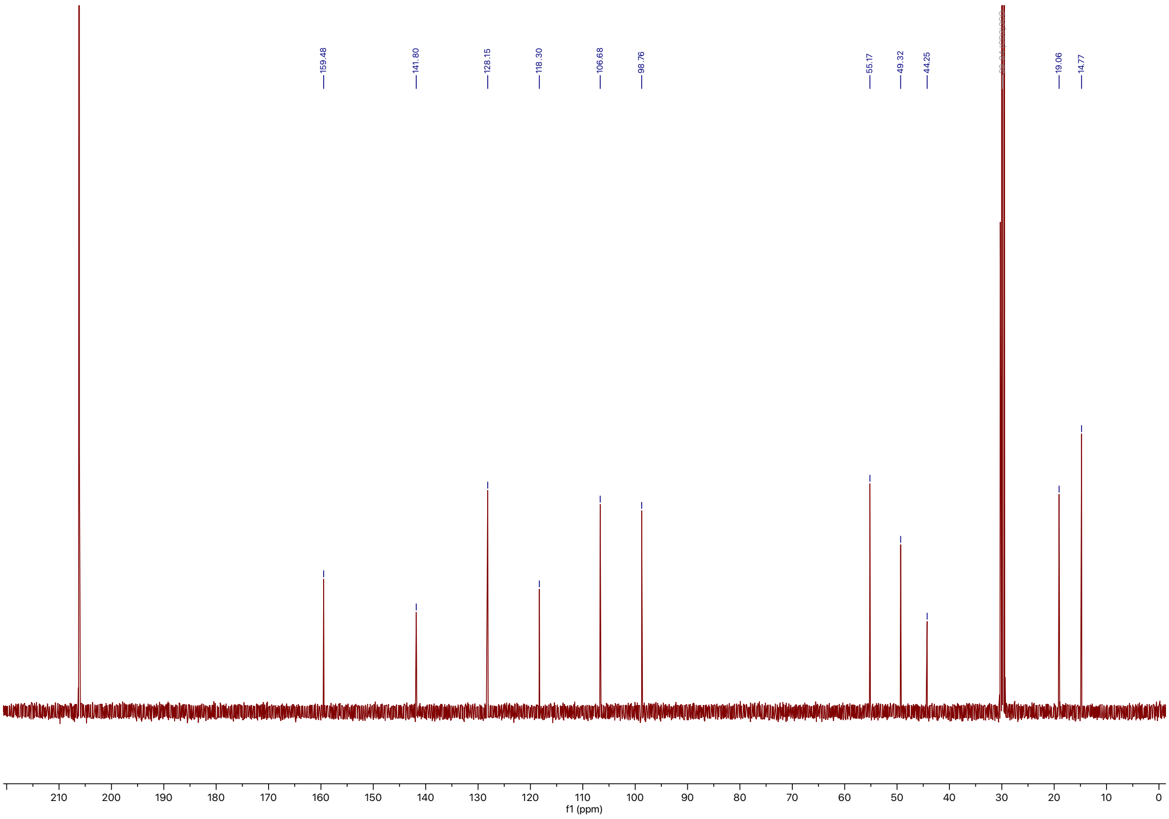

^{1}H-NMR (500 MHz) and ^{13}C-NMR (126 MHz) spectrum of 6d in Acetone-d_6 at 298 K.

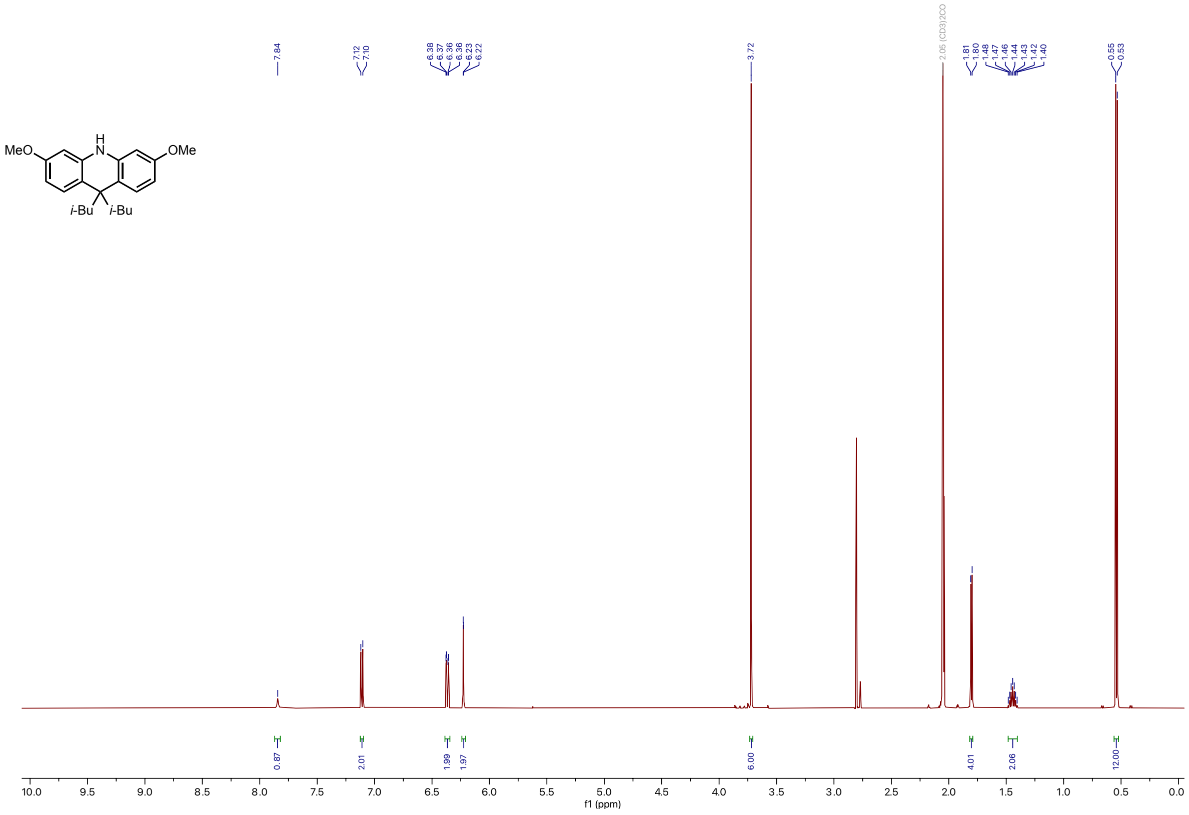

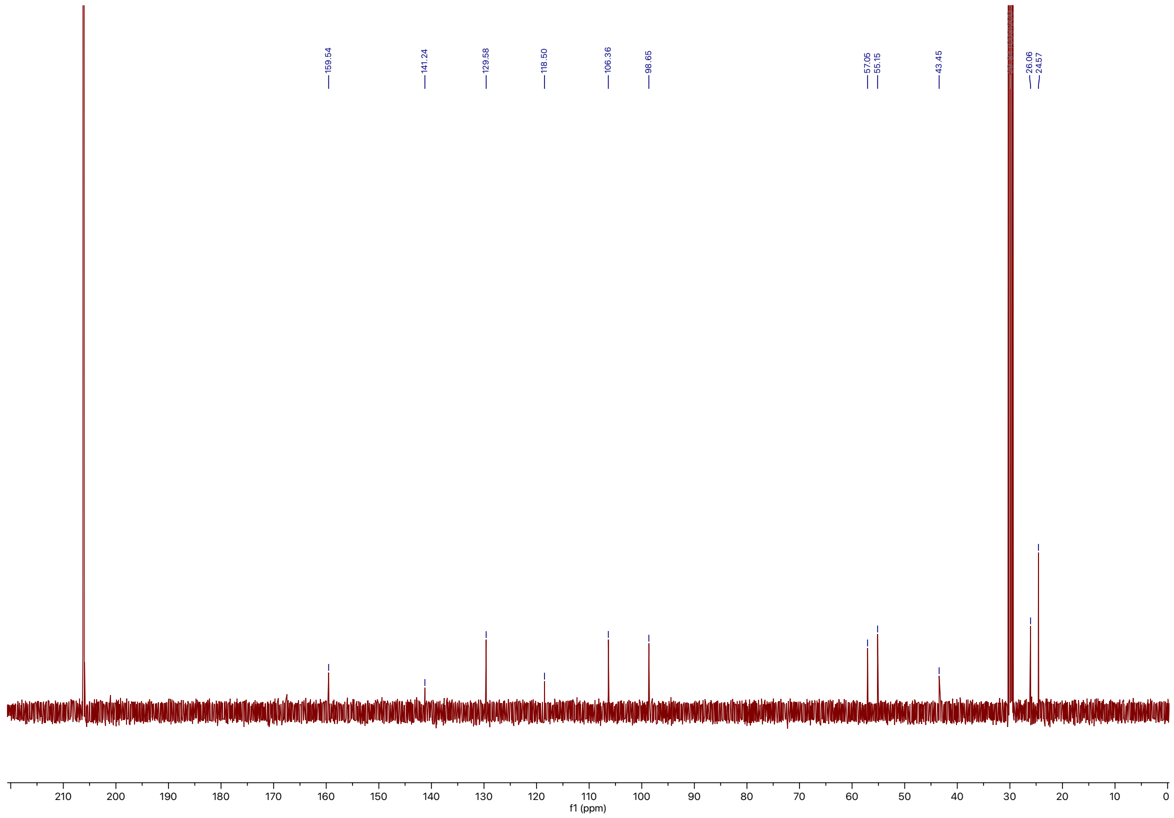

^{1}H-NMR (500 MHz) and ^{13}C-NMR (126 MHz) spectrum of 7b in Acetone-d_6 at 298 K

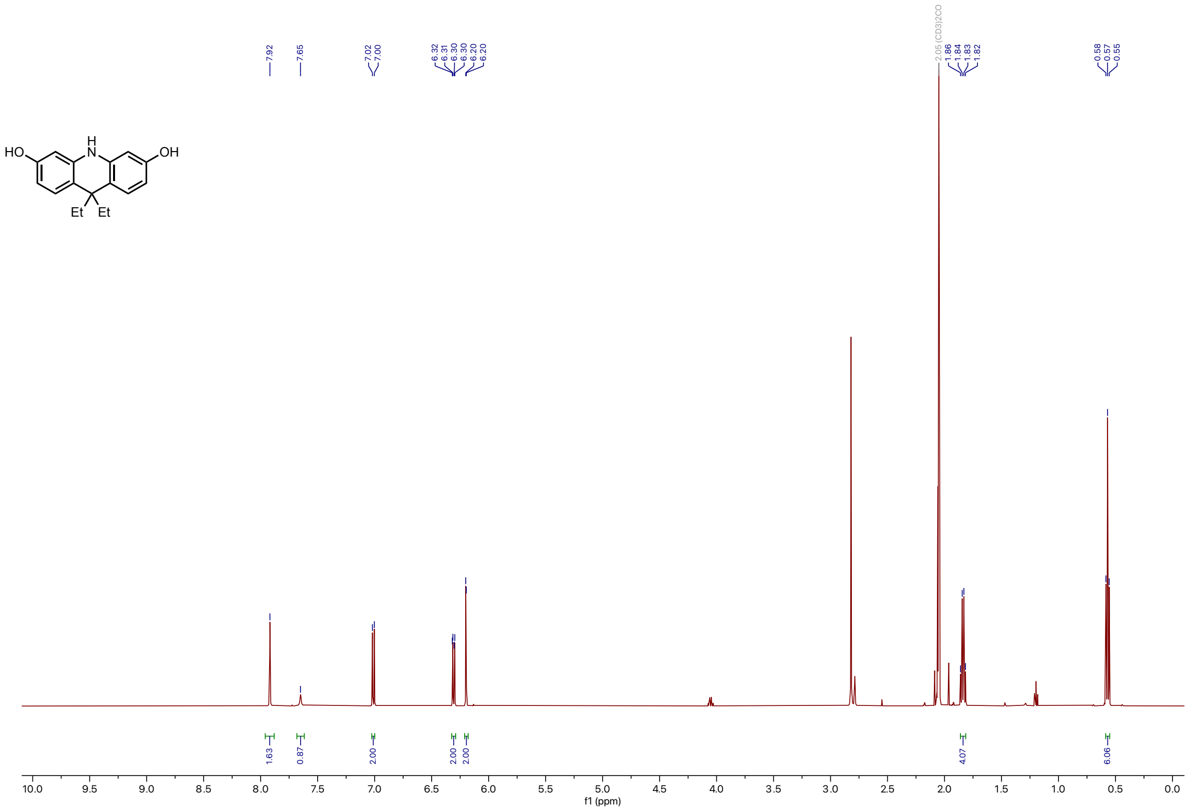

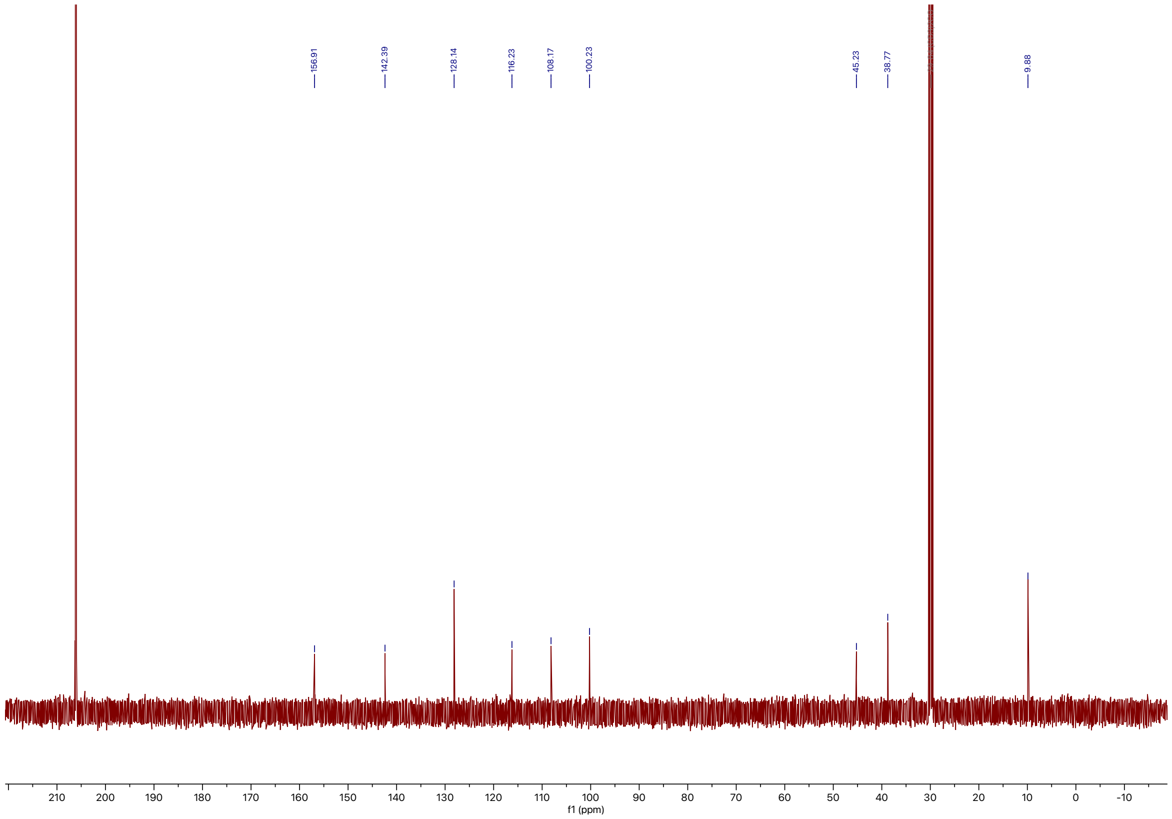

^{1}H-NMR (500 MHz) and ^{13}C-NMR (126 MHz) spectrum of 7c in Acetone-d_6 at 298 K.

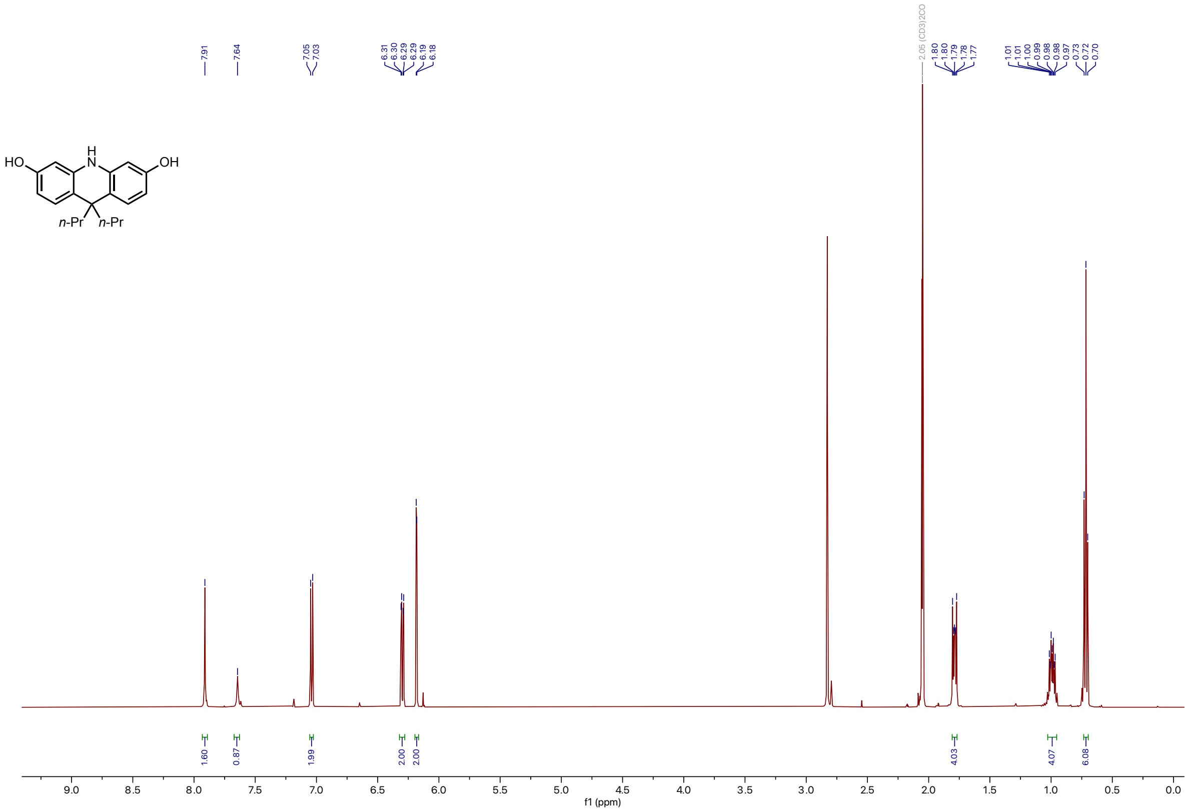

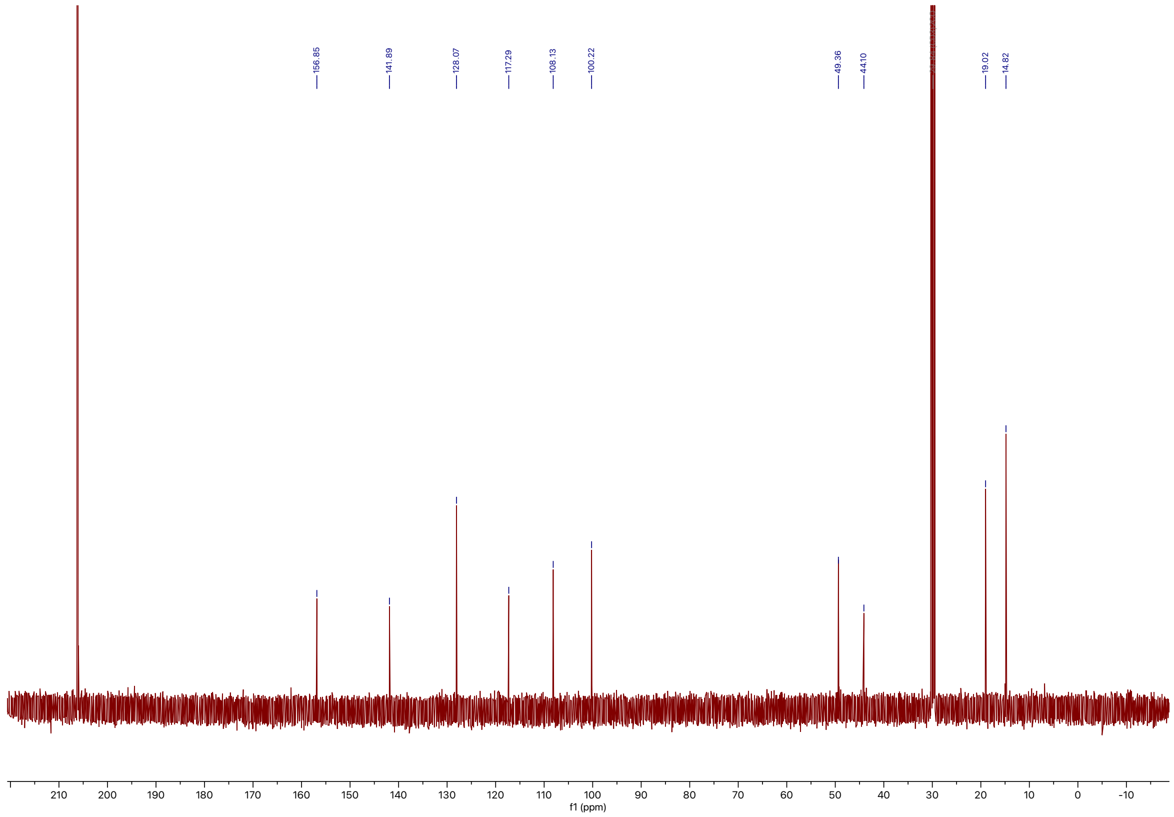

^{1}H-NMR (500 MHz) and ^{13}C-NMR (126 MHz) spectrum of 7d in Acetone-d_6 at 298 K.

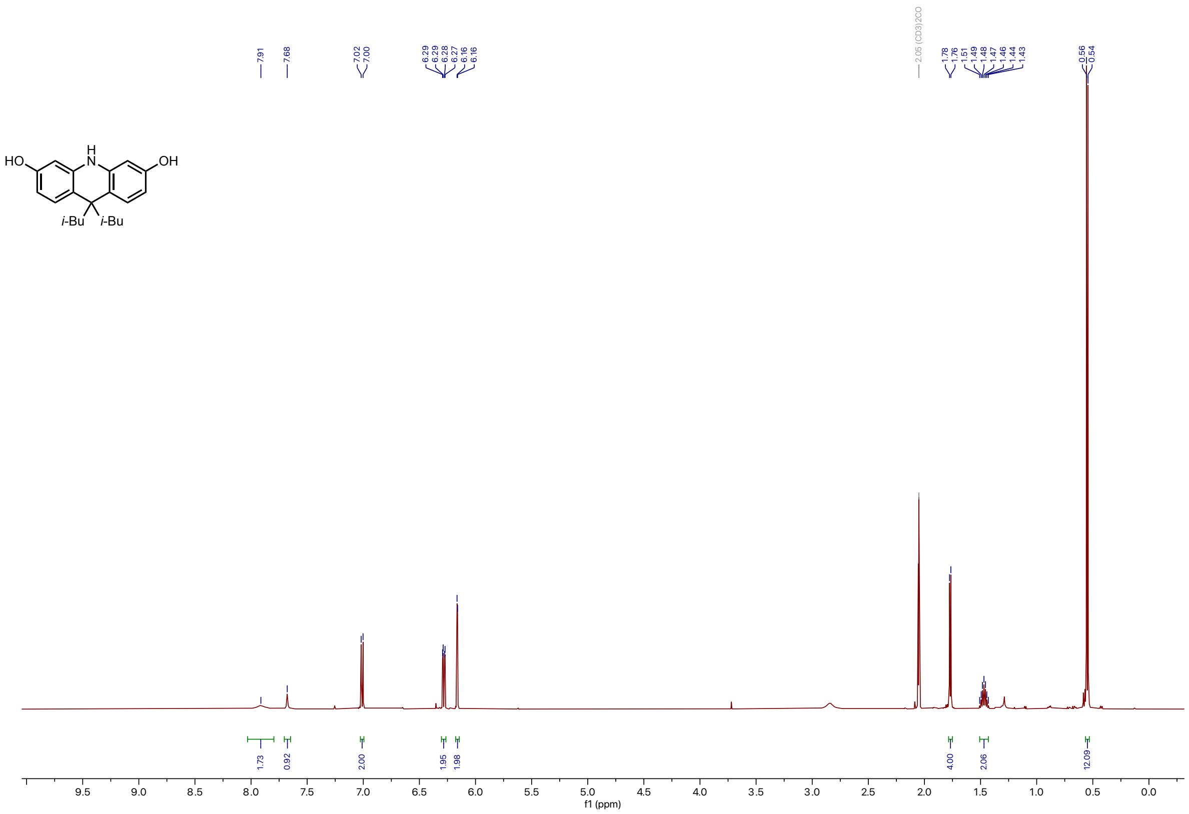

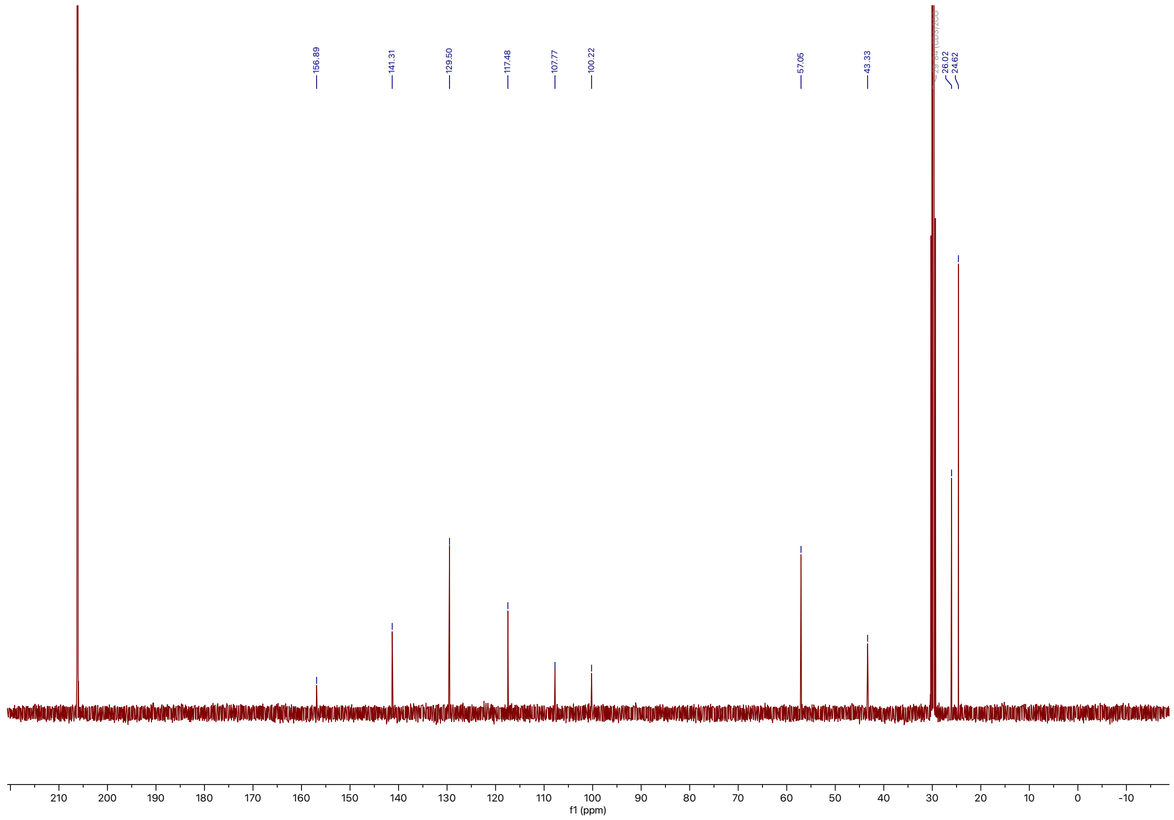

^{1}H-NMR (500 MHz) and ^{13}C-NMR (126 MHz) spectrum of 8a in Acetone-d_6 at 298 K.

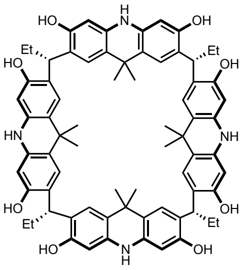

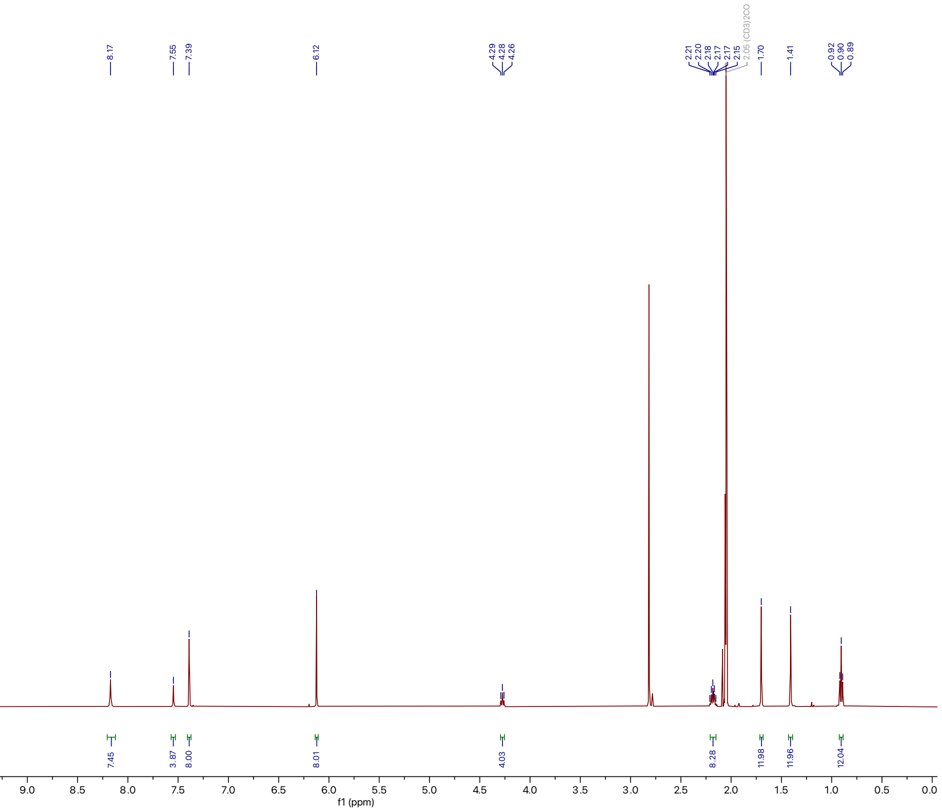

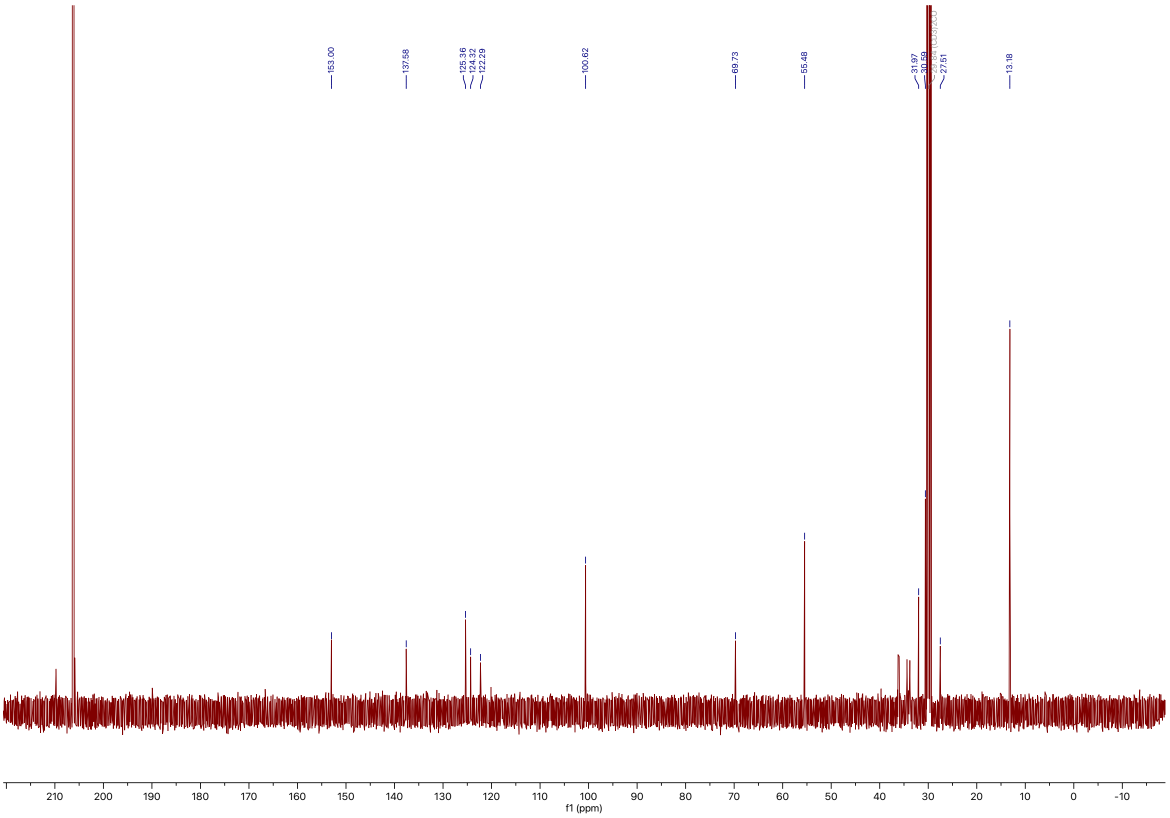

^{1}H-NMR (500 MHz) and ^{13}C-NMR (126 MHz) spectrum of 8b in Acetone-d_6 at 298 K.

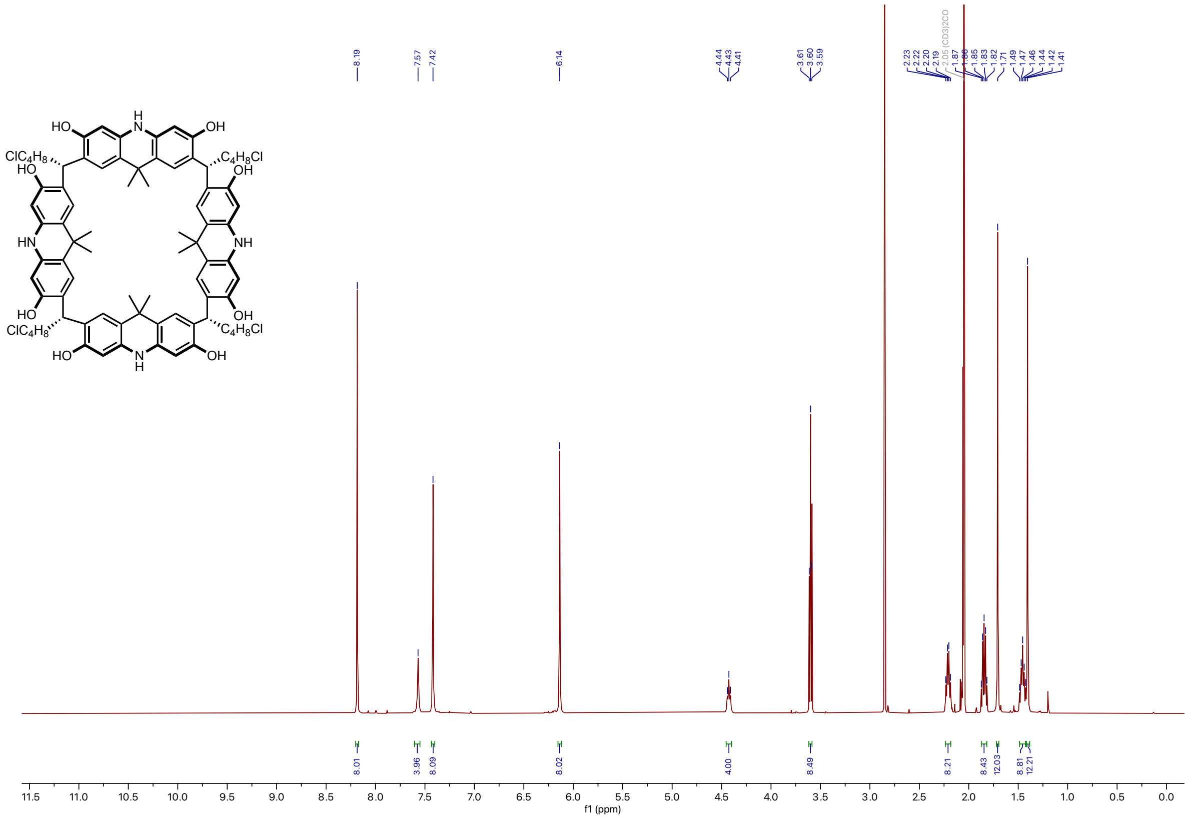

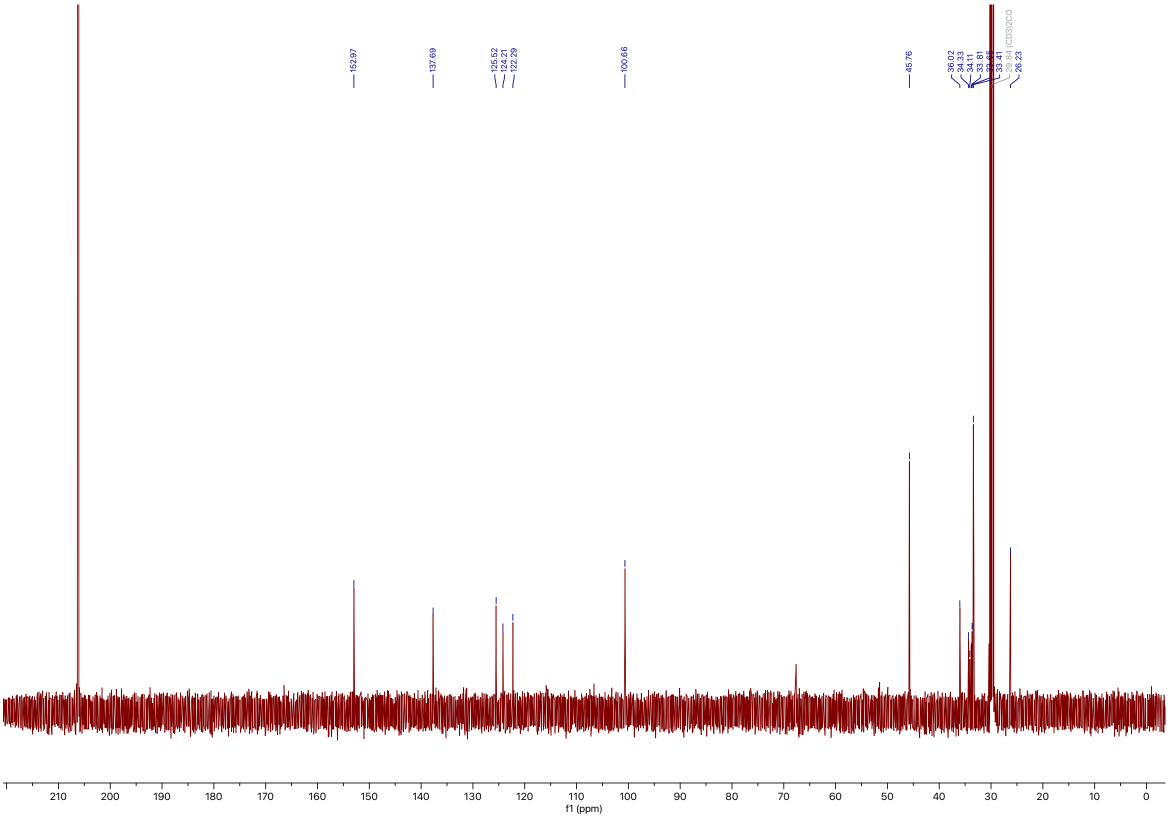

^{1}H-NMR (500 MHz) and ^{13}C-NMR (126 MHz) spectrum of 8e in Acetone-d_6 at 298 K.

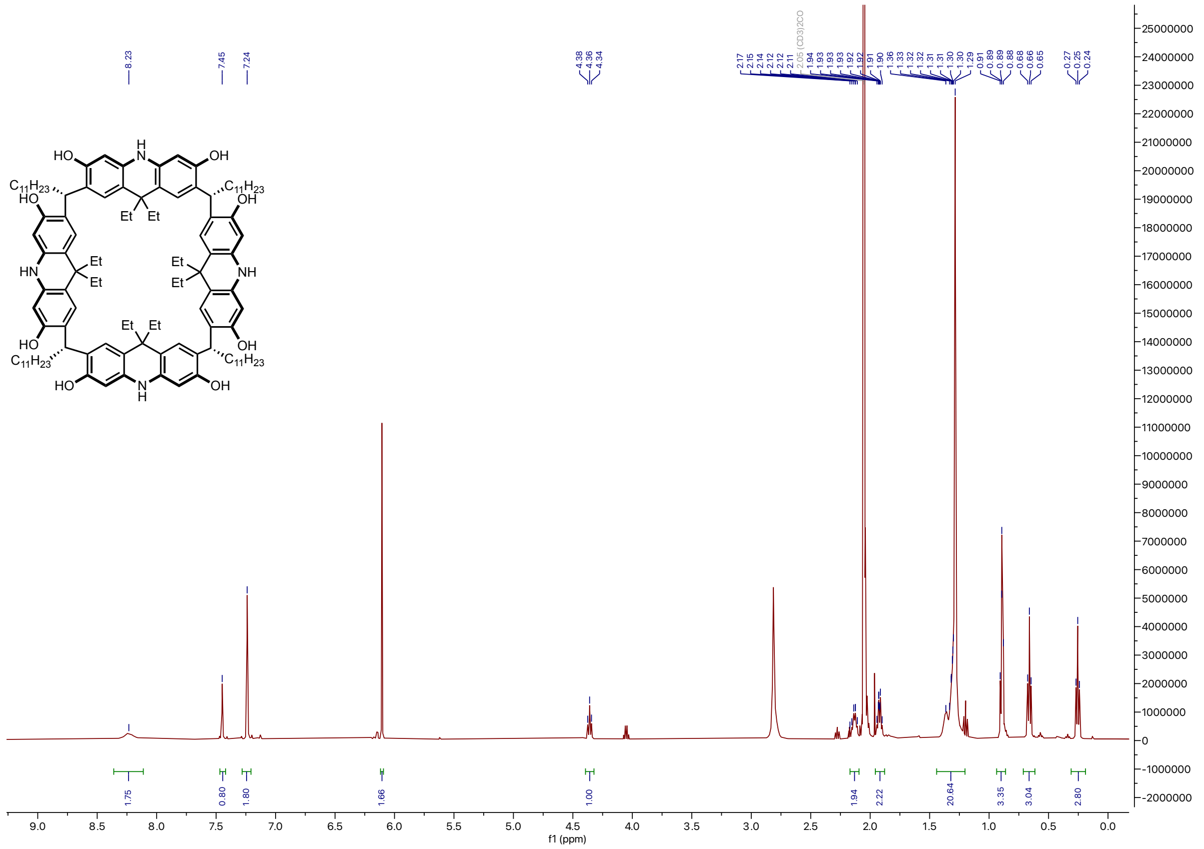

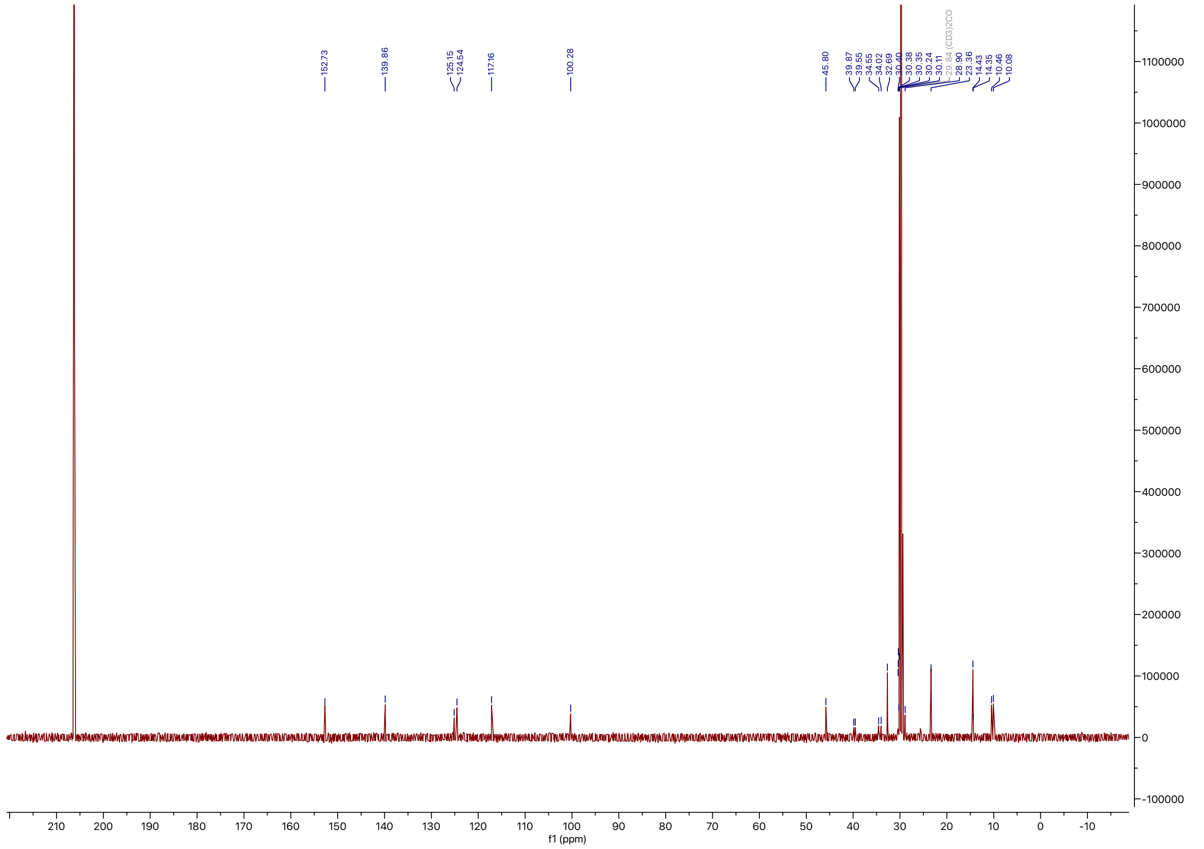

^{1}H-NMR (500 MHz) and ^{13}C-NMR (126 MHz) spectrum of 8f in Acetone-d_6 at 298 K.

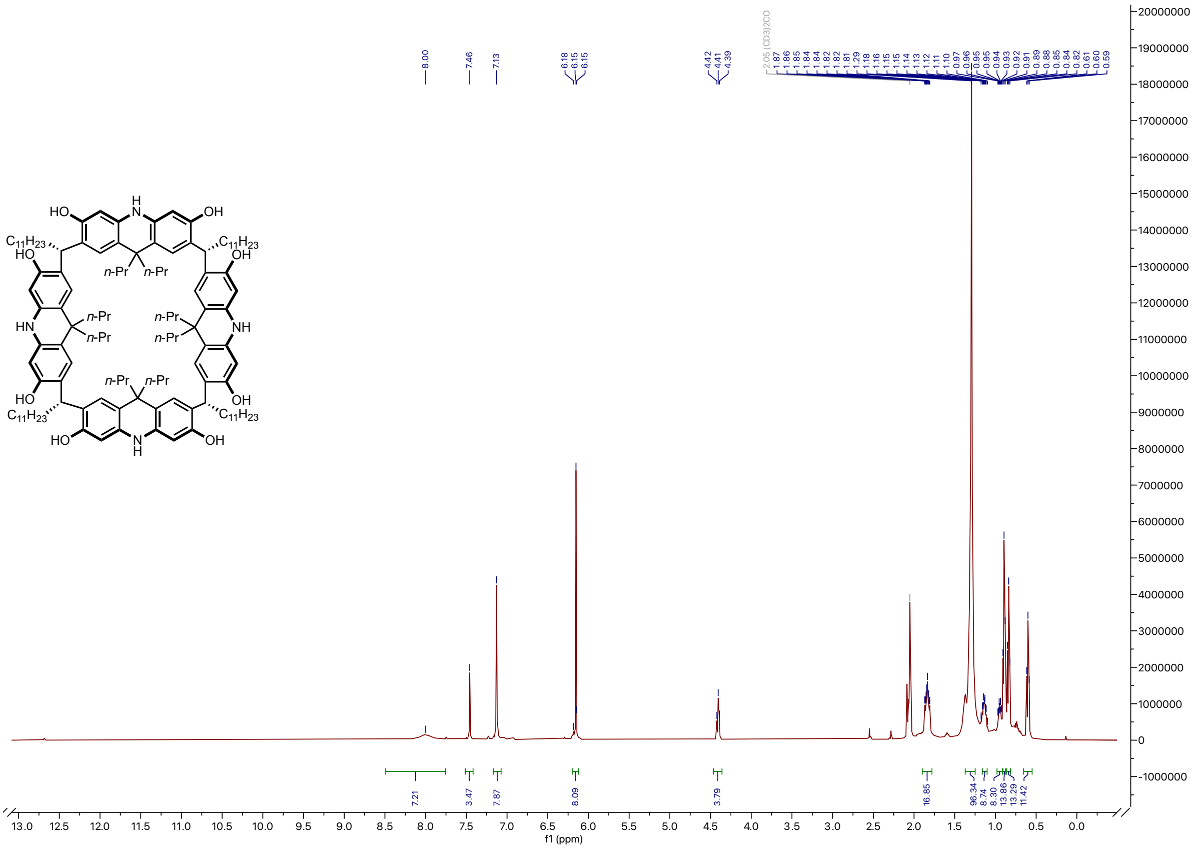

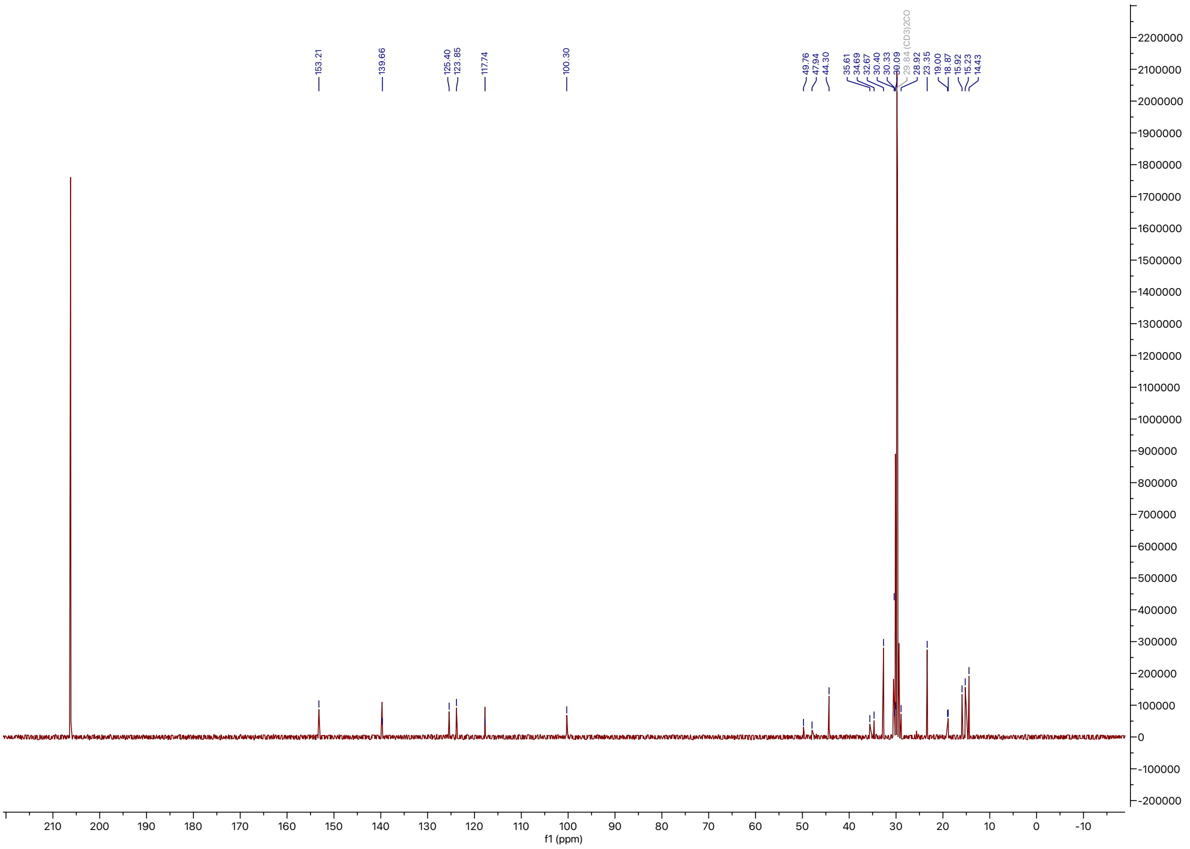

^{1}H-NMR (500 MHz) and ^{13}C-NMR (126 MHz) spectrum of 9 in Acetone-d_6 at 298 K.

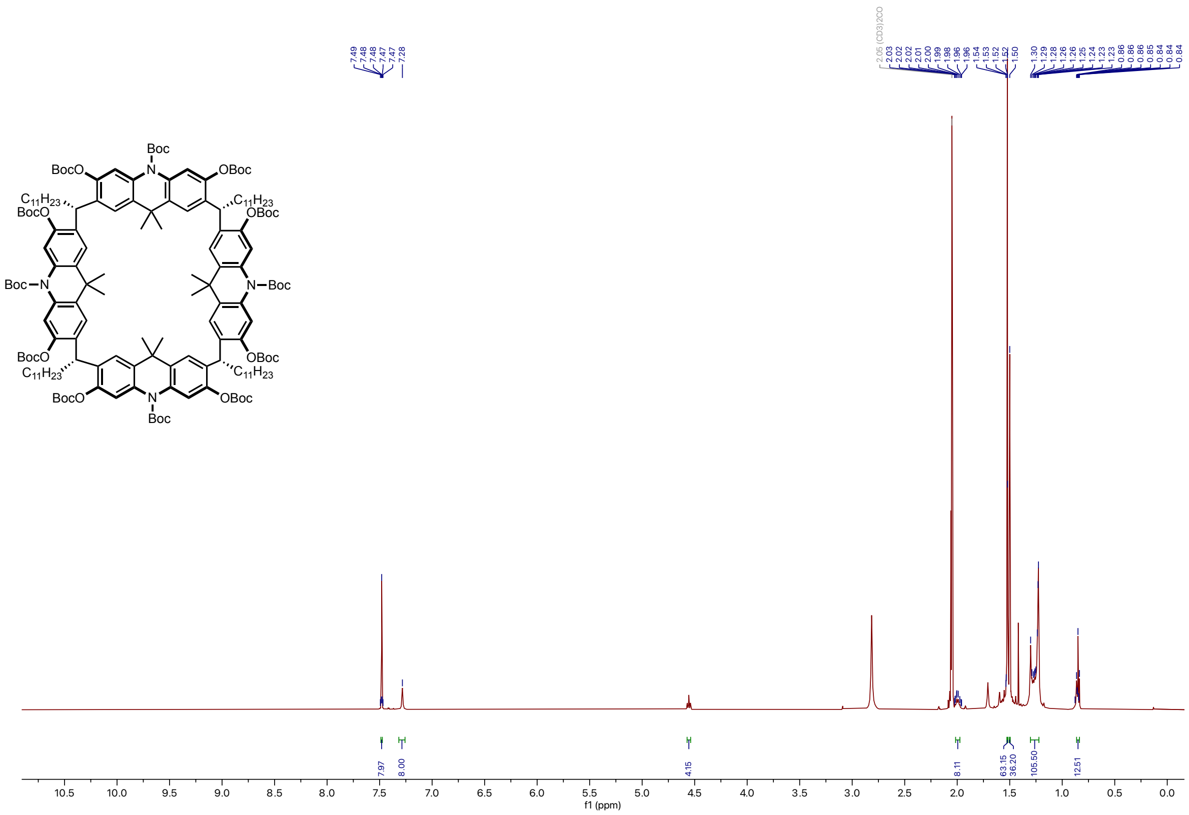

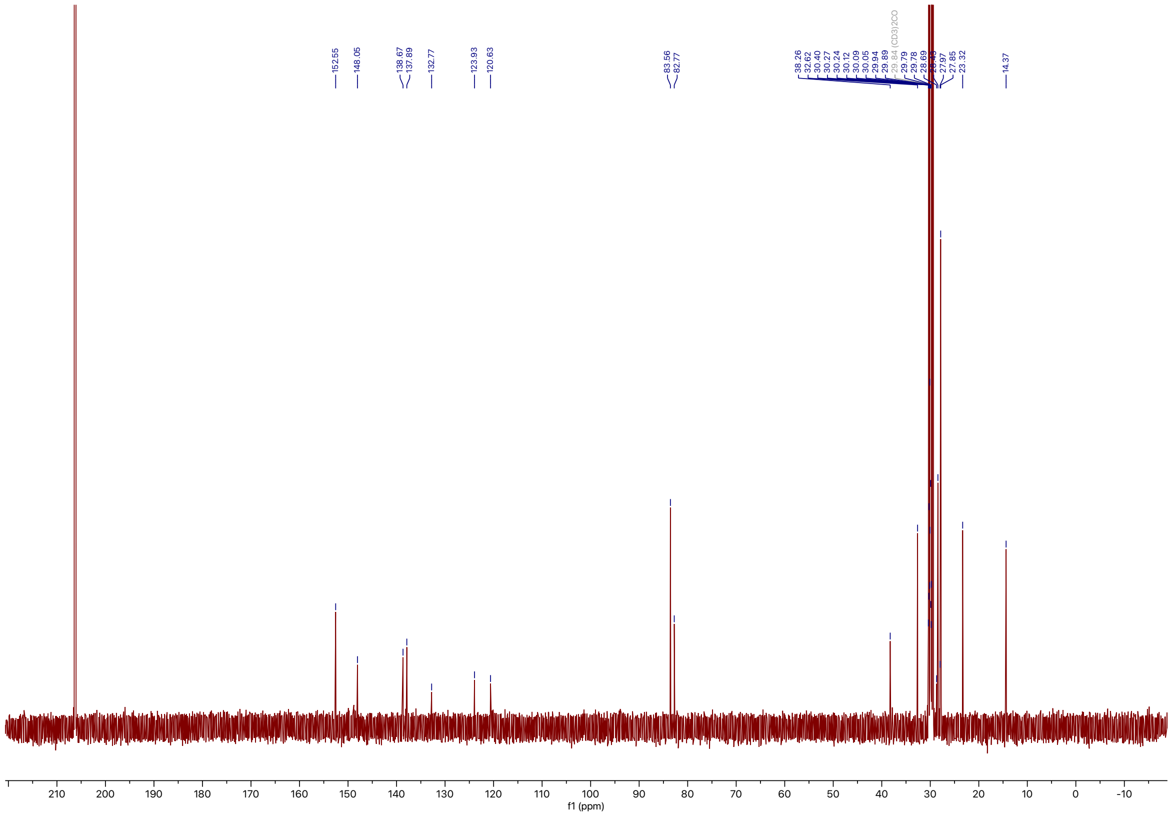

^{1}H-NMR (500 MHz) and ^{13}C-NMR (126 MHz) spectrum of 10 in Acetone-d_6 at 298 K.

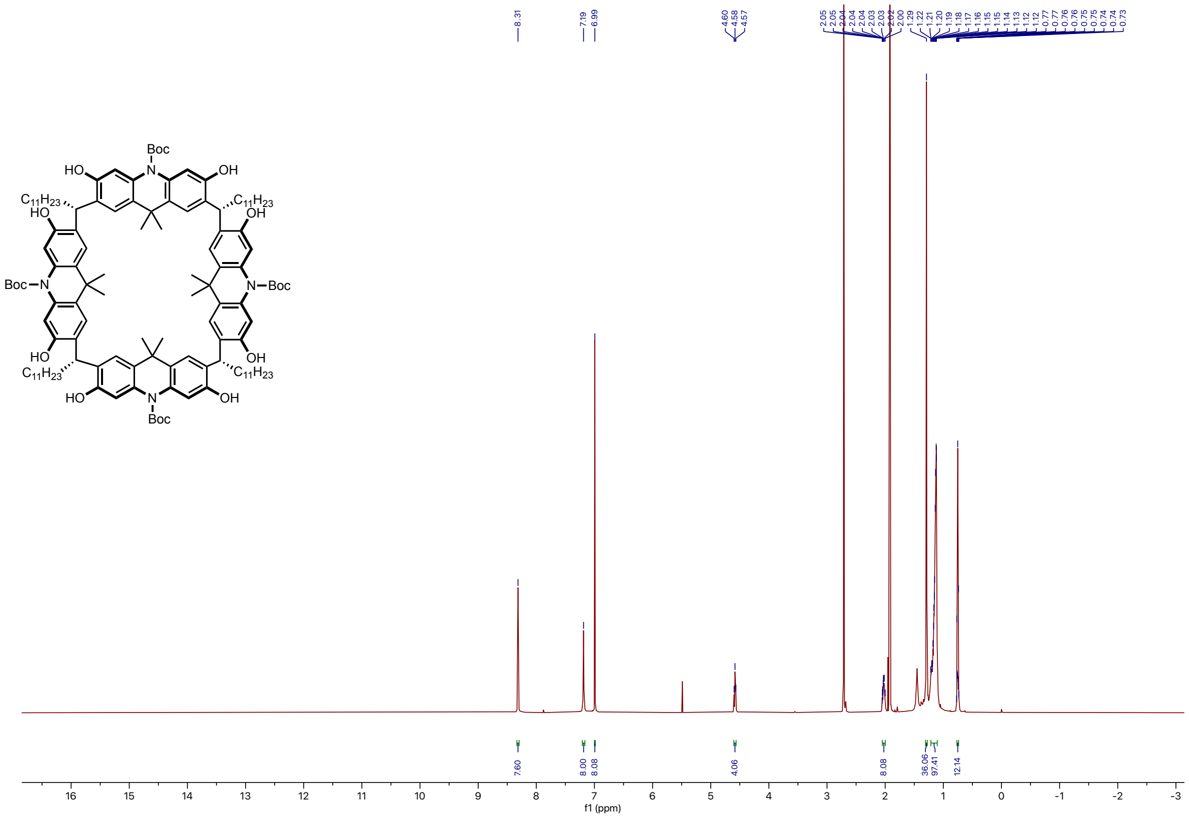

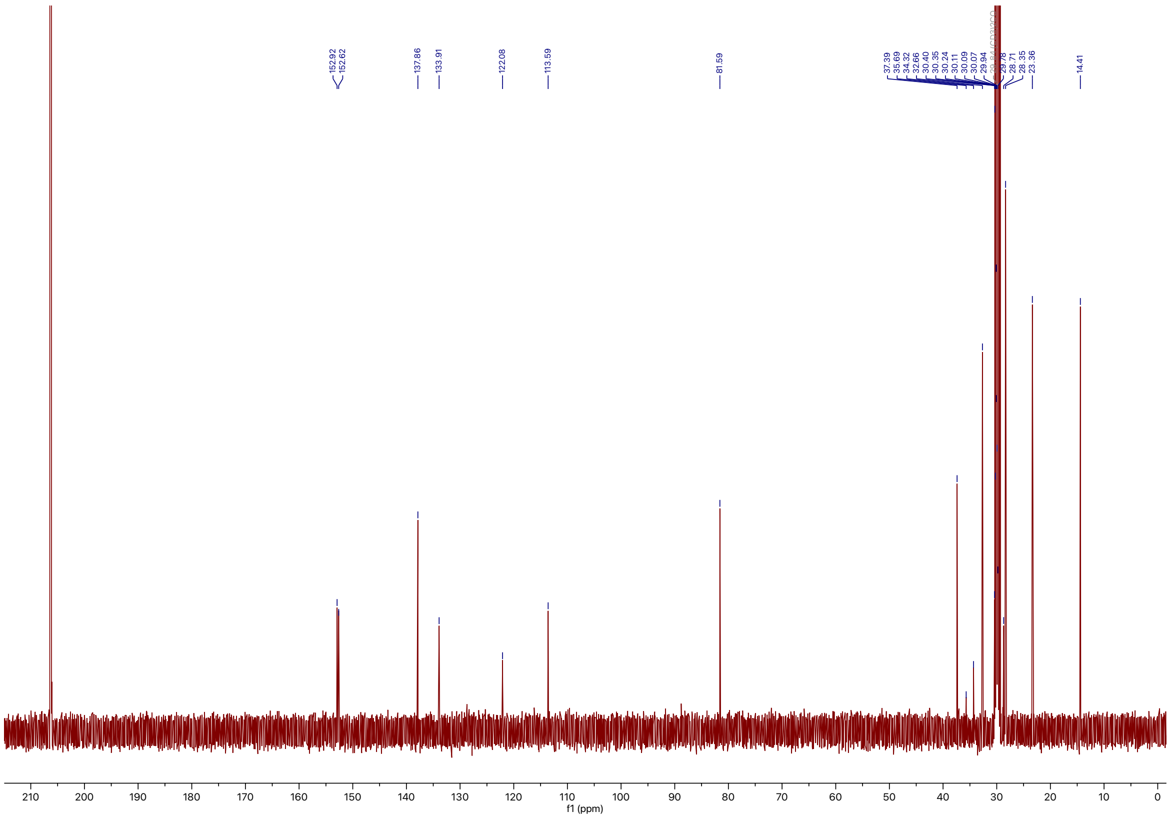

^{1}H-NMR (500 MHz) and ^{13}C-NMR (126 MHz) spectrum of 11 in Acetone-d_6 at 298 K.

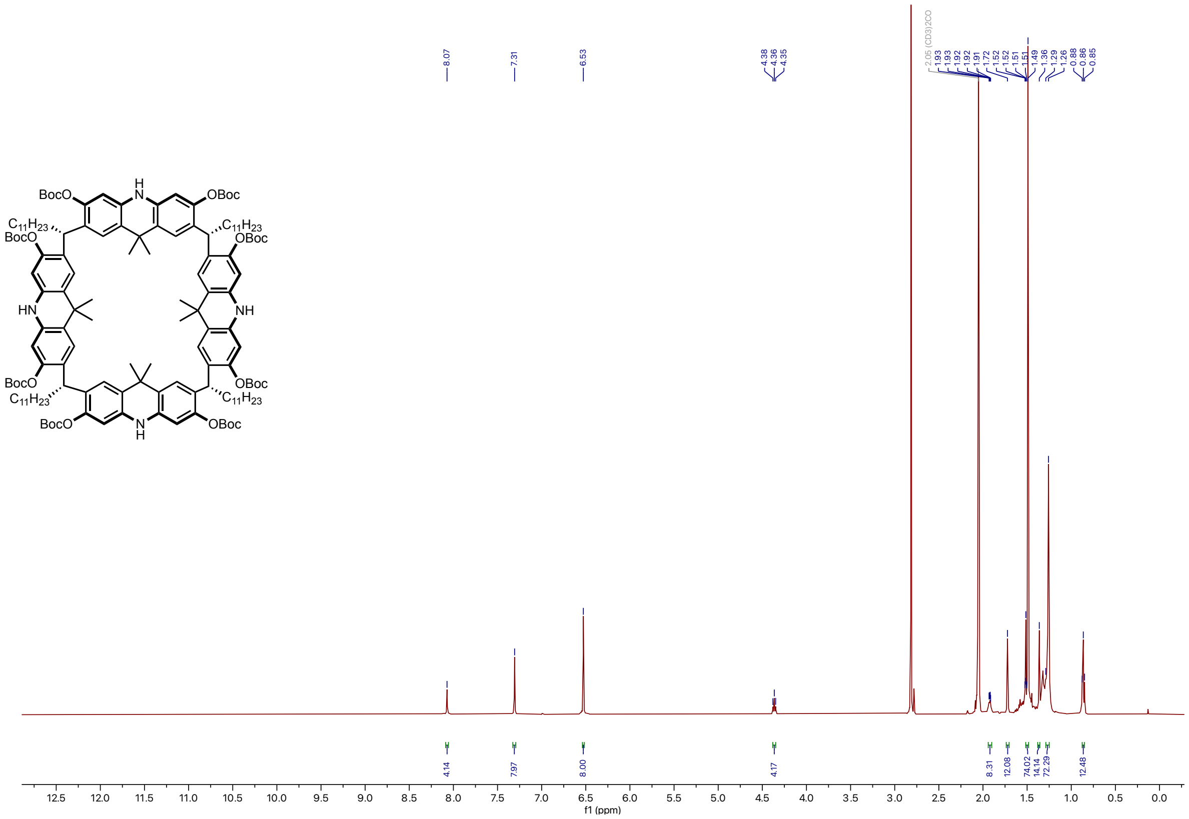

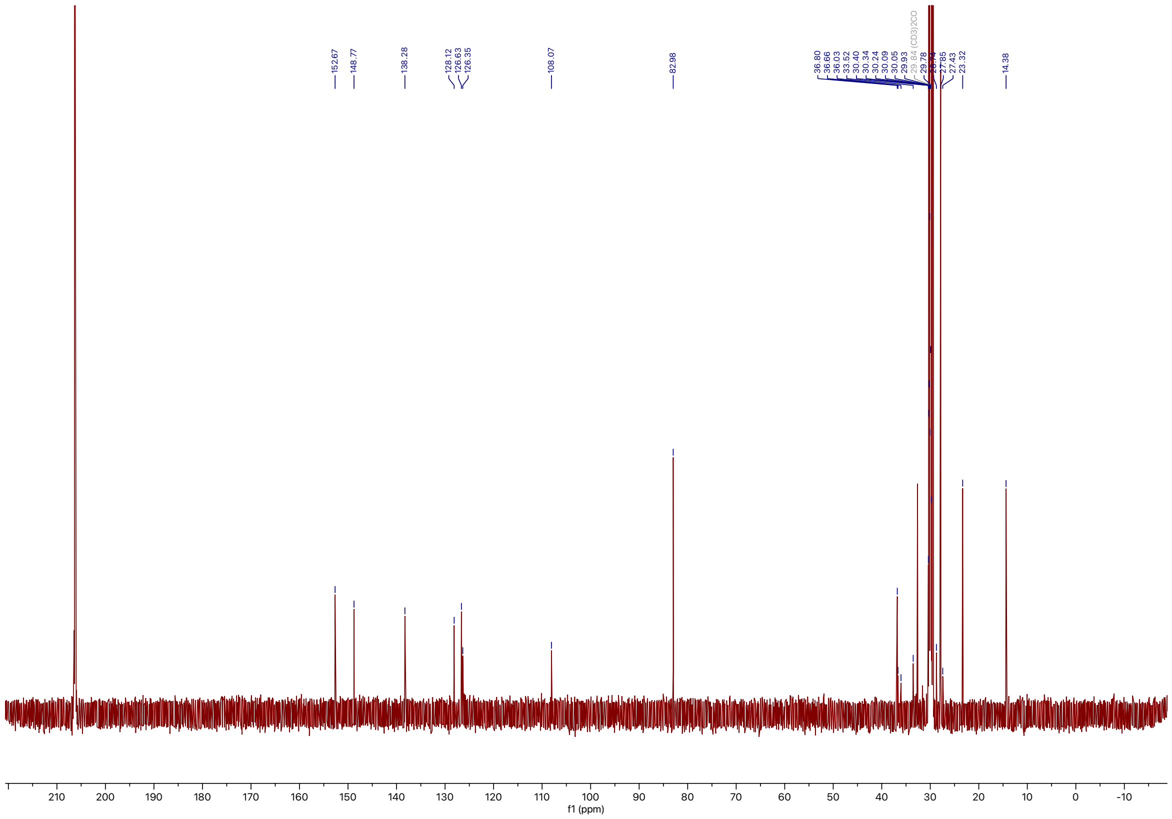

^{1}H-NMR (500 MHz) and ^{13}C-NMR (126 MHz) spectrum of 12 in Acetone-d_6 at 298 K.

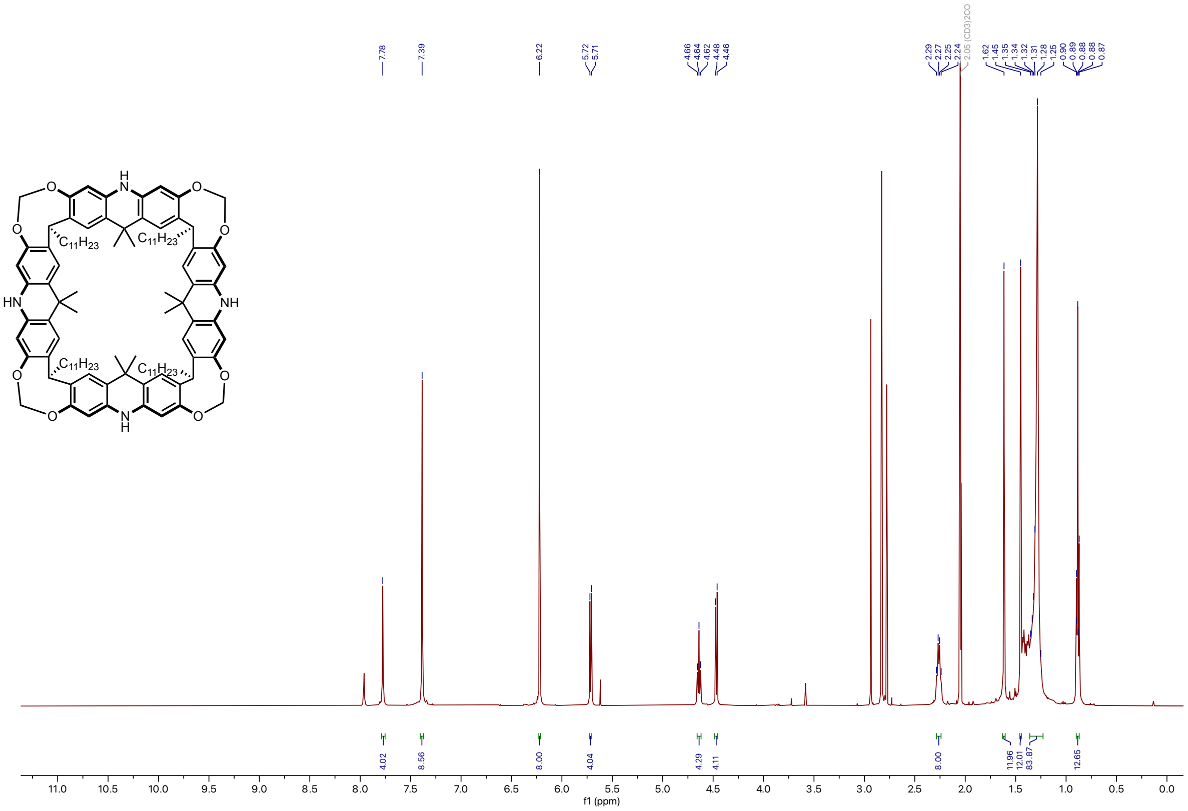

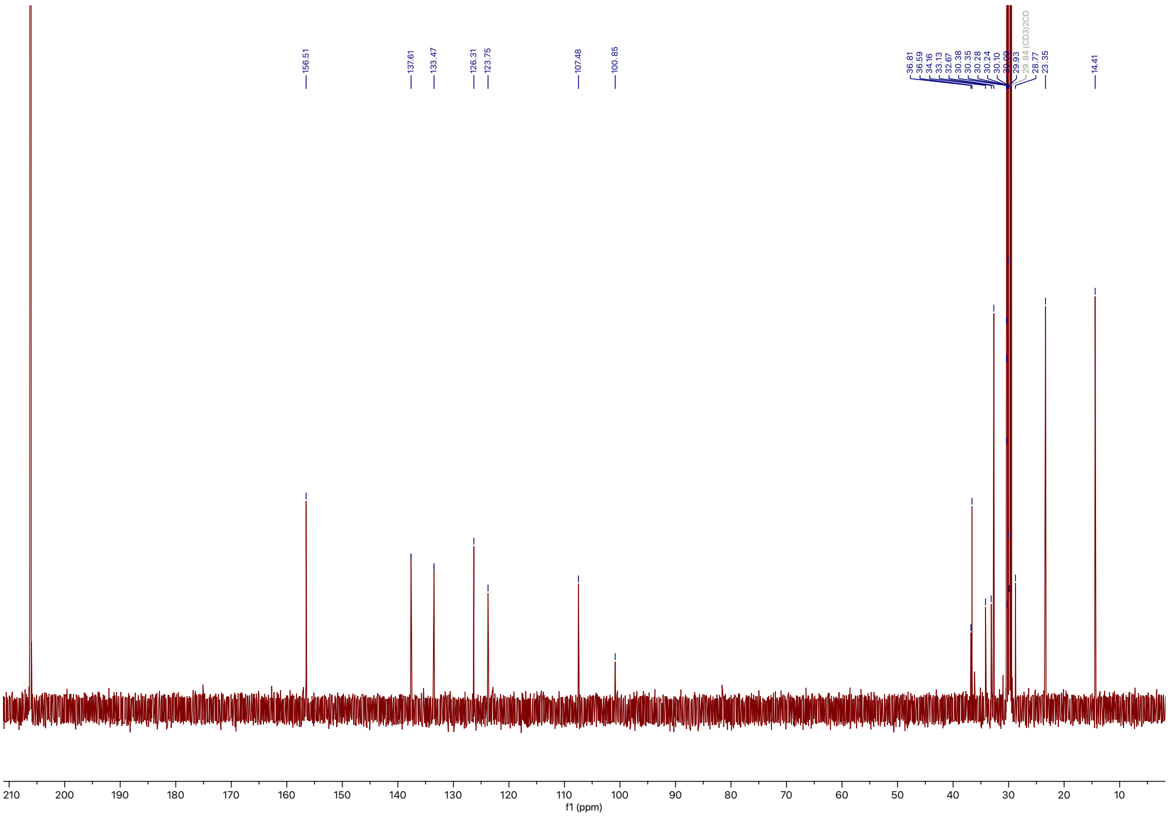

^{1}H-NMR (600 MHz), ^{13}C-NMR (151 MHz), and ^{19}F-NMR (470MHz, referenced with hexafluoroisopropanol), spectrum of 13 in Acetone-d_6 at 298 K.

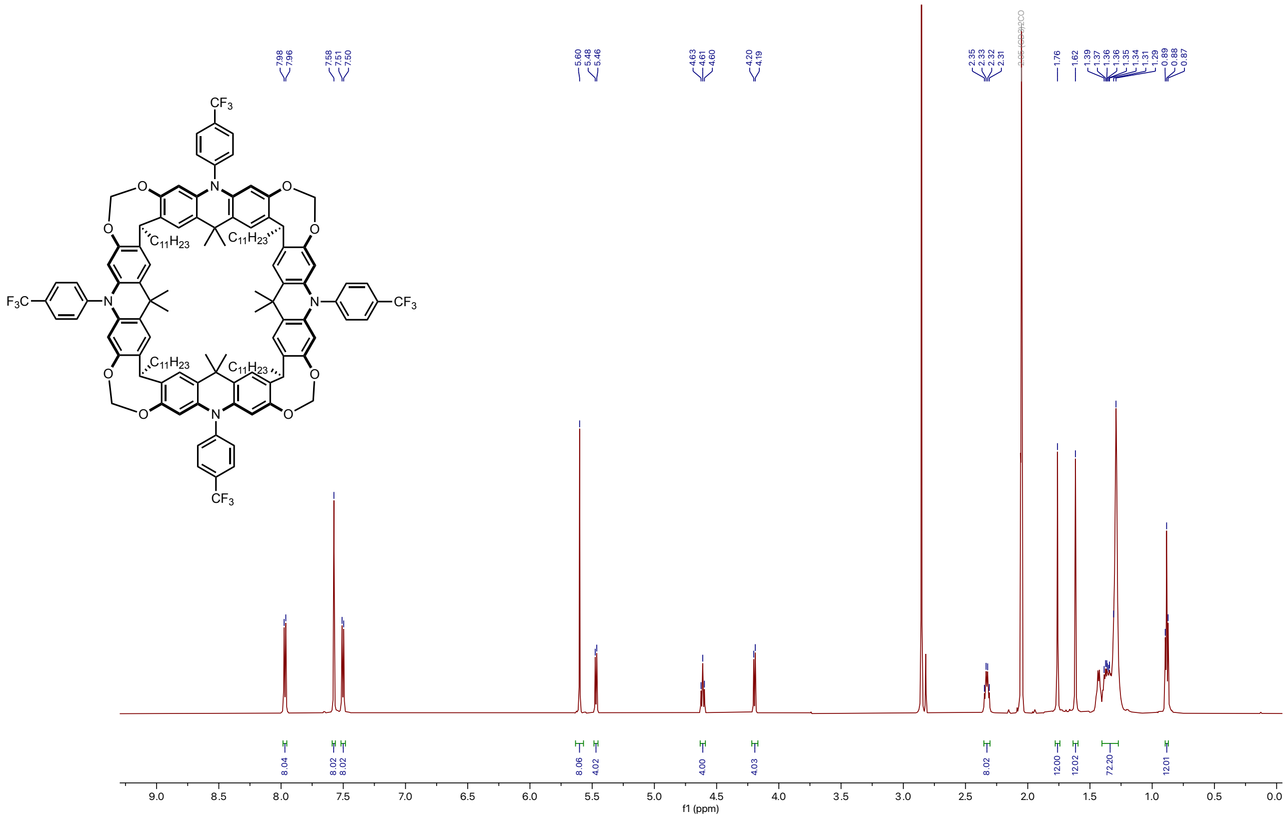

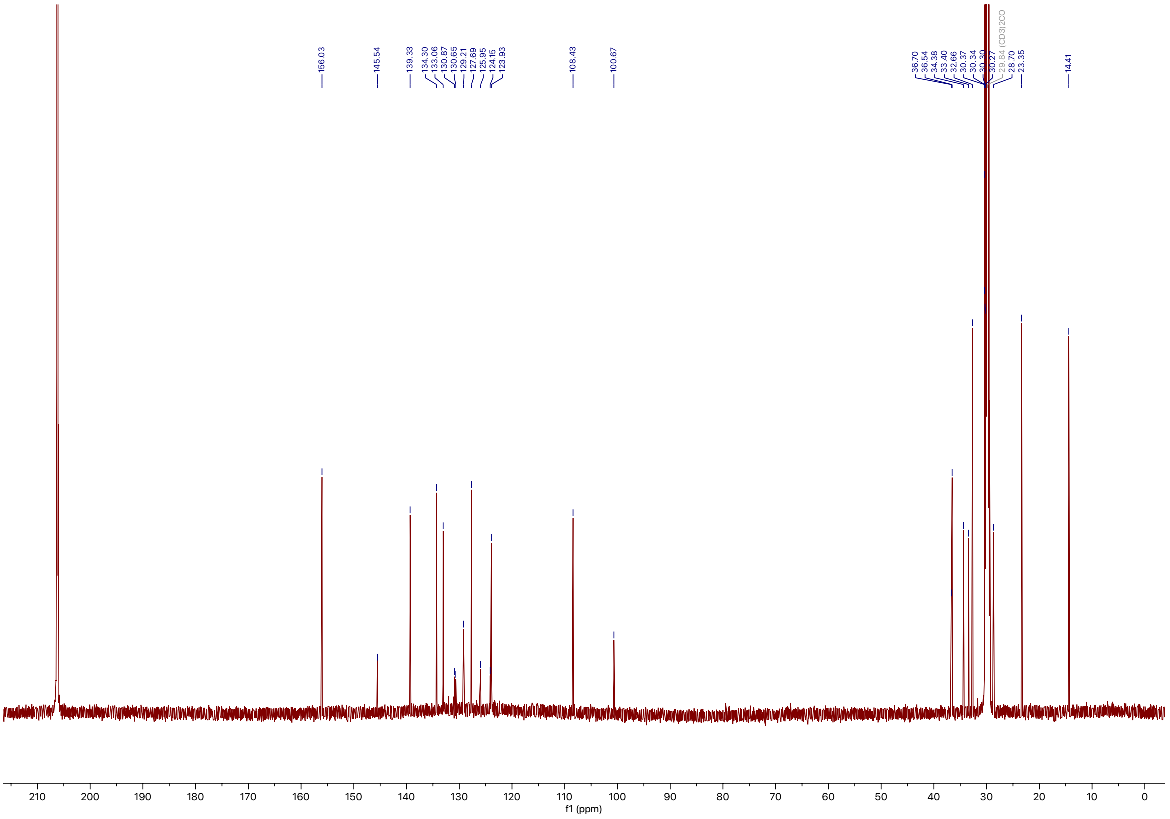

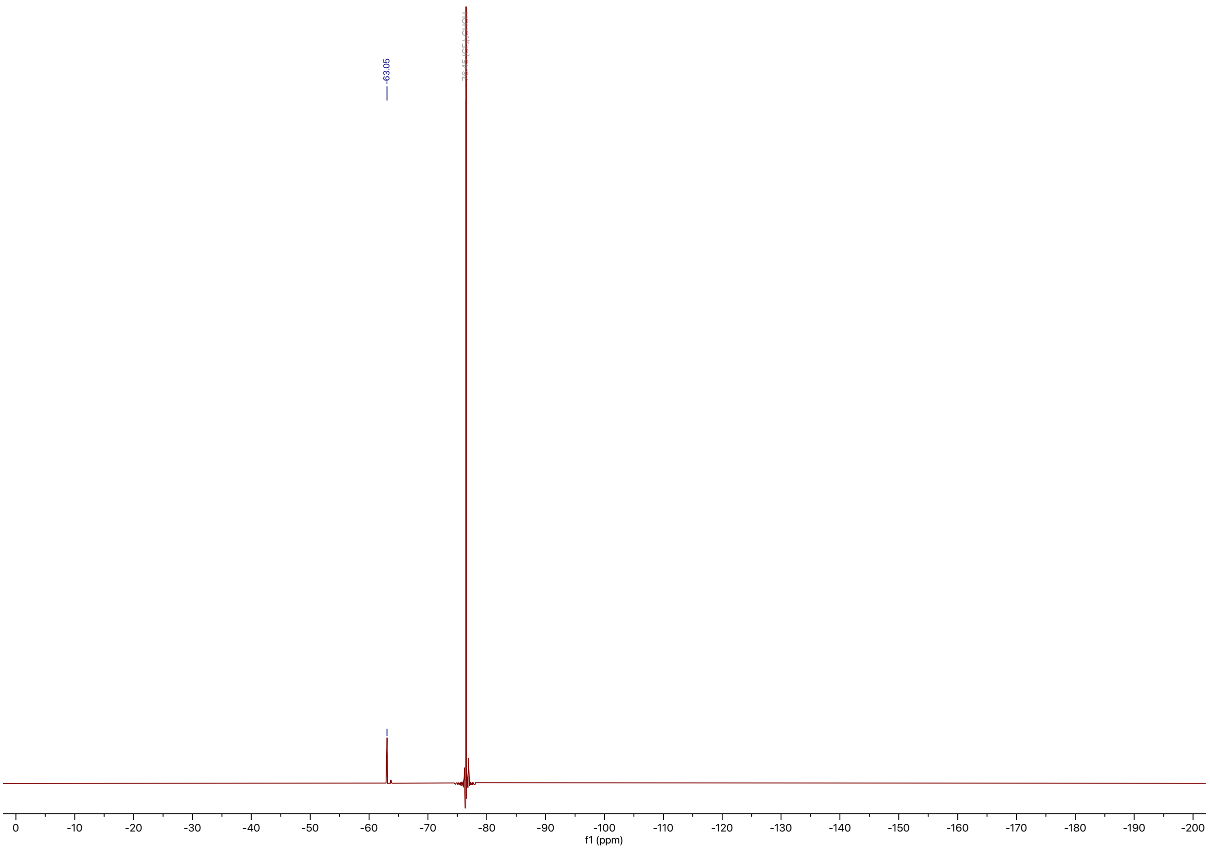
-63.05
-76.45 (CF3)2CHOH
0
-10
-20
-30
-40
-50
-60
-70
-80
-90
-100
-110
-120
-130
-140
-150
-160
-170
-180
-190
-200
f1 (ppm)

^{1}H-NMR (500 MHz) and ^{13}C-NMR (126 MHz) spectrum of 14 in Acetone-d_6 at 298 K.

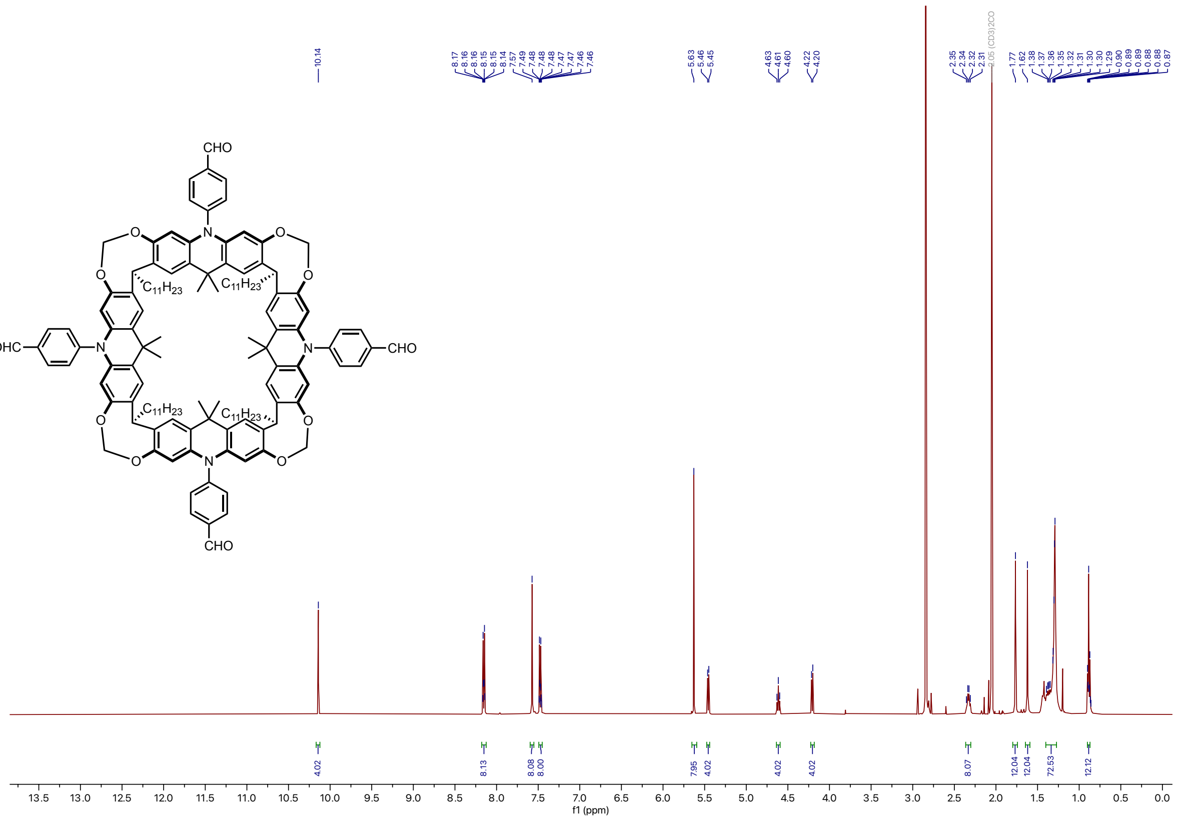

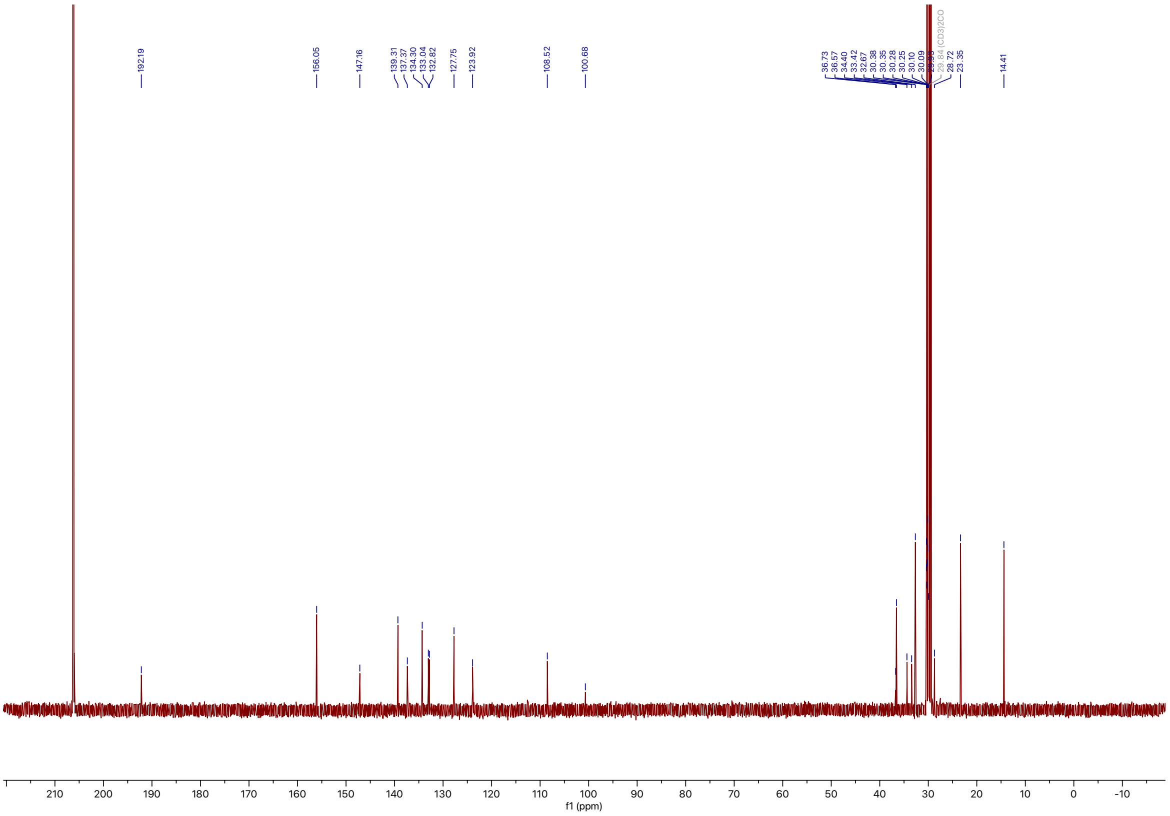

^{1}H-NMR (500 MHz) and ^{13}C-NMR (126 MHz) spectrum of 15 in Acetone-d_6 at 298 K.

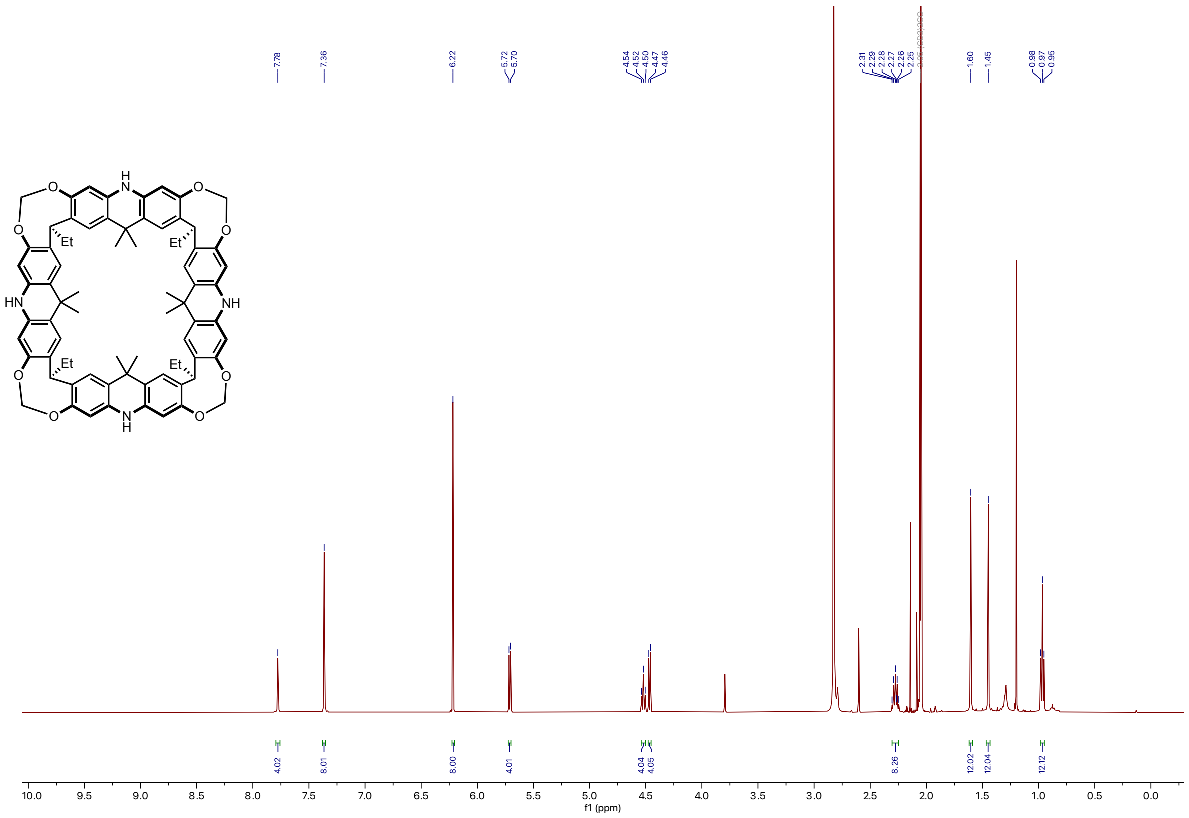

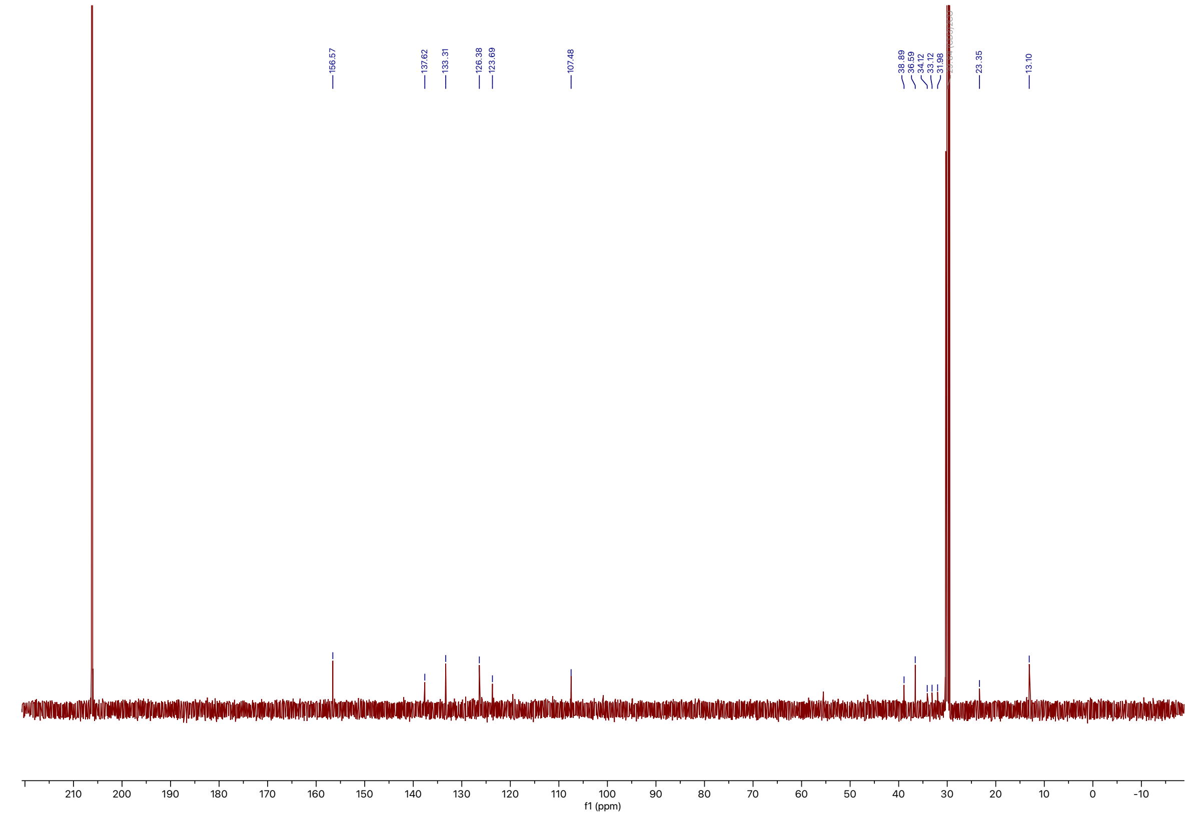

^{1}H-NMR (500 MHz) and ^{13}C-NMR (126 MHz) spectrum of 16 in CD_2Cl_2 at 298 K.

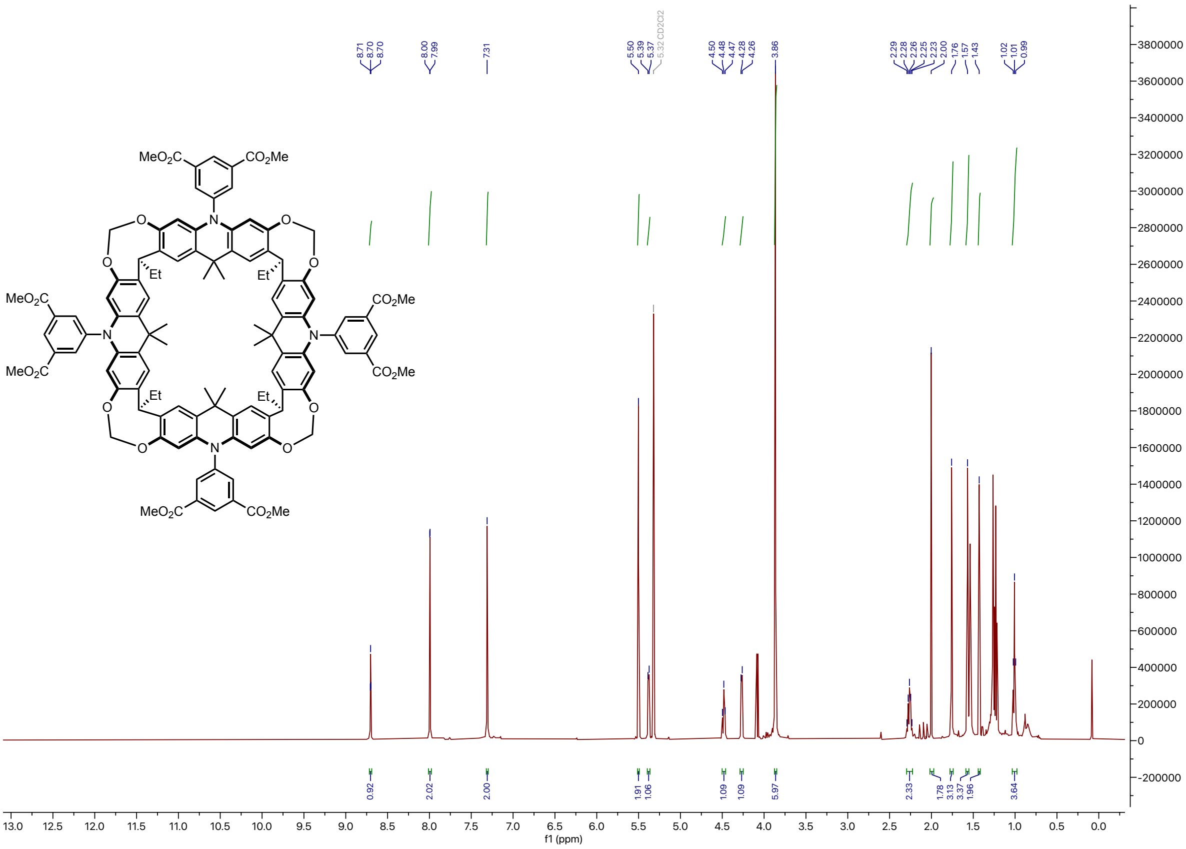

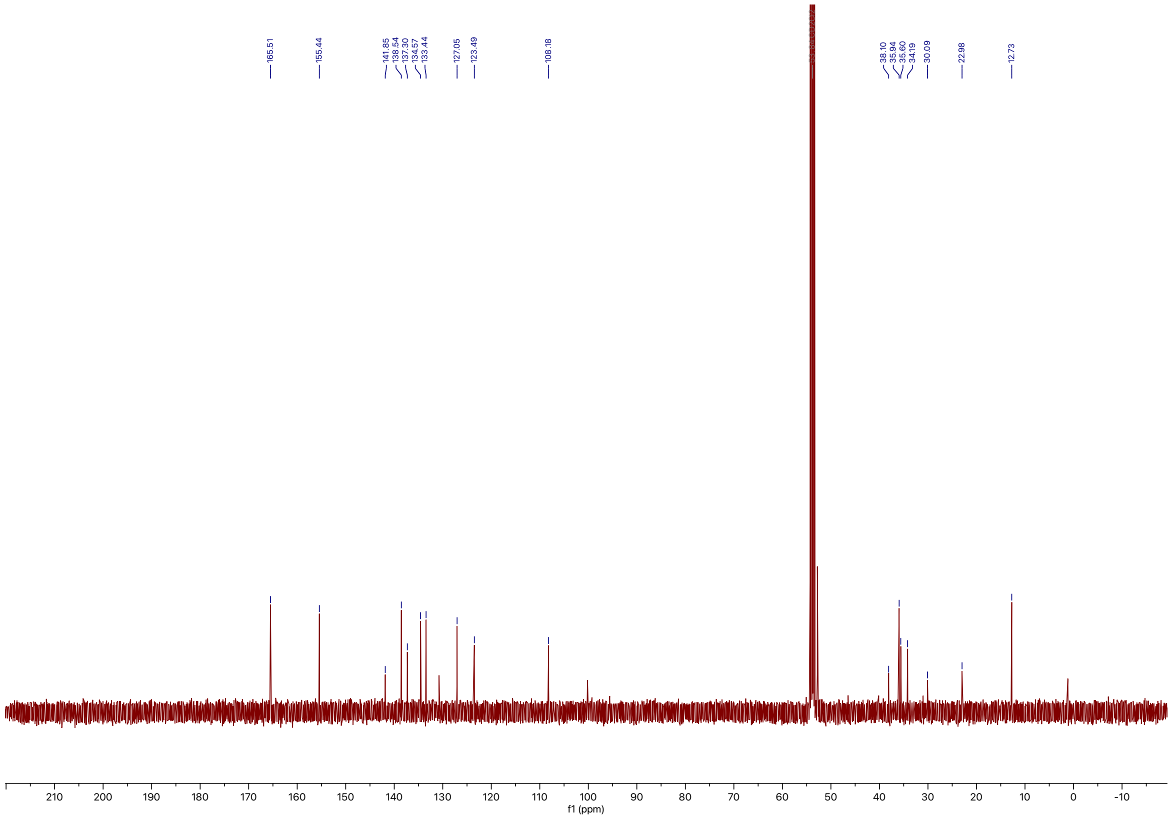

^{1}H-NMR (500 MHz) and ^{13}C-NMR (126 MHz) spectrum of 17 in Acetone-d_6 at 298 K.

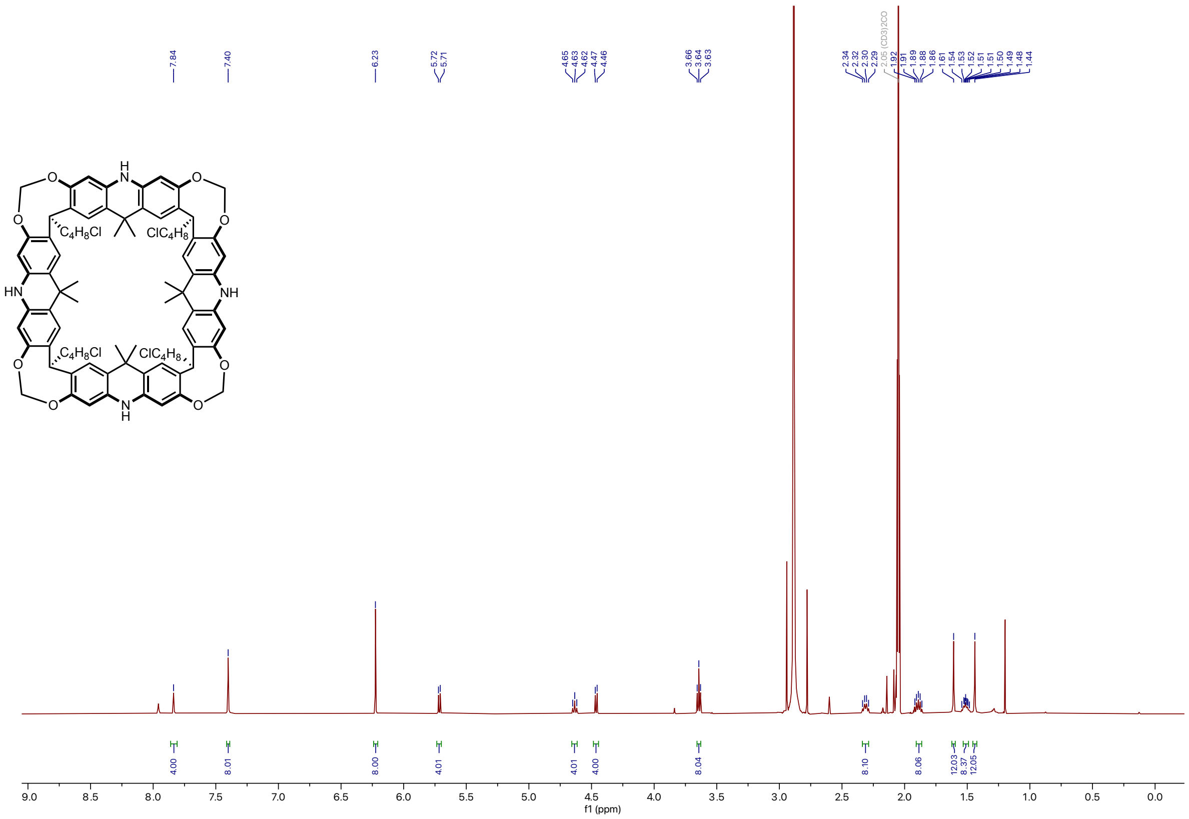

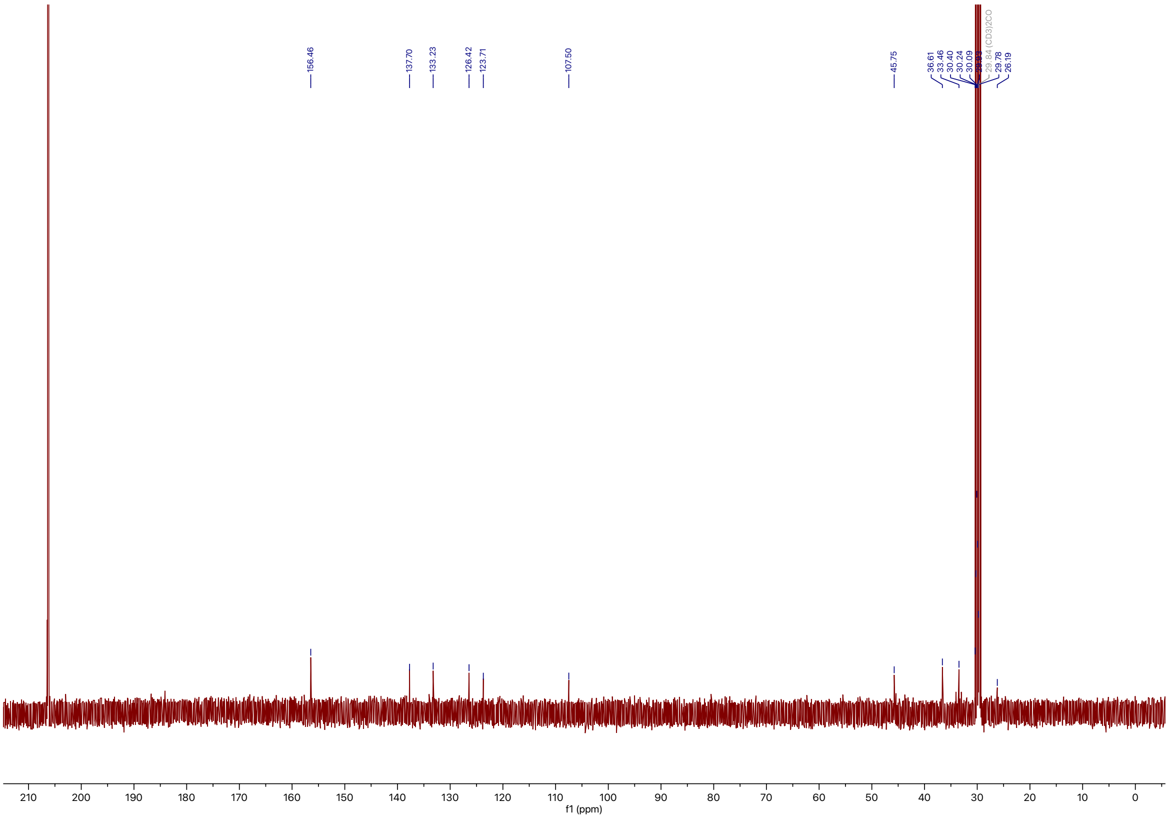

^{1}H-NMR (500 MHz) and ^{13}C-NMR (126 MHz) spectrum of 18 in MeOD-d_4 at 298 K.

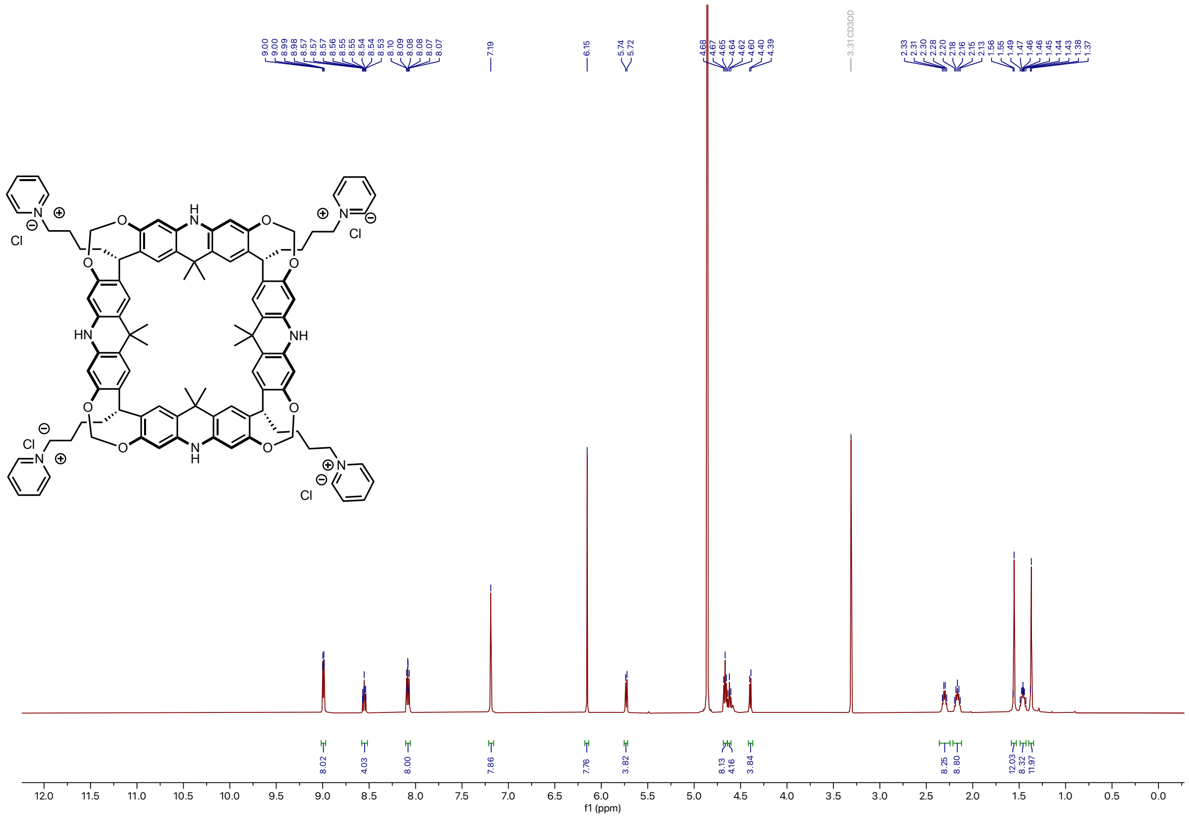

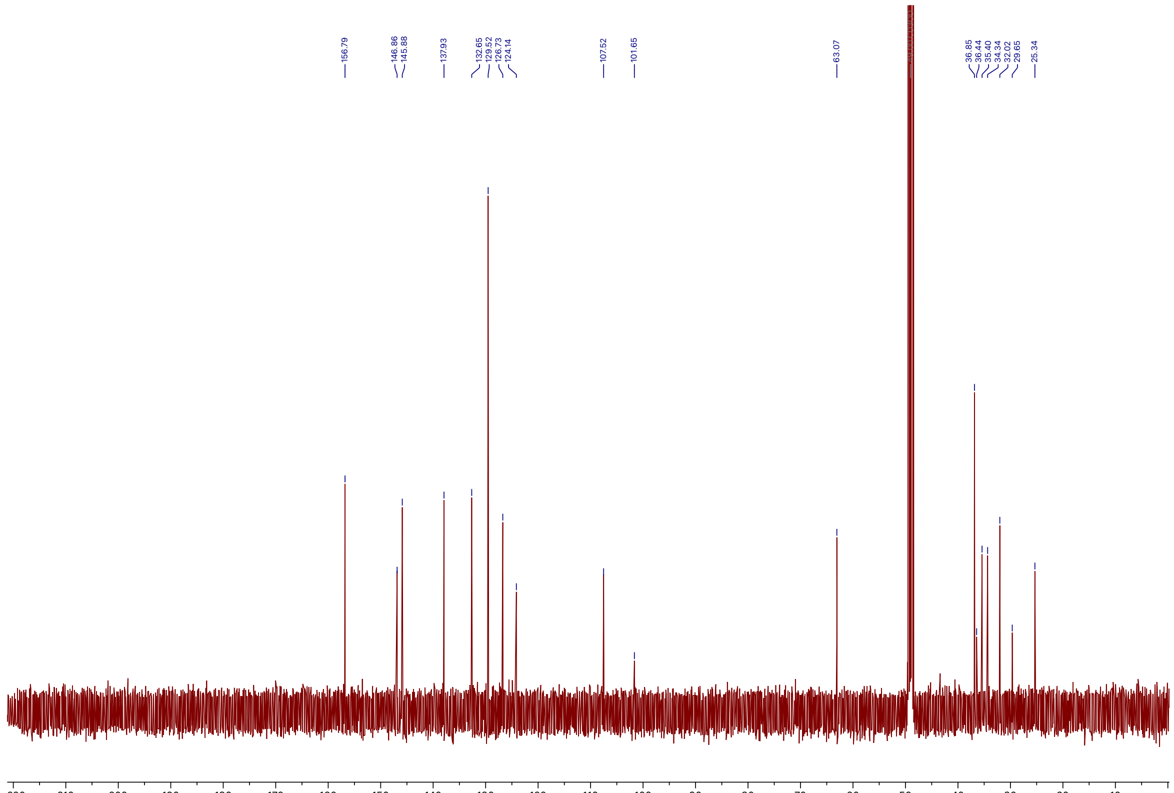

^{1}H-NMR (500 MHz) spectrum of 18 in D_2O/DMSO-d_6 (10 v%) at 298 K.

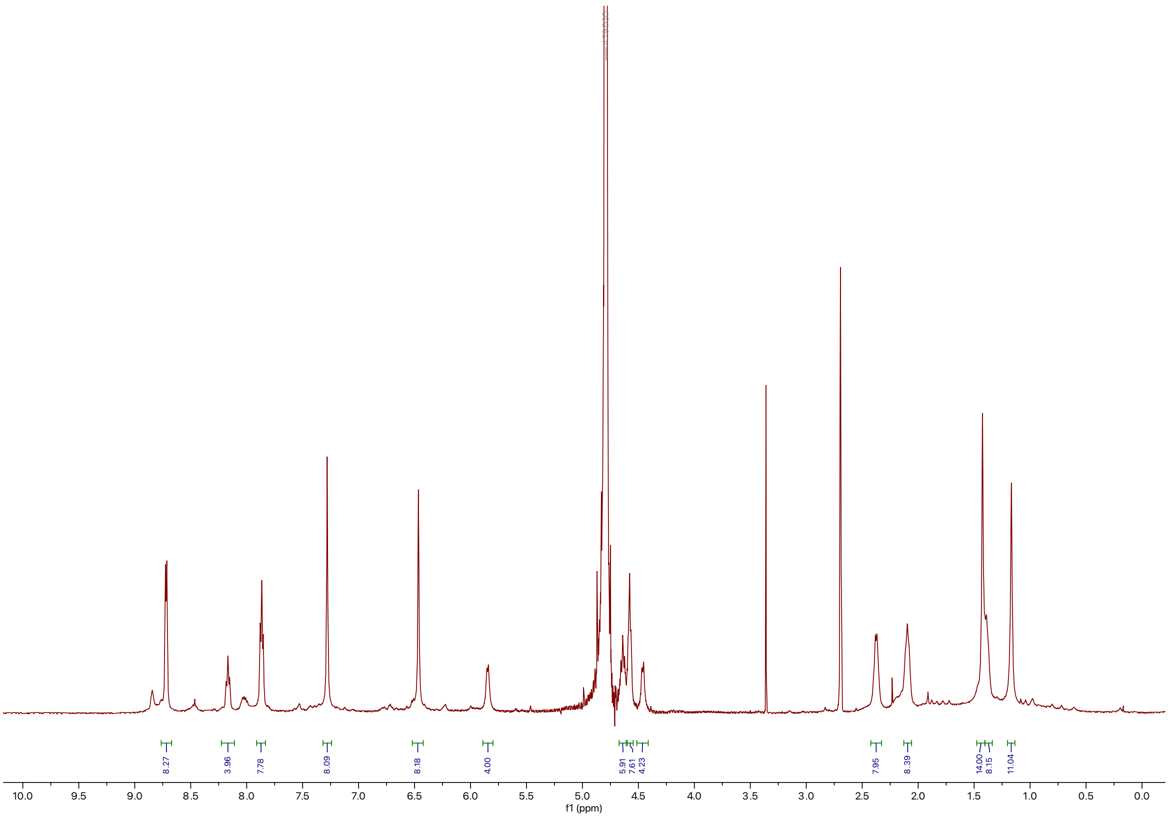

^{1}H-NMR (500 MHz) and ^{13}C-NMR (126 MHz) spectrum of 19 in MeOD-d_4 at 298 K.

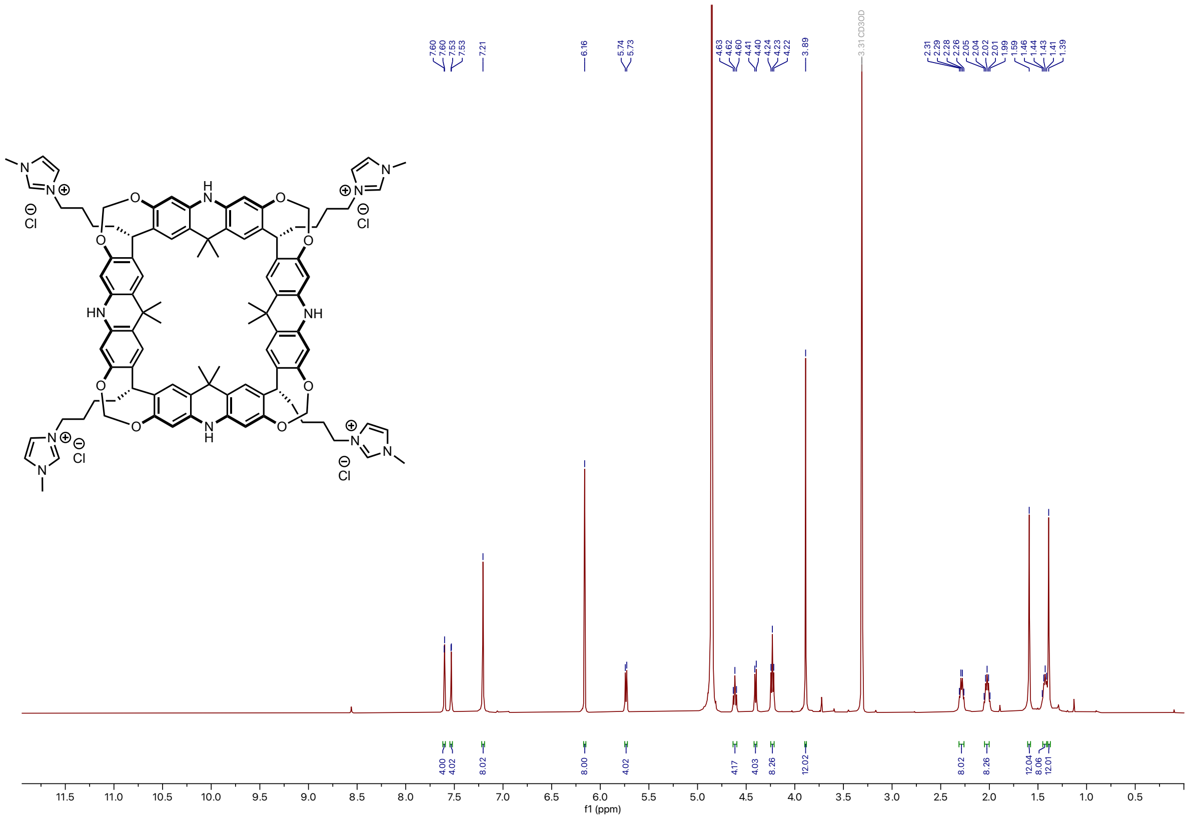

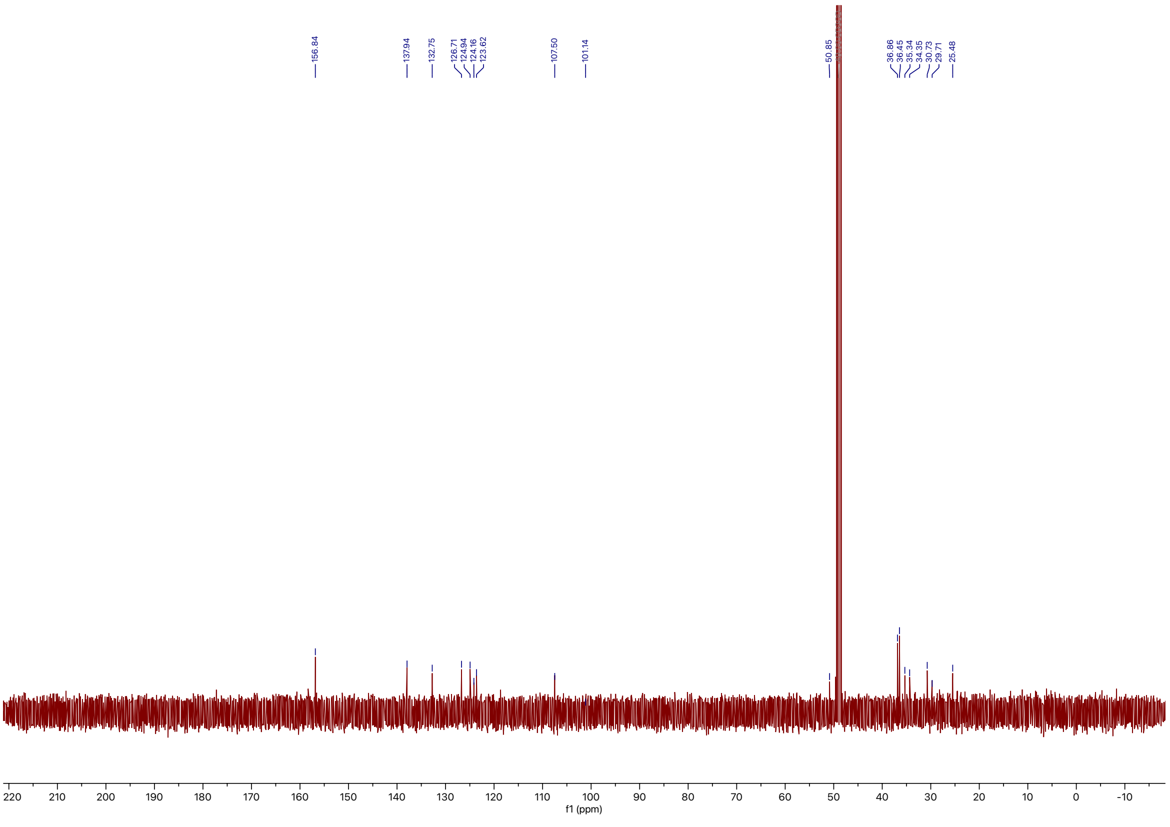

^{1}H-NMR (500 MHz) spectrum of 19 in D_2O/DMSO-d_6 (10v%) at 298 K.

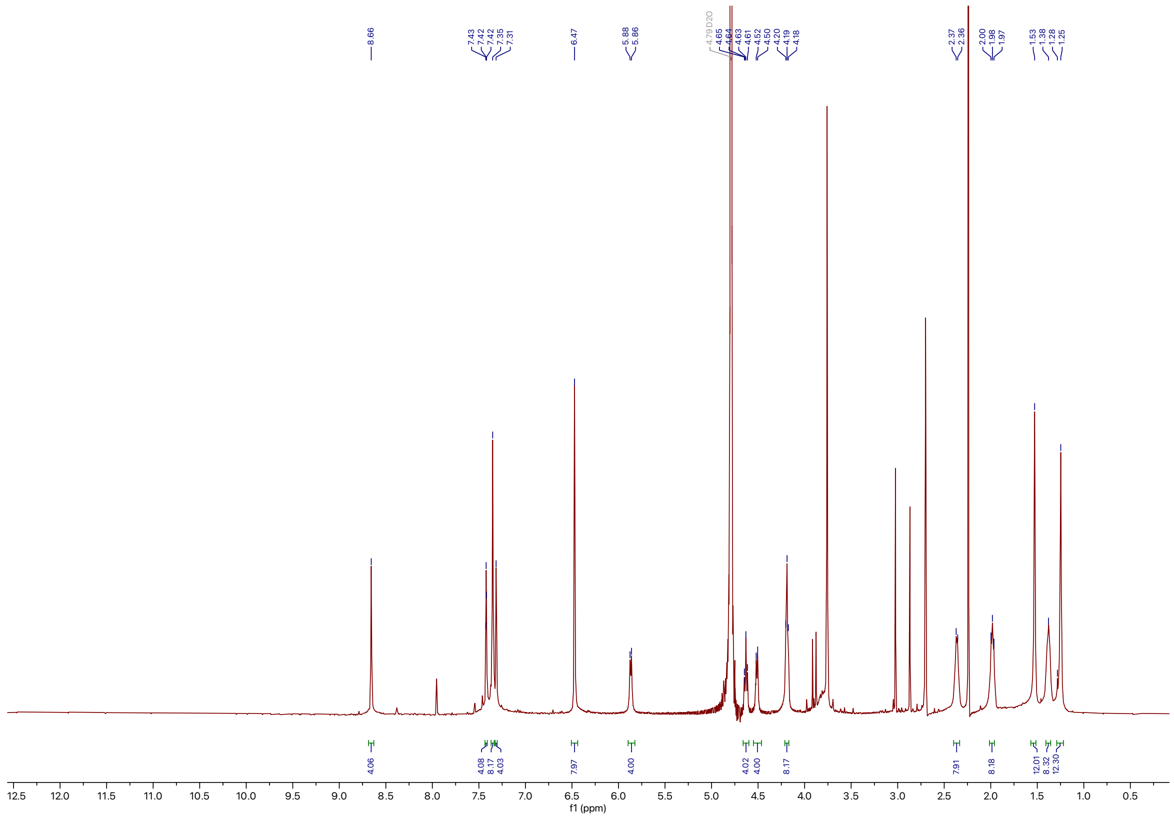

8. References

(1) Gottlieb, H. E.; Kotlyar, V.; Nudelman, A. NMR Chemical Shifts of Common Laboratory Solvents as Trace Impurities. *J. Org. Chem.* **1997**, *62* (21), 7512-7515. DOI: 10.1021/jo971176v

(2) Pfeuffer-Rooschuz, J.; Heim, S.; Prescimone, A.; Tiefenbacher, K. Megalo-Cavitands: Synthesis of Acridane[4]arenes and Formation of Large, Deep Cavitands for Selective C70 Uptake. *Angew. Chem. Int. Ed.* **2022**, *61* (42), e202209885. DOI: 10.1002/anie.202209885.

(3) Chen, H. C.; Chen, S. H. Diffusion of crown ethers in alcohols. *J. Phys. Chem.* **1984**, *88* (21), 5118-5121.
